# Phosphotungstic acid as a novel acidic catalyst for carbohydrate protection and glycosylation†

**DOI:** 10.1039/c9ra06170c

**Published:** 2019-10-21

**Authors:** Jyun-Siao Chen, Arumugam Sankar, Yi-Jyun Lin, Po-Hsun Huang, Chih-Hsiang Liao, Shen-Shen Wu, Hsin-Ru Wu, Shun-Yuan Luo

**Affiliations:** Department of Chemistry, National Chung Hsing University Taichung 402 Taiwan syluo@dragon.nchu.edu.tw; Taichung Municipal Feng Yuan Senior High School Taichung 420 Taiwan; National Hsinchu Girls' Senior High School Hsinchu 300 Taiwan; Instrumentation Center, National Tsing Hua University, MOST Hsinchu 300 Taiwan

## Abstract

This work demonstrates the utilization of phosphotungstic acid (PTA) as a novel acidic catalyst for carbohydrate reactions, such as per-*O*-acetylation, regioselective *O*-4,6 benzylidene acetal formation, regioselective *O*-4 ring-opening, and glycosylation. These reactions are basic and salient during the synthesis of carbohydrate-based bioactive oligomers. Phosphotungstic acid's high acidity and eco-friendly character make it a tempting alternative to corrosive homogeneous acids. The various homogenous acid catalysts were replaced by the phosphotungstic acid solely for different carbohydrate reactions. It can be widely used as a catalyst for organic reactions as it is thermally stable and easy to handle. In our work, the reactions are operated smoothly under ambient conditions; the temperature varies from 0 °C to room temperature. Good to excellent yields were obtained in all four kinds of reactions.

## Abstract 2

This work demonstrates the utilization of PTA as an acidic catalyst in primary reactions of carbohydrate. PTA can be widely used as a catalyst for carbohydrate chemistry. The reactions are executed at 0 °C or room temperature in good yields.

## Graphical abstract

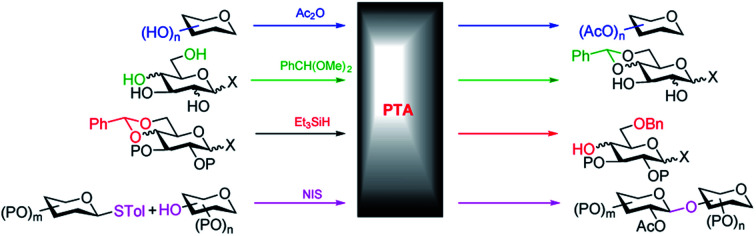

## Introduction

Carbohydrates are present throughout living organisms and are structurally richer than other biopolymers.^1^ They are involved in a vast range of crucial biological operations and are potential drug targets.^2,3^ They are available as mixtures from natural sources.^4^ Pure forms of carbohydrates are essential for understanding their biological activity^5*a*^ and disease-related information.^5*b*^ A few strategies for minimizing the number of steps and achieving oligosaccharides in good yields through chemical synthesis are available.^6^ The synthesis of oligosaccharides requires the preparation of glycosyl acceptors and glycosyl donors,^7*a*^ which upon glycosylation, produce stereo-selective and regio-selective glycosidic bonds.^7*b*^ Hence, new techniques for synthesizing oligosaccharides are essential.^8^

Acetylation is a common reaction in carbohydrate chemistry. It can proceed in the presence of Cu(OTf)_2_,^9*a*^ HClO_4_,^9*b*^ FeCl_3_,^10*a*^ V(O)(OTf)_3,_^10*b*^ pyridine,^11*a*^ Dy(OTf)_3,_^11*b*^ and Zn_4_(OCOCF_3_)_6_O.^11*c*^ However, mineral acid is corrosive and pyridine is harmful,^11*d*^ so new ways to conduct this reaction are sought. 4,6-*O*-benzylidene acetal is an important moiety in the synthesis of polysaccharides as one can reductively open the acetal either at *O*-4 or *O*-6 position of monosaccharide,^12*a*^ by tuning of the reaction conditions and give a free hydroxyl group selectively at C4 or C6. It could be installed by using acid or base^12*b*^ such as camphorsulfonic acid (CSA),^12*c*^*p*-toluenesulfonic acid (TsOH),^12*d*^ SnCl_4_^12*e*^ and TCT.^12*f*^ Nevertheless, the formation of 4,6-*O*-benzylidene acetals and its yields are limited by side-products and the requirement of severe acidic medium.^12*g*^ Selective *O*-4 ring opening is also an important reaction of carbohydrates as it affords free hydroxyl group at C-4 position. This reaction proceeds in the presence of trifluoroacetic acid and triethylsilane^12*h*,*i*^ or sodium cyanoborohydride.^13^ However, this reaction provides low yield^14*a*^ and sodium cyanoborohydride is dangerous,^14*b*^ so new mediators for this reaction must be found to increase the yield. Finally, the glycosylation reaction requires an acid catalyst to build the oligosaccharides and it requires acid catalysts such as FeCl_3_/C,^15*a*^ Cu(OTf)_2_,^15*b*^ BF_3_·OEt_2_ or TMSOTf.^15*c*^ However, no common acid catalyst for all of these reactions has been identified.

Hence, we would like to replace these different homogenous corrosive acid catalysts by using a sole acid catalyst for important reactions of carbohydrates. Furthermore, that acid catalyst must be eco-friendly, acidity must be strong enough, should not be corrosive and affordable. Fortunately, all of this requirements were fulfilled by the phosphotungstic acid. Thence, in this work, we have used phosphotungstic acid (H_3_[P(W_3_O_10_)_4_]) catalyst for basic reactions namely per-*O*-acetylation, regioselective *O*-4,6 benzylidene acetal formation, regioselective *O*-4 reductive ring-opening, and glycosylation reactions. All of these reactions were underwent smoothly in the presence of PTA under ambient reaction condition.

PTA is a heteropolyacid,^16*a*^ and a solid inorganic substance,^16*b*^ it is following Keggin's structure.^16*c*^ Phosphotungstic acid is non-toxic,^17*a*^ eco-friendly,^17*b*^ and the strongest heteropoly acid^18*a*^ than common mineral acid such as H_2_SO_4_, HCl, and HNO_3_.^18*b*^ It has a good chemical stability, thermal stability, reuse, and recycling.^19–21^ Phosphotungstic acid extensively used in well known organic reactions, including Prins cyclization,^22*a*^ Claisen–Schmidt condensation,^22*b*^ Schiff-bases synthesis,^23^ Pinacol-pinacolone rearrangements,^24^ Beckman rearrangements,^25^ bio-diesel synthesis in industries,^26*a*–*c*^ and quinolone synthesis.^27^

**Fig. 1:**
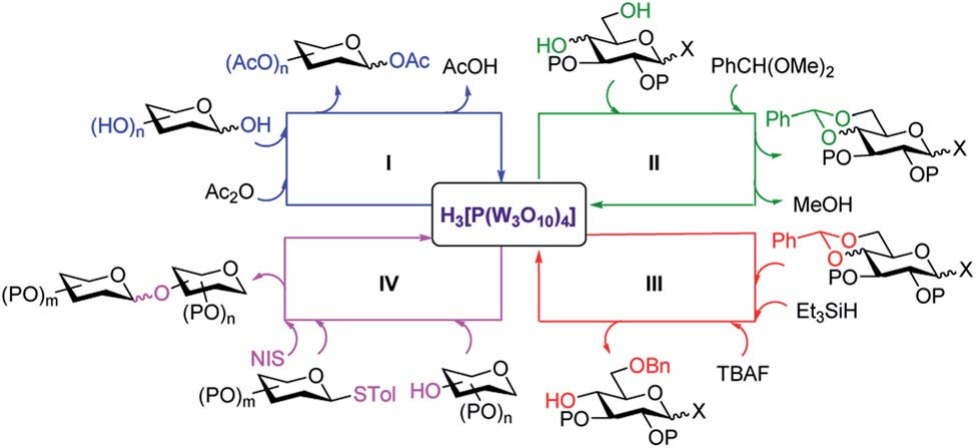
Reactions of carbohydrates with phosphotungstic acid as the catalyst.

Neverthless, its impact in carbohydrate field is quite low and a few groups have been employed it as a catalyst for carbohydrate reactions.^28*a*,*b*^ To the best of our knowledge, the applications of PTA in carbohydrates field is inadequate. Accordingly, our group extended the utilization of PTA to carbohydrates principle reactions, and obtaining good to excellent yields and will discuss in next part. These general reactions are depicted in Fig. 1.

## Results and discussion

### Acetylation

Table 1 presents the results of the phosphotungstic acid-catalyzed per-*O*-acetylation reaction. d-glucose 1a was used as the initial substrate to test phosphotungstic acid as a catalyst of per-*O*-acetylation. To test per-*O*-acetylation, the reaction was conducted under solvent-free conditions with d-glucose 1a, phosphotungstic acid (0.05 equiv.) and acetic anhydride (10 equiv.) in an atmosphere of nitrogen at room temperature. After 24 hours, the starting material was disappeared on the TLC plate and the desired product d-glucose pentaacetate 2a was obtained in 66% (*α*/*β* = 5/1) isolated yield (Table 1, entry 1). In an effort to increase the yield of the per-*O*-acetylated product, the amount of phosphotungstic acid was reduced to 0.01 equivalent. This change remarkably increased the yield of 2a to 88% (*α*/*β* = 5/1) without changing other reaction conditions (Table 1, entry 2). When 0.02 equivalent of phosphotungstic acid was used, the desired product 2a was afforded in good yield 93% (*α*/*β* = 5/1) (Table 1, entry 3).

**Table 1:** Phosphotungstic acid catalyzed per-*O*-acetylation

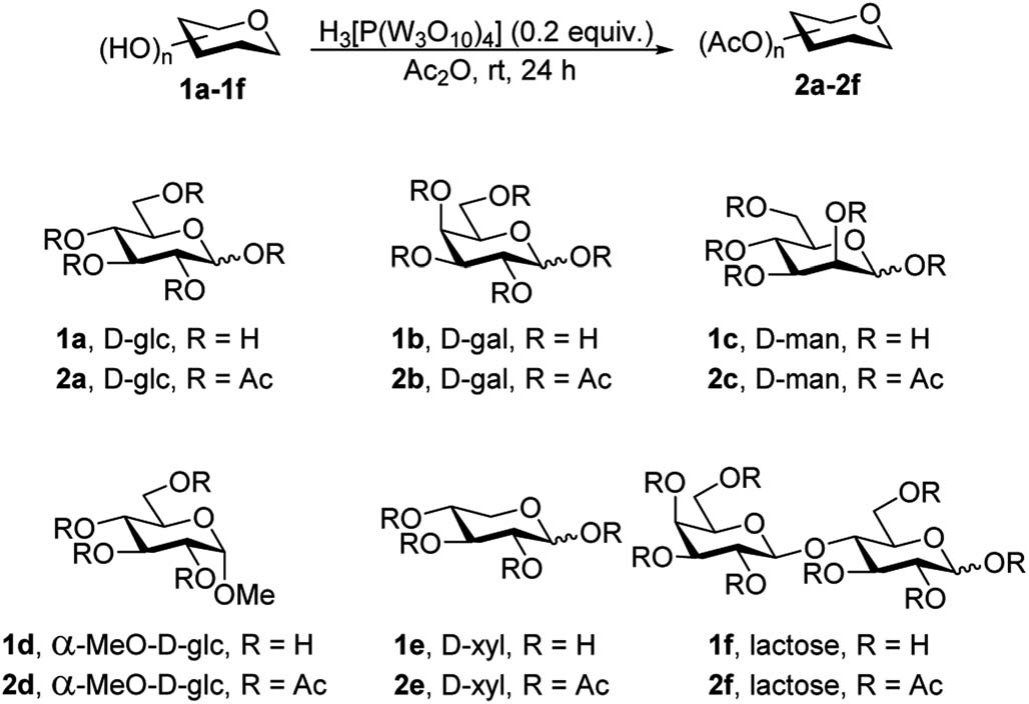				
Entry	SM	H_3_[P(W_3_O_10_)_4_] (equiv.)	Product (*α*/*β*)	Yield
1	1a	0.05	2a (5/1)	66%
2	1a	0.01	2a (5/1)	88%
3	1a	0.02	2a (5/1)	93%
4	1b	0.02	2b (5/1)	81%
5	1c	0.02	2c (*α* only)	88%
6	1d	0.02	2d (*α* only)	88%
7	1e	0.02	2e (5/1)	99%
8	1f	0.02	2f (5/1)	96%

Therefore, under the improved reaction conditions of per-*O*-acetylation by phosphotungstic acid, different substrates were screened. Unprotected sugars, such as d-galactose 1b, d-mannose 1c, methyl alpha-d-glucopyranoside 1d, d-xylose 1e and lactose 1f also participated in the reaction, which proceeded reaction smoothly under the same reaction conditions and producing d-galactose pentaacetate 2b in 81% (*α*/*β* = 5/1), d-mannose pentaacetate 2c in 88% (*α* only 1), 2d in 88% (*α* only), d-xylose tetraaacetate 2e in 99% (*α*/*β* = 5/1) and lactose octaacetate 2f in 96% (*α*/*β* = 5/1) yields (Table 1, entries 4–8). The various carbohydrates underwent reactions smoothly with acetic anhydride in the presence of phosphotungstic acid and the yields of acetylated products were excellent. These favorable findings help in solving problems such as the separation and corrosiveness of high-boiling-point liquid acids. Importantly, we developed a new method for per-*O*-acetylation with comparatively low cost PTA and it afforded good yield as Cu(OTf)_2_ and HClO_4_ wich are expensive catalysts.^9*a*,*b*^

### Acetalization

Phosphotungstic acid is used for the regioselective 4,6-*O*-benzylidene acetalization, as presented in Table 2. The reaction of 4,6-*O*-benzylidene acetalization started with 1d, phosphotungstic acid (0.5 equiv.), and benzaldehyde dimethyl acetal (2.0 equiv.) in acetonitrile. After 12 hours, the desired product 4a was obtained in 71% isolated yield (Table 2, entry 1). Initial attempts were made to find a suitable equivalent of phosphotungstic acid to enhance the yields of the desired product 4a. Notably, lowering the amount of phosphotungstic acid catalyst to 0.2 equivalent reduced the yield of 4a to 58% (Table 2, entry 2).

**Table 2:** Phosphotungstic acid catalyzed benzylidene acetal formation

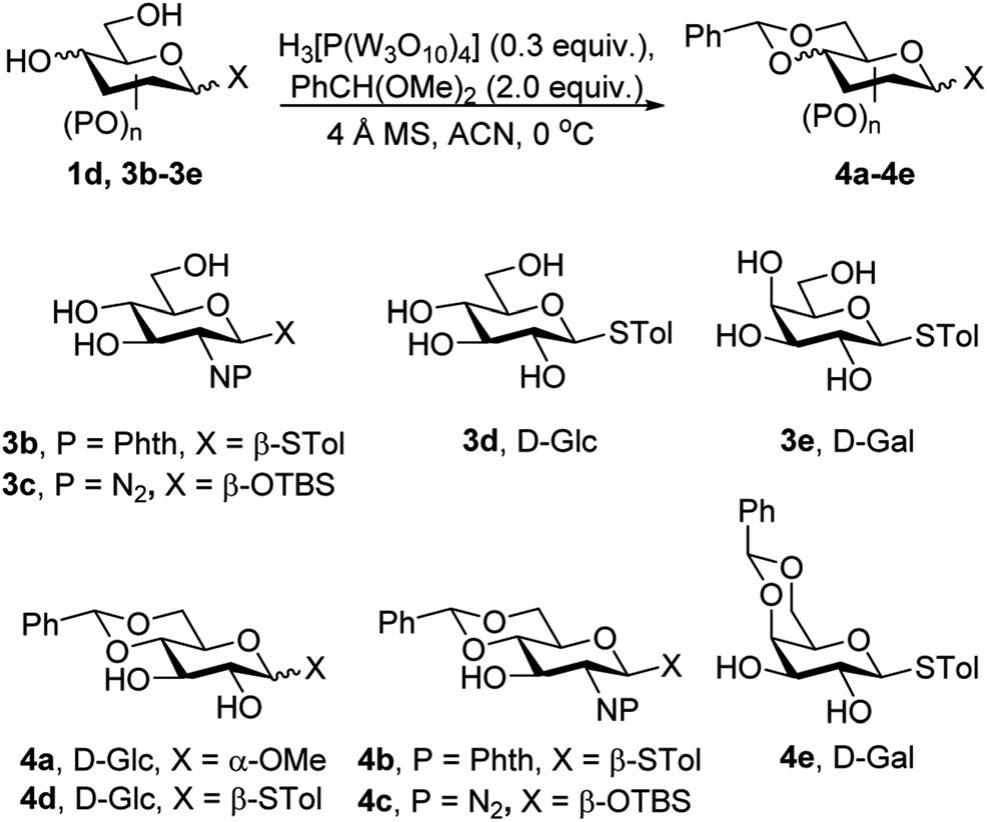				
Entry	SM	H_3_[P(W_3_O_10_)_4_] (equiv.)	Product	Yield
1	1d	0.5	4a	71%
2	1d	0.2	4a	58%
3	1d	0.25	4a	69%
4	1d	0.3^a^	4a	72%
5	1d	0.3^b^	4a	69%
6	3b	0.3	4b	84%
7	3c	0.15	4c	82%
8	3d	0.3	4d	65%
9	3e	0.3	4e	86%

a The benzaldehyde dimethyl acetal was used 2.0 equivalents.

b The benzaldehyde dimethyl acetal was used 3.0 equivalents.

Therefore, the amount of phosphotungstic acid was not reduced any further. When 0.25 equivalent of H_3_[P(W_3_O_10_)_4_] was used with 1d, the yield of product 4a changed the yield remarkably to 69% (Table 2, entry 3). When the amount of catalyst was increased to 0.3 equivalent, the desired product 4a was obtained in a slightly higher yield 72% (Table 2, entry 4). Ultimately, 0.3 equivalent of phosphotungstic acid was determined to be the optimal amount for the regioselective 4,6-*O*-benzylidene acetalization reaction. Increasing the amount of benzaldehyde dimethyl acetal to 3.0 equivalent did not significantly affect the yield of 4a, which remained 69% (Table 2, entry 5). Therefore, various substrates were examined under optimized reaction conditions. β-Thio-d-glucoside derivative 3b was reacted with benzaldehyde dimethyl acetal in presence of phosphotungstic acid. The starting material was consumed in 6 hours and 4b was obtained in 84% yield (Table 2, entry 6). After 3c underwent the reaction, and the desired product 4c was obtained in 82% yield (Table 2, entry 7). 3d and 3e underwent the reaction, and afforded 4d yield in 65% and 4e in 86% yield, respectively (Table 2, entries 8–9). It has produced better yield than PTSA^12*d*^ catalyst and similar yield as CSA. PTA only required less amount than CSA to bring better yield of benzylidene acetal.

### Regioselective reductive ring opening of benzylidene acetals

Following the successful per-*O*-acetylation and regioselective benzylidene ring formation, the *O*-4 selective ring opening reaction was considered. First, methyl 2,3-di-*O*-benzyl-4,6-*O*-benzylidene-α-d-glucopyranoside 5a was used as the optimal substrate. At the outset, 5a, phosphotungstic acid (0.05 equiv.) and triethylsilane (11.0 equiv.) were used in DCM at 0 °C. The expected product 6a was formed and its *O*-4 silylated side product also observed on the TLC plate. To cleave the silyl group at the *O*-4 position, the reaction mixture was treated with tetra-*n*-butyl ammonium fluoride (TBAF, 11.0 equiv.) and acetic acid (AcOH, 11.0 equiv.) at room temperature for 30 minutes, providing 6a in 31% isolated yield (Table 3, entry 1). For further optimization, the amount of H_3_[P(W_3_O_10_)_4_] was increased to 0.1 equivalent, significantly changing the yield of 6a to 80% (Table 3, entry 2). Further increasing the amount of catalyst to 0.2 equivalent did not have a considerable effect in 79% yield (Table 3, entry 3).

**Table 3:** Phosphotungstic acid catalyzed regio-selective *O*-4 ring opening reactions

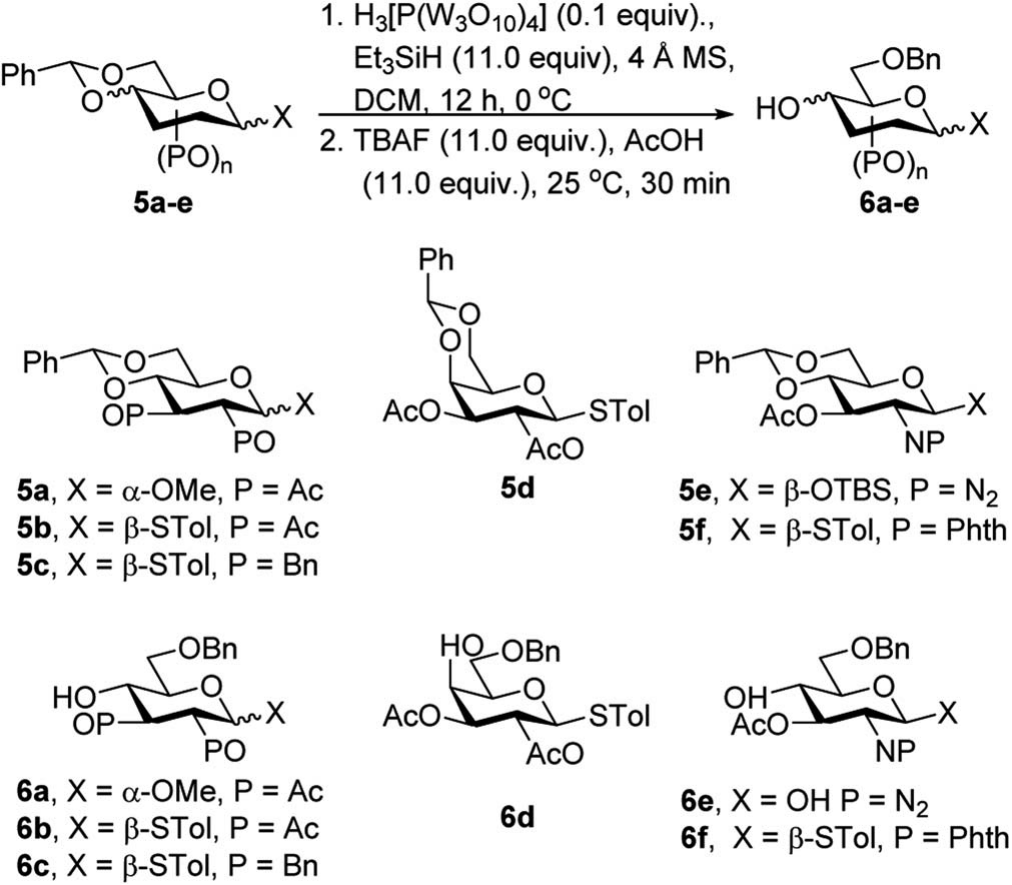				
Entry	SM	H_3_[P(W_3_O_10_)_4_] (equiv.)	Product	Yield
1	5a	0.05	6a	31%
2	5a	0.1	6a	80%
3	5a	0.2	6a	79%
4	5b	0.1	6b	82%
5	5c	0.1	6c	86%
6	5d	0.1	6d	79%
7	5e	0.1	6e	69%
8	5f	0.1	6f	80%

Based on these results, the various substrate for the *O*-4 ring opening reaction was investigated. The β-thio d-glucoside derivatives 5b and 5c underwent the reaction readily, giving 6b in 82% yield and 6c in 86% yield, respectively (Table 3, entries 4–5). The β-thio-d-galactose derivative 5d gave 6d in a similar yield 79% (Table 3, entry 6). Importantly, after the completion of the reaction of 5e, TBAF and AcOH were used to cleave its *O*-4 silylated group. However, under TBAF and AcOH condition, -OTBS was also removed, producing corresponding diol 6e in 69% (*α*/*β* = 3/2) yield (Table 3, entry 7). Finally, 4-methylphenyl 3-*O*-acetyl-4,6-*O*-benzylidene-2-deoxyl-2-phthalimido-1-thio-β-d-glucopyranoside 5f freely underwent the reaction, giving the corresponding product 6f in 80% yield (Table 3, entry 8). The PTA is little higher cost than TFA. However, PTA brought better yield than TFA^12*i*^ catalysed *O*-4 reductive ring-opening reaction.

### Glycosylation reactions

**Fig. 2:**
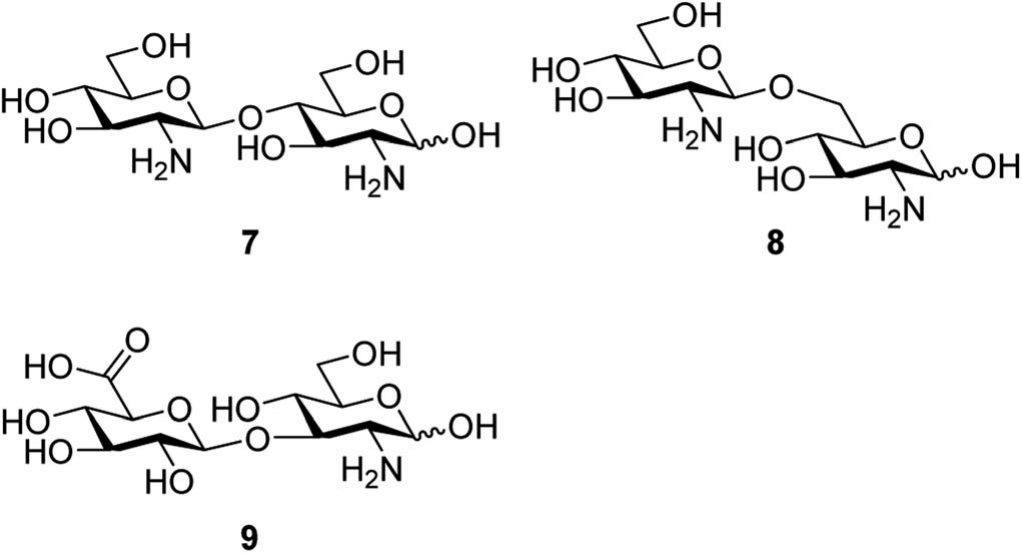
Structures of the disaccharide of chitosan 7, lipid A 8 and hyaluronic acid 9.

Obtaining highly stereo-selective glycosidic linkages is one of the most challenges in carbohydrate synthesis, as it is affected by solvent effect, neighboring group effect, anomeric effect, temperature effect and promoter effect. Use of phosphotungstic acid which is a large catalyst may lead to increased selectivities in glycosylations. Phosphotungstic acid was used in the glycosylation to synthesize biologically important disaccharide molecules. Chitosan plays a crucial role in tissue repair, angiogenesis, tumor growth and drug delivery.^29^ The disaccharide of chitosan contains β-d-GlcNH_2_(1 → 4)-d-GlcNH_2_7. Lipid A is a lipophilic portion of bacterial lipopolysaccharides and has a β-d-GlcNH_2_(1 → 6)-d-GlcNH_2_ backbone 8.^30^ Hyaluronic acid is another important biological molecule,^31^ which is involved in cell-migration, tumor inhibition, adhesion and other processes. It consists of a β-d-GlcA (1 → 3)-d-GlcNH_2_ disaccharide repeating unit 9 (Fig. 2).

During the glycosylation reaction, the acid-sensitive group such as the benzylidene ring was stable and exhibited good tolerance of the phosphotungstic acid catalyst. The catalyst was used as a promoter in the preparation of a disaccharide 13. With donor 5f and acceptor 6g. The reaction proceeded conveniently to provide compound 13 in 82% yield (Table 4, entry 1). The derivatives of lipid A have immune-modulator characteristic. They are therefore used as adjuvants for vaccinations and treatments for many diseases. Two protected disaccharides 14 (83%) and 15 (53%) were synthesized using phosphotungstic acid (Table 4, entries 2–3). Eventually, phosphotungstic acid was used to synthesize 1,3-linked disaccharide with acceptor 4c and protected donors 5f and 10. The expected products 16 and 17 were obtained in good yields (Table 4, entries 4–5). Three biologically important disaccharide backbones were prepared exclusively in β-form with the participation of the neighbouring group. All of the reactions proceeded smoothly in the presence of phosphotungstic acid as the catalyst. The glycosylation proceeded even at room temperature in the presence of PTA while other catalyst such as BF_3_.THF, TMSOTf and Ag(OTf)_2_ required lower temperature.

**Table 4:** Phosphotungstic acid catalyzed glycosylation with various donors and acceptors

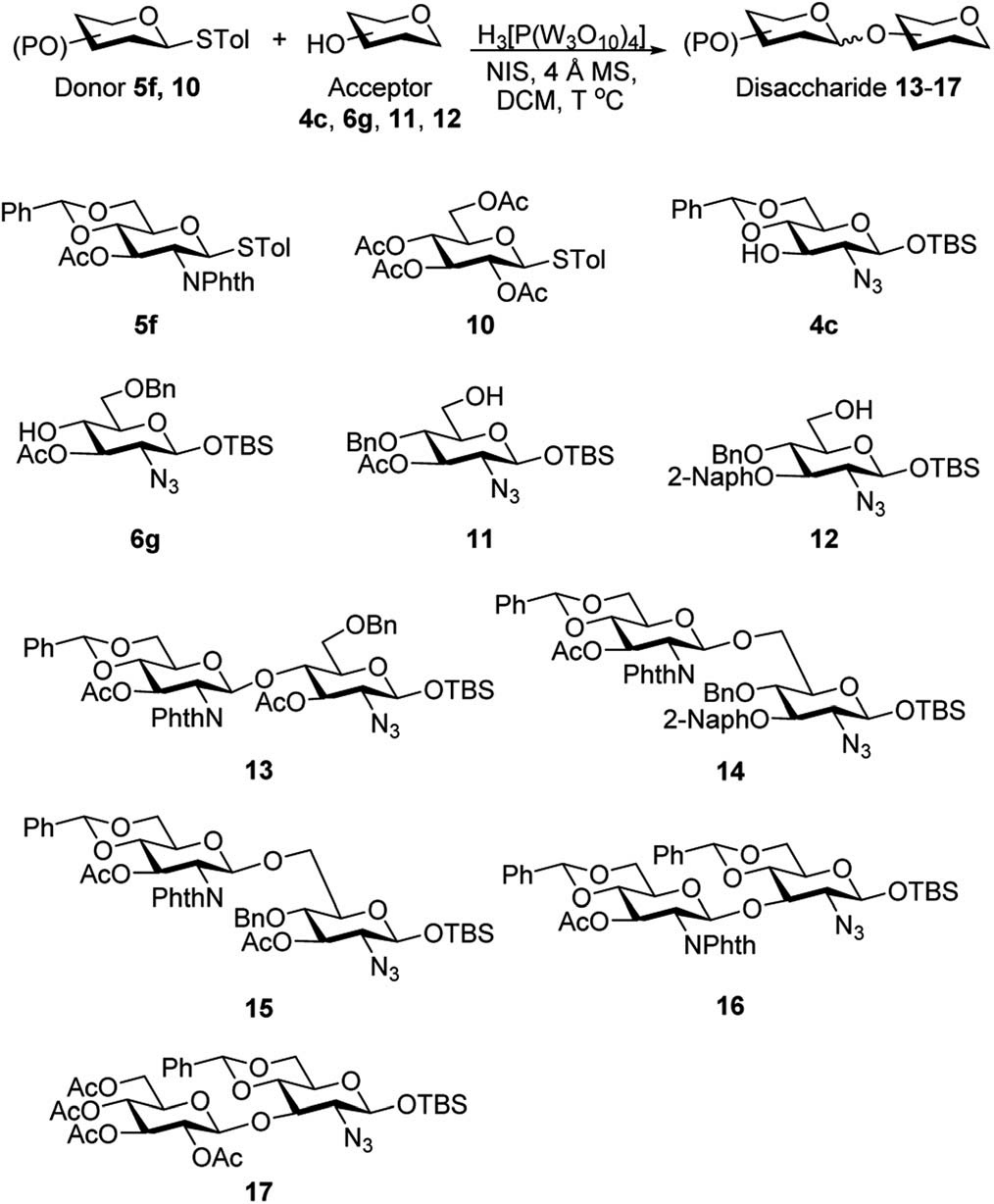					
Entry	Donor	Acceptor	*T* ^°^C	Product (*α*/*β*)	Yield
1	5f	6g	25	13 (*β* only)	82%
2	5f	11	0	14 (*β* only)	83%
3	5f	12	−40	15 (*β* only)	53%
4	5f	4c	25	16 (*β* only)	86%
5	10	4c	25	17 (*β* only)	67%

## Conclusions

We have successfully established that the phosphotungstic acid was a convenient candidate to replace the corresponding homogeneous acids for various carbohydrates reactions. It could effectively catalyze per-*O*-acetylation, 4,6-*O*-benzylidene acetal formation, regioselective *O*-4 ring-opening, and glycosylation. Notably, the glycosylation reactions brought biologically important disaccharide unites like chitosan, hyaluronic acid and lipid A in ambient condition. The different protecting groups such as benzylidene acetal, –OAc, –NPhth, –OBn, 2-Naph, –OTBS and azido were well tolerated during the reaction. It provides moderate to excellent yields under optimal reaction conditions. Phosphotungstic acid is a useful acidic catalyst for various reactions of carbohydrates.

## Experimental section

### General information

The reactions were conducted in flame-dried glassware, under the nitrogen atmosphere. Acetonitrile and dichloromethane were purified and dried from a safe purification system containing activated Al_2_O_3_. All reagents obtained from commercial sources were used without purification unless otherwise mentioned. Flash column chromatography was carried out on Silica Gel 60. TLC was performed on pre-coated glass plates of Silica Gel 60 F254 detection was executed by spraying with a solution of Ce(NH_4_)_2_(NO_3_)_6_ (0.5 g), (NH_4_)_6_Mo_7_O_24_ (24.0 g) and H_2_SO_4_ (28.0 mL) in water (500.0 mL) and subsequent heating on a hot plate. Optical rotations were measured at 589 nm (Na), ^1^H, ^13^C NMR, DEPT, ^1^H–^1^H COSY, ^1^H–^13^C COSY, and NOESY spectra were recorded with 400 MHz instruments. Chemical shifts are in ppm from Me_4_Si generated from the CDCl_3_ lock signal at *δ* 7.26. IR spectra were taken with a FT-IR spectrometer using NaCl plates. Mass spectra were analyzed on orbitrap instrument with an ESI source.

### Acetylation

#### General procedure for per-*O*-acetylation reaction

A round bottom flask equipped with a magnetic stirrer bar was charged with 1a–1f (200 mg for 1a–1e, 500 mg for 1f, 1.0 equiv.) and to this were added phosphotungstic acid (0.02 equiv.) and acetic anhydride (10.0 equiv.) under nitrogen atmosphere. The reaction mixture was stirred at 28 °C for 24 hours. After completion of the reaction, the reaction mixture was diluted with ethyl acetate and extracted with water (50.0 mL × 3). The combined organic layers were dried over anhydrous MgSO_4_, filtered and concentrated. The crude product was purified by column chromatography on silica gel to give the desired product 2a–2f.

#### 1,2,3,4,6-Penta-*O*-acetyl-d-glucopyranose (2a)

Prepared according to the general procedure discussed above: white solid (405 mg, 93%); *R*_f_ 0.53 (EtOAc/Hex = 1/1); mp 109–113 °C; [*α*]^29^_D_ +62.5 (*c* 1.0, DCM); IR (NaCl) *ν* 1752, 1647, 1371, 1221, 1147 cm^−1^; ^1^H NMR (400 MHz, CDCl_3_) *δ* 6.33 (d, *J* = 3.6 Hz, 1H), 5.71 (d, *J* = 8.4 Hz, 0.2H), 5.50–5.45 (m, 1H), 5.25 (t, *J* = 9.2 Hz, 0.2H), 5.16–5.14 (m, 1H), 5.11 (d, *J* = 4.0 Hz, 1H), 5.09 (d, *J* = 4.0 Hz, 0.5H), 4.29–4.28 (m, 0.5H), 4.25 (d, *J* = 4.4 Hz, 1H), 4.14–4.10 (m, 2H), 4.07 (d, *J* = 2.4 Hz, 0.4H), 2.18 (s, 3H), 2.12 (s, 0.6H), 2.10 (s, 3H), 2.09 (s, 0.7H), 2.04 (s, 3H), 2.04 (s, 0.5H), 2.03 (s, 0.5H), 2.03 (s, 3H), 2.02 (s, 3H), 2.01 (s, 0.6H); ^13^C NMR (100 MHz, CDCl_3_) *δ* 170.6, 170.2, 169.6, 169.4, 168.7, 89.0, 69.8, 69.2, 67.9, 61.4, 20.9, 20.7, 20.6, 20.5, 20.4; HRMS (ESI, M + Na^+^) calcd for C_16_H_22_O_11_Na 413.1060, found 413.1050.

#### 1,2,3,4,6-Penta-*O*-acetyl-d-galactopyranose (2b)

Prepared according to the general procedure discussed above: white soild (353 mg, 81%); *R*_f_ 0.50 (EtOAc/Hex = 1/2); mp 70–80 °C; [*α*]^29^_D_ +80.8 (*c* 1.0, DCM); IR (NaCl) *ν* 1751, 1648, 1373 cm^−1^; ^1^H NMR (400 MHz, CDCl_3_) *δ* 6.38 (d, *J* = 1.6 Hz, 1H), 5.69 (d, *J* = 8.4 Hz, 0.2H), 5.50 (d, *J* = 1.2 Hz, 1H), 5.42 (dd, *J* = 3.2, 1.0 Hz, 0.2H), 5.33 (t, *J* = 1.4 Hz, 2H), 5.09–5.05 (m, 0.2H), 4.34 (td, *J* = 6.8, 1.2 Hz, 1H), 4.14 (t, *J* = 7.0 Hz, 0.4H), 4.10 (d, *J* = 3.3 Hz, 1H), 4.09 (d, *J* = 3.4 Hz, 1H), 4.07–4.05 (m, 0.3H), 2.17 (m, 0.6H), 2.16 (s, 3H), 2.16 (s, 3H), 2.12 (s, 0.6H), 2.05 (s, 0.5H), 2.04 (s, 3H), 2.02 (s, 3H), 2.01 (s, 3H), 1.99 (s, 0.5H); ^13^C NMR (100 MHz, CDCl_3_) *δ* 170.0, 169.9, 169.8, 169.6, 168.6, 89.4, 68.5, 67.2, 67.1, 66.2, 61.0, 20.6, 20.5, 20.4, 20.3; HRMS (ESI, M + Na^+^) calcd for C_16_H_22_O_11_Na 413.1060, found 413.1056.

#### 1,​2,​3,​4,​6-​Penta-*​O*-​acetyl-α-d-​mannopyranose (2c)

Prepared according to the general procedure discussed above: colorless oil (380 mg, 88%); *R*_f_ 0.60 (EtOAc/Hex = 1/1); [*α*]^29^_D_ +50.3 (*c* 1.0, DCM); IR (NaCl) *ν* 2106, 1749, 1645, 1371 cm^−1^; ^1^H NMR (400 MHz, CDCl_3_) *δ* 6.08 (s, 1H), 5.34–5.33 (m, 2H), 5.25 (s, 1H), 4.28 (dd, *J* = 12.0, 4.8 Hz, 1H), 4.08 (dd, *J* = 12.6, 1.8 Hz, 2H), 2.17 (s, 3H), 2.16 (s, 3H), 2.09 (s, 3H), 2.05 (s, 3H), 2.00 (s, 3H); ^13^C NMR (100 MHz, CDCl_3_) *δ* 170.3, 169.7, 169.4, 169.3, 167.8, 90.3, 70.3, 68.5, 68.0, 65.2, 61.8, 20.5, 20.4, 20.3, 20.29, 20.26; HRMS (ESI, M + Na^+^) calcd for C_16_H_22_O_11_Na 413.1060, found 413.1054.

#### Methyl 2,​3,​4,​6-​tetra-*​O*-​acetyl-​α-d-​glucopyranoside (2d)

Prepared according to the general procedure discussed above: white soild (330 mg, 88%); *R*_f_ 0.60 (EtOAc/Hex = 1/2); mp 51–53 °C; [*α*]^29^_D_ +46.7 (*c* 1.0, DCM); IR (NaCl) *ν* 2960, 1750, 1648, 1371, 1225 cm^−1^; ^1^H NMR (400 MHz, CDCl_3_) *δ* 5.47 (dd, *J* = 10.1, 9.5 Hz, 1H), 5.07 (dd, *J* = 10.2, 9.4 Hz, 1H), 4.95 (d, *J* = 3.7 Hz, 1H), 4.90 (dd, *J* = 10.2, 3.7 Hz, 1H), 4.26 (dd, *J* = 12.3, 4.6 Hz, 1H), 4.12–4.08 (m, 1H), 3.98 (ddd, *J* = 10.2, 4.5, 2.3 Hz, 1H), 3.41 (s, 3H), 2.10 (s, 3H), 2.08 (s, 3H), 2.03 (s, 3H), 2.01 (s, 3H); ^13^C NMR (100 MHz, CDCl_3_) *δ* 170.4, 169.9, 169.8, 169.4, 96.6, 70.6, 69.9, 68.3, 67.0, 61.7, 55.3, 20.5, 20.46, 20.4; HRMS (ESI, M + Na^+^) calcd for C_15_H_22_O_10_Na 385.1111, found 385.1111.

#### 1,​2,​3,​5-​Tetra-*O*-acetate-d-​xylopyranose (2e)

Prepared according to the general procedure discussed above: colorless oil (424 mg, 99%); *R*_f_ 0.55 (EtOAc/Hex = 1/1); [*α*]^29^_D_ +51.8 (*c* 1.0, DCM); IR (NaCl) *ν* 2924, 1743, 1649, 1370, 1211 cm^−1^; ^1^H NMR (400 MHz, CDCl_3_) *δ* 6.25 (d, *J* = 3.6 Hz, 1H), 5.70 (d, *J* = 6.8 Hz, 0.18H), 5.46 (t, *J* = 9.9 Hz, 1H), 5.36 (t, *J* = 5.0 Hz, 0.33H), 5.30–5.23 (m, 0.28H), 5.19 (t, *J* = 8.4 Hz, 0.26H), 5.06–5.00 (m, 2H), 4.35 (dd, *J* = 12.0, 4.0 Hz, 0.22H), 4.24 (d, *J* = 6.0 Hz, 0.15H), 4.14 (dd, *J* = 12.4, 4.8 Hz, 0.24H), 3.99 (dd, *J* = 12.0, 6.0 Hz, 0.23H), 3.93 (dd, *J* = 11.2, 5.9 Hz, 1H), 3.70 (t, *J* = 11.0 Hz, 1H), 3.52 (dd, *J* = 12.0, 8.4 Hz, 0.2H). 2.17 (s, 3H), 2.13–2.05 (m, 9H), 2.05 (s, 3H), 2.04 (s, 3H), 2.02 (s, 3H); ^13^C NMR (100 MHz, CDCl_3_) *δ* 170.0, 169.7, 169.6, 168.9, 89.1, 69.2, 69.16, 68.5, 60.5, 20.8, 20.6, 20.6, 20.4; HRMS (ESI, M + Na^+^) calcd for C_13_H_18_O_9_Na 341.0849, found 341.0842.

#### 1,​2,​3,​6-​Tetra-*O*-acetate-4-​*O*-​(2,​3,​4,​6-​tetra-*​O*-​acetyl-​β-​d-​galactopyranosyl)​-​d-​glucopyranose (2f)

Prepared according to the general procedure discussed above: white solid (950 mg, 96%); *R*_f_ 0.44 (EtOAc/Hex = 3/2); mp 85–87 °C; [*α*]^22^_D_ +55.8 (*c* 1.0, DCM); IR (NaCl) *ν* 2981, 2943, 1753, 1649, 1434, 1371 cm^−1^; ^1^H NMR (400 MHz, CDCl_3_) *δ* 6.25 (d, *J* = 3.6 Hz, 1H), 5.67 (d, *J* = 8.4 Hz, 0.2H), 5.46 (t, *J* = 9.6 Hz, 1.2H), 5.35 (d, *J* = 3.6 Hz, 1.2H), 5.27–5.21 (m, 0.3H), 5.12 (dd, *J* = 10.4, 7.6 Hz, 1H), 5.09–5.03 (m, 0.4H), 5.00 (dd, *J* = 10.3, 3.6 Hz, 1H), 4.96 (dd, *J* = 10.2, 3.4 Hz, 1H), 4.48 (d, *J* = 8.0 Hz, 1H), 4.43 (s, 1H), 4.18–4.05 (m, 4H), 4.00 (ddd, *J* = 10.0, 3.6, 1.6 Hz, 1H), 3.88 (t, *J* = 6.8 Hz, 1H), 3.81 (t, *J* = 9.7 Hz, 1H), 2.18 (s, 3H), 2.16 (s, 2H), 2.15 (s, 1H), 2.13 (s, 3H), 2.12 (s, 1H), 2.09 (s, 1H), 2.06 (s, 2H), 2.06 (s, 2H), 2.05 (s, 3H), 2.05 (s, 1H), 2.04 (s, 1H), 2.03 (s, 1H), 2.01 (s, 2H), 1.97 (s, 3H); ^13^C NMR (100 MHz, CDCl_3_) *δ* 170.28, 170.24, 170.08, 170.01, 169.87, 169.56, 169.07, 168.88, 101.13, 88.87, 75.72, 70.91, 70.61, 69.50, 69.31, 69.01, 66.49, 61.36, 60.70, 20.91, 20.80, 20.61, 20.46; HRMS (ESI, M + Na^+^) calcd for C_28_H_38_O_19_Na 701.1905, found 701.1902.

### Acetalization

#### General procedure for benzylidene formation

A solution of 1d, 3b–3e (150 mg for 1d, 3b, 3d, and 3e, 140 mg for 3c, and 120 mg for 3e, 1.0 equiv.) and activated 4 Å MS in anhydrous acetonitrile (2 mL) was stirred for 30 minutes under nitrogen atmosphere at rt. Then the flask was placed into an ice bath. After 3 minutes, benzaldehyde dimethyl acetal (2.0 equiv.) and dried phosphotungstic acid (0.3 equiv. for 1d, 3b, 3d, and 3e and 0.15 equiv. for 3c) were added to the reaction mixture at 0 °C. After completion of the reaction, the reaction mixture was passed through the Celite and extracted with water (25 mL) and ethyl acetate (50 mL × 2). The combined organic layers were washed with brine, dried over anhydrous MgSO_4_, filtered and concentrated under reduced pressure. The crude product was purified by column chromatography on silica gel to afford 4a–4e.

#### Methyl 4,6-*O*-benzylidene-α-d-glucopyranoside (4a)

Prepared according to the general procedure discussed above: white solid. (157 mg, 72%); *R*_f_ 0.35 (EtOAc); mp 161–162 °C; [*α*]^25^_D_ +117.0 (*c* 1.0, DCM); IR (NaCl) *v* 3380, 2915, 2870, 1640, 1453, 1373, 1192, 1145, 1125, 1076, 1030, 1000 cm^−1^; ^1^H NMR (400 MHz, CDCl_3_) *δ* 7.48–7.45 (m, 2H), 7.37–7.32 (m, 3H), 5.49 (s, 1H), 4.73 (d, *J* = 4.0 Hz, 1H), 4.26 (dd, *J* = 9.6, 4.4 Hz, 1H), 3.88 (t, *J* = 9.2 Hz, 1H), 3.80–3.67 (m, 2H), 3.58 (bs, 1H), 3.44 (t, *J* = 12.0 Hz, 1H), 3.41 (s, 3H), 3.29 (bs, 1H), 2.65 (bs, 1H); ^13^C NMR (100 MHz, CDCl3) *δ* 137.2, 129.5, 128.6, 126.5, 102.1, 100.0, 81.1, 73.0, 71.8, 69.1, 62.8, 55.8; HRMS (ESI, M + Na^+^) calcd for C_14_H_18_O_6_Na 305.1001, found 305.1005.

#### 4-Methylphenyl 4,6-*O*-benzylidene-2-deoxyl-1-thio-2-phthalimido-β-d-glucopyranoside (4b)

Prepared according to the general procedure discussed above: white solid (154 mg, 84%); *R*_f_ 0.46 (EtOAc/Hex = 1/2); mp 121–122 °C; [*α*]^25^_D_ +40.0 (*c* 0.45, DCM); IR (NaCl) *v* 3443, 2868, 2090, 1775, 1644 cm^−1^; ^1^H NMR (400 MHz, CDCl_3_) *δ* 7.91–7.78 (m, 2H), 7.76–7.68 (m, 2H), 7.49–4.41 (m, 2H), 7.38–7.31 (m, 3H), 7.27–7.24 (m, 2H), 7.05 (d, *J* = 7.6 Hz, 2H), 5.60 (d, *J* = 10.8 Hz, 1H), 5.53 (s, 1H), 4.58 (t, *J* = 8.8 Hz, 1H), 4.36 (dd, *J* = 10.4, 4.8 Hz, 1H), 4.27 (t, *J* = 10.0 Hz, 1H), 3.79 (t, *J* = 10.0 Hz, 1H), 3.68–3.60 (m, 1H), 3.55 (t, *J* = 9.2 Hz, 1H), 2.75 (s, 1H), 2.29 (s, 3H); ^13^C NMR (100 MHz, CDCl_3_) *δ* 165.7, 165.0, 135.9, 134.3, 131.6, 130.7, 129.1, 127.1, 126.8, 125.8, 125.2, 123.7, 121.3, 120.8, 99.3, 81.9, 79.3, 67.7, 67.1, 66.0, 53.0, 18.6; HRMS (ESI, M + Na^+^) calcd for C_28_H_25_NO_6_SNa 526.1301, found 526.1299.

#### 2-Azido-4,6-*O*-benzylidene-2-deoxyl-1-*O-tert*-butyldi-methylsilyl-β-d-glucopyranoside (4c)

Prepared according to the general procedure discussed above: colorless oil. (106 mg, 82%); *R*_f_ 0.59 (EtOAc/Hex = 1/2); [*α*]^25^_D_ −19.9 (*c* 0.05, DCM); IR (NaCl) *v* 3443, 3039, 2860, 2111 cm^−1^; ^1^H NMR (400 MHz, CDCl_3_) *δ* 7.52–7.47 (m, 2H), 7.41–7.35 (m, 3H), 5.50 (s, 1H), 4.59 (d, *J* = 8.0 Hz, 1H), 4.26 (t, *J* = 4.0 Hz, 1H), 3.74 (t, *J* = 10.0 Hz, 1H), 3.59–3.46 (m, 2H), 3.31–3.25 (m, 2H), 3.18 (br, 1H), 0.97 (s, 9H), 0.18 (s, 6H); ^13^C NMR (100 MHz, CDCl_3_) *δ* 136.8, 129.2, 128.3, 126.3, 101.8, 97.4, 80.7, 71.5, 68.9, 68.4, 66.2, 25.5, 17.8, −4.4, −5.3; HRMS (ESI, M + Na^+^) calcd for C_19_H_29_N_3_O_5_SiNa 430.1774, found 430.1770.

#### 4-Methylphenyl 4,6-*O*-benzylidene-1-thio-β-d-glucopyranoside (4d)

Prepared according to the general procedure discussed above: white solid. (126 mg, 65%); *R*_f_ 0.62 (EtOAc); mp 177–178 °C; [*α*]^25^_D_ −161.30 (*c* 1.0, DCM); IR (NaCl) *v* 3442, 2870, 2088, 1643, 1493, 1454, 1381, 1265, 1214, 1165, 1085, 1030, 1005, 974 cm^−1^; ^1^H NMR (400 MHz, CDCl_3_) *δ* 7.48–7.42 (m, 4H), 7.38–7.35 (m, 3H), 7.15 (d, *J* = 8.0 Hz, 2H), 5.52 (s, 1H), 4.57 (d, *J* = 9.6 Hz, 1H), 4.39 (dd, *J* = 10.4, 4.4 Hz, 1H), 3.85 (t, *J* = 8.4 Hz, 1H), 3.78 (t, *J* = 7.4 Hz, 1H), 3.52–3.47 (m, 2H), 3.43 (t, *J* = 9.0 Hz, 1H), 2.80 (bs, 1H), 2.65 (bs, 1H), 2.36 (s, 3H); ^13^C NMR (100 MHz, CDCl_3_) *δ* 138.6, 136.8, 133.4, 129.7, 129.2, 128.2, 127.3, 126.2, 101.7, 88.5, 80.0, 74.3, 72.3, 70.3, 68.4, 21.1; HRMS (ESI, M + Na^+^) calcd for C_20_H_22_O_5_SNa 397.1086, found 397.1084.

#### 4-Methylphenyl 4,6-*O*-benzylidene-1-thio-β-d-galactopyranoside (4e)

Prepared according to the general procedure discussed above: white solid (135 mg, 86%); *R*_f_ 0.34 (EtOAc); mp 145–146 °C; [*α*]^25^_D_ −175.30 (*c* 1.0, DCM); IR (NaCl) *v* 3440, 2920, 2665, 2248, 1644, 1493, 1451, 1402, 1361, 1276, 1245, 1197, 1165, 1101, 1070, 1042, 993, 970 cm^−1^; ^1^H NMR (400 MHz, CDCl_3_) *δ* 7.45 (d, *J* = 8.0 Hz, 2H), 7.25 (d, *J* = 4.0 Hz, 5H), 6.99 (d, *J* = 8.0 Hz, 2H), 5.37 (s, 1H), 4.32 (d, *J* = 8.8 Hz, 1H), 4.25 (d, *J* = 12.4 Hz, 1H), 4.06 (d, *J* = 3.2 Hz, 1H), 3.89 (d, *J* = 12.4 Hz, 1H), 3.55 (bs, 1H), 3.50 (t, *J* = 9.2 Hz, 1H), 3.40 (s, 1H), 2.54 (bs, 2H), 2.24 (s, 3H); ^13^C NMR (100 MHz, CDCl_3_) *δ* 138.5, 137.5, 134.2, 129.6, 129.2, 128.1, 126.5, 101.3, 86.9, 75.2, 73.7, 69.9, 69.2, 68.6, 21.2; HRMS (ESI, M + Na^+^) calcd for C_20_H_22_O_5_SNa 397.1086, found 397.1086.

### Regioselective reductive ring opening of benzylidene acetals

#### General procedure for regio-selective *O*-4 ring opening reaction

A solution of 5a–5f (100 mg for 5a–5e and 200 mg for 5f, 1.0 equiv.) and activated 4 Å MS (100 mg) in anhydrous dichloromethane (1.0 mL) was stirred under nitrogen atmosphere at room temperature. Then the flask was placed into an ice bath for 5 minutes and triethylsilane (11 equiv.), phosphotungstic acid (0.1 equiv.) were added to the reaction mixture at 0 °C. Then the reaction was allowed to stir at same temperature for 12 hours. After completion of the reaction, were added 1 M tetra-*n*-butyl ammonium fluoride (11 equiv.) and acetic acid (11 equiv.) at 0 °C, and stirred for 30 minutes at 25 °C. The molecular sieves were removed by filtration through Celite. The filtrate was extracted with EtOAc (20 mL × 3) and water (20 mL). The combined organic layers were dried over anhydrous MgSO_4_, filtered and concentrated. It was purified by column chromatography on silica gel to give the desired product 6a–6f.

#### Methyl 2,3-di-*O*-acetyl-6-*O*-benzyl-α-d-glucopyranoside (6a)

Prepared according to the general procedure discussed above: yellow liquid (88 mg, 80%); *R*_f_ 0.35 (EtOAc/Hex = 1/1); [*α*]^26^_D_ +109.5 (*c* 1.0, DCM); IR (NaCl) *ν* 3474, 2919, 2871, 1747, 1452, 1371 cm^−1^; ^1^H NMR (400 MHz, CDCl_3_) *δ* 7.37–7.29 (m, 5H), 5.30 (t, *J* = 10.0 Hz 1H), 4.91 (d, *J* = 3.6 Hz, 1H), 4.87 (dd, *J* = 10.1, 3.6 Hz, 1H), 4.60 (q, *J* = 12.0 Hz, 2H), 3.80–3.70 (m, 4H), 3.40 (s, 3H), 2.83 (s, 1H), 2.09 (s, 3H), 2.08 (s, 3H).; ^13^C NMR (100 MHz, CDCl_3_) *δ* 176.8, 171.3, 170.2, 137.7, 128.3, 127.6, 127.5, 96.6, 73.5, 72.9, 70.7, 70.1, 70.0, 69.2, 55.1, 20.8, 20.6; HRMS (ESI, M + Na^+^) calcd for C_18_H_24_O_8_Na 391.1369, found 391.1362.

#### 4-Methylphenyl 2,3-di-*O*-acetyl-6-​*O*-benzyl-1-thio-β-d-glucopyranoside (6b)

Prepared according to the general procedure discussed above: yellow oil (83 mg, 82%); *R*_f_ 0.25 (EtOAc/Hex = 1/2); [*α*]^26^_D_ −37.4 (*c* 0.8, DCM); IR (NaCl) *ν* 3478, 3030, 2920, 1752, 1494, 1373 cm^−1^; ^1^H NMR (400 MHz, CDCl_3_) *δ* 7.40–7.28 (m, 7H), 7.08 (d, *J* = 8.4 Hz, 2H), 5.06 (t, *J* = 9.6 Hz, 1H), 4.90 (dd, *J* = 9.6, 9.2 Hz, 1H), 4.63 (d, *J* = 10.0 Hz, 1H), 4.57 (d, *J* = 5.2 Hz, 2H), 3.80 (dd, *J* = 6.4, 4.8 Hz, 2H), 3.72 (t, *J* = 9.4 Hz, 1H), 3.58–3.52 (m, 1H), 2.32 (s, 3H), 2.09 (s, 3H), 2.07 (s, 3H).; ^13^C NMR (100 MHz, CDCl_3_) *δ* 171.2, 169.5, 138.4, 137.6, 133.4, 129.6, 128.4, 128.0, 127.8, 127.6, 85.8, 78.4, 76.7, 73.7, 70.0, 69.9, 69.87, 21.1, 20.8.; HRMS (ESI, M^+^Na^+^) calcd for C_24_H_28_O_7_SNa 483.1453, found 483.1446.

#### 4-​Methylphenyl 2,​3,​6-​tri-​*O*-benzyl​-​1-​thio-​β-d-​glucopyranoside (6c)

Prepared according to the general procedure discussed above: yellow oil (86 mg, 86%); *R*_f_ 0.25 (EtOAc/Hex = 1/2); mp 62–64 °C; [*α*]^26^_D_ −7.4 (*c* 0.8, DCM); IR (NaCl) *ν* 3478, 3030, 2920, 1752, 1494, 1373 cm^−1^; ^1^H NMR (400 MHz, CDCl_3_) *δ* 7.46 (d, *J* = 8.1 Hz, 2H), 7.44–7.40 (m, 2H), 7.38–7.27 (m, 13H), 7.05 (d, *J* = 7.9 Hz, 2H), 4.93 (d, *J* = 4.4 Hz, 1H), 4.90 (d, *J* = 6.0 Hz, 1H), 4.76 (dd, *J* = 16.0, 15.2 Hz, 2H), 4.63 (d, *J* = 9.6 Hz, 1H), 4.57 (d, *J* = 4.4 Hz, 2H), 3.77 (t, *J* = 5.0 Hz, 2H), 3.64 (t, *J* = 9.2 Hz, 1H), 3.53 (t, *J* = 8.6 Hz, 1H), 3.49–3.42 (m, 2H), 2.55 (s, 1H), 2.31 (s, 3H).; ^13^C NMR (100 MHz, CDCl_3_) *δ* 138.4, 138.2, 138.1, 138.0, 137.9, 137.7, 132.6, 129.7, 129.6, 128.6, 128.4, 128.37, 128.2, 127.9, 127.7, 87.9, 86.1, 80.4, 78.0, 75.5, 75.3, 73.6, 71.6, 70.3, 21.1; HRMS (ESI, M + Na^+^) calcd for C_34_H_36_O_5_SNa 579.2181, found 579.2184.

#### 4-Methylphenyl 2,3-di-*O*-acetyl-6-​*O*-benzyl-1-thio-β-d-galacopyranoside (6d)

Prepared according to the general procedure discussed above: white solid (80 mg, 79%); *R*_f_ 0.50 (EtOAc/Hex = 1/1); mp 114–115 °C; [*α*]^26^_D_ +1.8 (*c* 0.8, DCM); IR (NaCl) *ν* 3478, 3030, 2922, 1750, 1494, 1369 cm^−1^; ^1^H NMR (400 MHz, CDCl_3_) *δ* 7.41 (d, *J* = 8.0 Hz, 2H), 7.36–7.29 (m, 5H), 7.07 (d, *J* = 8.1 Hz, 2H), 5.27 (t, *J* = 9.8 Hz, 1H), 4.96 (dd, *J* = 9.6, 3.0 Hz, 1H), 4.63 (d, *J* = 10.0 Hz, 1H), 4.56 (d, *J* = 4.8 Hz, 2H), 4.17 (t, *J* = 3.2 Hz, 1H), 3.78 (t, *J* = 4.4 Hz, 2H), 3.71 (t, *J* = 5.0 Hz, 1H), 2.64–2.59 (m, 1H), 2.31 (s, 3H), 2.08 (s, 3H), 2.07 (s, 3H); ^13^C NMR (100 MHz, CDCl_3_) *δ* 170.2, 169.5, 164.8, 155.2, 138.3, 137.5, 133.3, 129.6, 128.5, 128.3, 127.9, 127.8, 86.5, 76.7, 74.5, 73.8, 69.5, 68.3, 67.6, 29.7, 21.2, 20.9; HRMS (ESI, M + Na^+^) calcd for C_24_H_28_O_7_SNa 483.1453, found 483.1458.

#### 3-*O*-Acetyl-2-azido-6-*​O*-benzyl-2-deoxyl-d-glucopyranoside (6e)

Prepared according to the general procedure discussed above: colorless liquid (52 mg, 69%), *α*/*β* = 3/2; *R*_f_ 0.30 (EtOAc/Hex = 1/1); [*α*]^25^_D_ −5.5 (*c* 0.6, DCM); IR (NaCl) *ν* 3417, 2923, 2871, 2110, 1720, 1454, 1364 cm^−1^.; ^1^H NMR (400 MHz, CDCl_3_) *δ* 7.37–7.27 (m, 8.4H), 5.33 (d, *J* = 10.0 Hz, 0.7H), 5.30 (d, *J* = 3.2 Hz, 1H), 4.78 (dd, *J* = 10.2, 8.7 Hz, 0.9H), 4.58–4.54 (m, 3H), 4.08 (ddd, *J* = 9.2, 5.7, 2.8 Hz, 1H), 3.74 (t, *J* = 3.1 Hz, 0.8H), 3.72 (d, *J* = 2.8 Hz, 0.6H), 3.68–3.62 (m, 2H), 3.55 (d, *J* = 9.5 Hz, 1H), 3.52–3.49 (m, 1H), 3.34 (dd, *J* = 10.2, 8.0 Hz, 0.8H), 3.28 (dd, *J* = 10.5, 3.4 Hz, 1H), 2.16 (s, 3H), 2.15 (s, 2H); ^13^C NMR (100 MHz, CDCl_3_) *δ* 172.0, 171.8, 137.3, 137.2, 128.53, 128.5, 128.02, 127.98, 95.9, 91.9, 75.7, 74.6, 73.7, 73.6, 73.5, 70.5, 70.3, 70.1, 69.5, 69.4, 64.5, 61.4, 21.0; HRMS (ESI, M + Na^+^) calcd for C_15_H_19_O_6_N_3_Na 360.1172, found 360.1164.

#### 4-Methylphenyl 3-*O*-acetyl-6-​*O*-benzyl-2-deoxyl-2-phthalimi-do-1-thio-β-d-glucopyranoside (6f)

Prepared according to the general procedure discussed above: white solid (159 mg, 80%); *R*_f_ 0.38 (EtOAc/Hex = 1/1); mp 54–56 °C; [*α*]^23^_D_ +17.0 (*c* 1.0, DCM); IR (NaCl) *ν* 3478, 3030, 2922, 2855, 1750, 1494, 1430, 1369 cm^−1^; ^1^H NMR (400 MHz, CDCl_3_) *δ* 7.89–7.84 (m, 2H), 7.77–7.72 (m, 2H), 7.40–7.31 (m, 5H), 7.29 (d, *J* = 8.2 Hz, 2H), 7.03 (d, *J* = 7.9 Hz, 2H), 5.68–5.61 (m, 2H), 4.60 (q, *J* = 11.8 Hz, 2H), 4.26 (t, *J* = 10.4 Hz, 1H), 3.88–3.72 (m, 4H), 2.91 (s, 1H), 2.29 (s, 3H), 1.91 (s, 3H); ^13^C NMR (100 MHz, CDCl_3_) *δ* 171.1, 167.8, 167.3, 138.4, 137.7, 134.3, 134.1, 133.5, 131.6, 131.2, 129.6, 128.4, 127.8, 127.7, 127.5, 123.6, 123.5, 83.2, 78.3, 74.3, 73.7, 71.0, 70.2, 53.6, 52.6, 21.1, 20.7, 20.5, 13.9; HRMS (ESI, M + Na^+^) calcd for C_30_H_29_NO_7_SNa 570.1562, found 570.1561.

### Glycosylation reactions

#### 2-Azido-2-deoxy-3-*O*-acetyl-6-*O*-benzyl-1-*O-tert*-butyldime-thylsilyl-4-*O*-(3-*O*-ace-tyl-4,6-*O*-benzylidene-2-deoxy-2-phthalimido-β-d-glucopyranosyl)-β-d-glucopyranoside (13)

A solution of acceptor 6g (50 mg, 0.11 mmol), donor 5f (120 mg, 0.22 mmol), and activated 4 Å molecular sieves (300 mg) in dichloromethane (1 mL) was stirred for 30 minutes at room temperature. After *N*-iodosuccinimide (148 mg, 0.66 mmol) and dried phosphotungstic acid (158 mg, 0.055 mmol) were added, the reaction mixture was stirred for 5 hours. When the reaction was completed, molecular sieves were removed by filtration through Celite. The filtrate was extracted with aqueous sodium thiosulfate (10 mL) and brine (10 mL), and the organic layer was dried over anhydrous MgSO_4_, filtered, and concentrated under vacuum. The residue was purified by column chromatography on silica gel to give the desired product 13 (79 mg, 82%) as a white soild. *R*_f_ 0.47 (EtOAc/Hex = 1/2); mp 112–114 °C; [*α*]^24^_D_ −13.4 (*c* 1.0, DCM); IR (NaCl) *ν* 3479, 2931, 2112, 1751, 1720, 1386, 1227, 1105, 1082 cm^−1^; ^1^H NMR (400 MHz, CDCl_3_) *δ* 7.83 (dd, *J* = 5.5, 3.1 Hz, 2H), 7.71 (dd, *J* = 5.5, 3.0 Hz, 2H), 7.46 (d, *J* = 4.4 Hz, 1H), 7.44 (d, *J* = 2.1 Hz, 1H), 7.38–7.35 (m, 3H), 7.32–7.27 (m, 3H), 7.21–7.19 (m, 2H), 5.84 (dd, *J* = 10.2, 9.4 Hz, 1H), 5.52 (s, 1H), 5.50 (d, *J* = 8.4 Hz, 1H), 4.90 (dd, *J* = 10.4, 9.2 Hz, 1H), 4.47 (d, *J* = 8.0 Hz, 1H), 4.39–4.36 (m, 2H), 4.26 (d, *J* = 12.0 Hz, 1H), 4.21 (dd, *J* = 10.4, 8.4 Hz, 1H), 3.93 (t, *J* = 9.6 Hz, 1H), 3.79–3.70 (m, 2H), 3.65–3.59 (m, 1H), 3.36–3.33 (m, 1H), 3.30–3.25 (m, 3H), 2.15 (s, 3H), 1.86 (s, 3H), 0.88 (s, 9H), 0.07 (s, 3H), 0.07 (s, 3H); ^13^C NMR (101 MHz, CDCl_3_) *δ* 170.1, 169.2, 137.8, 136.6, 134.2, 131.2, 129.1, 128.1, 127.4, 127.3, 126.1, 123.4, 101.5, 98.2, 96.7, 78.8, 74.6, 74.2, 72.7, 72.4, 69.5, 68.5, 67.3, 66.3, 65.8, 55.5, 25.4, 21.1, 20.4, 17.8, −4.6, −5.4; HRMS (ESI, M + Na^+^) calcd for C_44_H_52_N_4_O_13_SiNa 895.31978, found 895.3181.

#### 2-Azido-4-*O*-benzyl-1-*O-tert*-butyldimethylsilyl-2-deo-xy-3-*O*-(2-naphthylmethyl)-6-*O*-(3-*O*-acetyl-4,6-*O*-be-nzylidene-2-deoxy-2-phthalimi-do-β-d-glucopyranosyl)-β-d-glucopyranoside (14)

To a solution of acceptor 12 (241 mg, 0.44 mmol), donor 5f (200 mg, 0.37 mmol), *N*-iodosuccinimide (90 mg, 0.40 mmol) and activated 4 Å molecular sieves (150 mg) in dichloromethane (3.0 mL) were stirred for 1 hour at room temperature. After phosphotungstic acid (126 mg, 0.044 mmol) was added at 0 °C, the reaction mixture was stirred for 12 hours at same temperature. When the reaction was completed, the mixture was quenched by triethylamine and molecular sieves were removed by filtration through Celite. The filtrate was extracted with aqueous sodium thiosulfate (10 mL) and brine (10 mL), and the organic layer was dried over anhydrous MgSO_4_, filtered, and concentrated under vacuum. The residue was purified by column chromatography on silica gel to give the desired product 14 (84 mg, 53%) as pali yellow soild. *R*_f_ 0.50 (EtOAc/Hex = 1/2); mp 90–93 °C; [*α*]^25^_D_ −93.63 (*c* 0.1, DCM); IR (NaCl) *v* 3414, 2106, 1748, 1718, 1645 cm^−1^; ^1^H NMR (400 MHz, CDCl_3_) *δ* 7.86–7.79 (m, 3H), 7.75–7.72 (m, 3H), 7.60–7.58 (m, 2H), 7.51–7.45 (m, 5H), 7.39 (d, *J* = 4.4 Hz, 3H), 7.28–7.24 (m, 4H), 7.03 (d, *J* = 6.0 Hz, 2H), 5.87 (t, *J* = 10 Hz, 1H), 5.57 (s, 1H), 5.53 (d, *J* = 8.4, 1H), 5.00 (d, *J* = 11.2 Hz, 1H), 4.86 (d, *J* = 11.2 Hz, 1H), 4.60 (d, *J* = 10.8 Hz, 1H), 4.39 (t, *J* = 8, 2H), 4.32 (d, *J* = 10.8 Hz, 1H), 4.23 (d, *J* = 10 Hz, 1H), 3.88–3.79 (m, 2H), 3.77–3.69 (m, 2H), 3.46–3.36 (m, 2H), 3.34–3.29 (m, 2H), 1.90 (s, 3H), 0.89 (s, 9H), 0.08 (s, 3H), 0.00 (s, 3H); ^13^C NMR (100 MHz, CDCl_3_) *δ* 170.0, 137.4, 136.8, 135.3, 134.0, 133.1, 132.8, 129.0, 128.2, 128.1, 128.0, 127.8, 127.6, 127.5, 127.5, 126.5, 126.1, 125.9, 125.9, 125.8, 123.3, 101.5, 98.0, 97.0, 82.4, 79.0, 77.5, 75.2, 74.7, 74.1, 69.8, 68.5, 68.3, 67.9, 66.1, 55.0, 30.7, 25.4, 20.4, 17.7, −4.4, −5.6; HRMS (ESI, M + Na^+^) calcd for C_53_H_58_N_4_O_12_SiNa 993.3718, found 993.3707.

#### 2-Azido-2-deoxy-3-*O*-acetyl-4-*O*-benzyl-1-*O-tert*-butyldime-thylsilyl-6-*O*-(3-*O*-ace-tyl-4,6-*O*-benzylidene-2-phthalimido-2-deoxy-β-d-glucopyranosyl)-β-d-*gluco*-pyranoside (15)

To a solution of acceptor 11 (99 mg, 0.22 mmol), donor 5f (100 mg, 0.18 mmol), *N*-iodosuccinimide (61 mg, 0.27 mmol) and activated 4 Å molecular sieves (200 mg) in dichloromethane (1.5 mL) were stirred for 1 hour at room temperature. After dried phosphotungstic acid (158 mg, 0.054 mmol) was added, the reaction mixture was stirred for 3 hours at −40 °C. When the reaction was completed, it was quenched by triethylamine and molecular sieves were removed by filtration through Celite. The filtrate was extracted with aqueous sodium thiosulfate (10 mL) and brine (10 mL), and the organic layer was dried over anhydrous MgSO_4_, filtered, and concentrated under vacuum. The residue was purified by column chromatography on silica gel to give the desired product 15 (84 mg, 53%). *R*_f_ 0.30 (EtOAc/Hex = 1.5/8.5); mp 175–176 °C; [*α*]^25^_D_ −152.2 (*c* 1.0, DCM); IR (NaCl) *v* 2951, 2931, 2884, 2858, 2111, 1747, 1718, 1645, 1469, 1387, 1312, 1225, 1176, 1104, 1042, 1014, 1001, 970, 917, 872, 840, 784, 738, 700 cm^−1^; ^1^H NMR (400 MHz, CDCl_3_) *δ* 7.76 (s, 2H), 7.65–7.63 (m, 2H), 7.47–7.44 (m, 2H), 7.37–7.35 (m, 3H), 7.22–7.21 (m, 3H), 6.96–6.94 (m, 2H), 5.84 (t, *J* = 9.2 Hz, 1H), 5.54 (s, 1H), 5.51 (d, *J* = 8.4 Hz, 1H), 4.91 (t, *J* = 8.8 Hz, 1H), 4.54 (d, *J* = 9.2 Hz, 1H), 4.41–4.35 (m, 2H), 4.27 (d, *J* = 4 Hz, 2H), 4.00 (d, *J* = 10.4 Hz, 1H), 3.86–3.76 (m, 2H), 3.75–3.69 (m, 2H), 3.48–3.37 (m, 2H), 3.22 (dd, *J* = 12.8, 8.8 Hz, 1H), 1.97 (s, 3H), 1.89 (s, 3H), 0.86 (S, 9H), 0.07 (s, 3H), 0.01 (s, 3H); ^13^C NMR (100 MHz, CDCl_3_) *δ* 170.1, 169.7, 137.1, 136.8, 134.2, 129.1, 128.3, 128.2, 127.8, 127.5, 126.2, 123.5, 101.6, 98.1, 97.0, 79.1, 76.0, 74.3, 74.0, 73.5, 69.8, 68.6, 67.8, 66.3, 66.2, 55.1, 25.4, 20.8, 20.5, 17.8, −4.3, −5.4; HRMS (ESI, M + Na^+^) calcd for C_44_H_52_N_4_O_13_SiNa 895.3197, found 895.3193.

#### 2-Azido-4,6-*O*-benzylidene-1-*O-tert*-butyldimethylsilyl-2-deoxyl-3-*O*-(3-*O*-acetyl-4,6-*O*-benzylidene-2-deoxyl-2-phtha-limido-β-d-glucopyranosyl)-β-d-glucopyranoside (16)

A solution of acceptor 4c (100 mg, 0.25 mmol) and dried phosphotungstic acid (177 mg, 0.062 mmol) in dichloromethane (2.68 mL) was stirred for 30 minutes at 28 °C with activated 4 Å molecular sieves (335 mg). Then *N*-iodosuccinimide (166 mg, 0.74 mmol) and donor 5f (235 mg, 0.43 mmol) in dichloromethane (4 mL) was added, and the reaction mixture was stirred for 8 hours. When the reaction was completed, molecular sieves were removed by filtration through Celite. The filtrate was extracted with aqueous sodium thiosulfate (20 mL) and brine (20 mL), and the organic layers were dried over anhydrous MgSO_4_, filtered, and concentrated under vacuum. The residue was purified by column chromatography on silica gel to give the desired product 16 (176 mg, 86%) as a white soild. *R*_f_ 0.52 (EtOAc/Hex = 1/2); mp 260–262 °C; [*α*]^24^_D_ −64.3 (*c* 1.0, DCM); IR (NaCl) *ν* 3441, 2931, 2112, 1745, 1719, 1387, 1225, 1098 cm^−1^; ^1^H NMR (400 MHz, CDCl_3_) *δ* 7.85 (dd, *J* = 5.2, 3.0 Hz, 2H), 7.68 (dd, *J* = 5.4, 3.0 Hz, 2H), 7.49–7.43 (m, 4H), 7.39–7.34 (m, 6H), 5.90 (t, *J* = 9.8 Hz, 1H), 5.56 (d, *J* = 8.4 Hz, 1H), 5.50 (d, *J* = 14.8 Hz, 2H), 4.53 (d, *J* = 7.6 Hz, 1H), 4.39 (dd, *J* = 10.2, 8.6 Hz, 1H), 4.24 (dd, *J* = 10.6, 5.0 Hz, 1H), 4.07 (dd, *J* = 10.0, 4.4 Hz, 1H), 3.78–3.69 (m, 3H), 3.66 (dd, *J* = 9.4, 4.2 Hz, 1H), 3.58 (t, *J* = 9.2 Hz, 1H), 3.48 (t, *J* = 9.4 Hz, 1H), 3.31 (td, *J* = 9.6, 5.0 Hz, 1H), 3.20 (dd, *J* = 9.6, 7.6 Hz, 1H), 1.88 (s, 3H), 0.85 (s, 9H), 0.07 (s, 3H), 0.06 (s, 3H); ^13^C NMR (101 MHz, CDCl_3_) *δ* 170.0, 136.9, 136.8, 134.1, 131.3, 129.0, 128.1, 128.0, 126.2, 125.9, 123.4, 101.5, 101.1, 99.8, 97.7, 80.6, 79.0, 78.9, 69.7, 68.5, 68.2, 67.4, 66.3, 66.0, 55.4, 25.4, 20.5, 17.7, −4.5, −5.3; HRMS (ESI, M + H^+^) calcd for C_42_H_49_N_4_O_12_Si 829.3116, found 829.3113.

#### 2-Azido-4,6-*O*-benzylidene-1-*O-tert*-butyldimethylsilyl-2-deoxyl-3-*O*-(2,3,4,6-​tetra-​*O*-​acetyl-​β-d-glucopyranosyl)-β-d-glucopyranoside (17)

A solution of acceptor 4c (100 mg, 0.25 mmol) and donor 10 (167 mg, 0.37 mmol) in dichloromethane (5.5 mL) was stirred for 30 minutes at room temperature with activated 4 Å molecular sieves (323 mg). After *N*-iodosuccinimide (166 mg, 0.74 mmol) and phosphotungstic acid (216 mg, 0.075 mmol) were added, the reaction mixture was stirred for 5 hours at room temperature. When the reaction was completed, molecular sieves were removed by filtration through Celite. The filtrate was extracted with aqueous sodium thiosulfate (10 mL) and brine (10 mL), and the organic layers were dried over anhydrous MgSO_4_, filtered, and concentrated under vacuum. The residue was purified by column chromatography on silica gel to give the desired product 17 (123 mg, 67%) as a white soild. *R*_f_ 0.51 (EtOAc/Hex = 3/4); mp 96–98 °C; [*α*]^25^_D_ −40.4 (*c* 1.0, DCM); IR (NaCl) *ν* 3472, 2933, 2860, 2114, 1757, 1371, 1228, 1099, 1040 cm^−1^; ^1^H NMR (400 MHz, CDCl_3_) *δ* 7.44 (dd, *J* = 6.8, 2.9 Hz, 2H), 7.33–7.32 (m, 3H), 5.52 (s, 1H), 5.16 (d, *J* = 9.6 Hz, 1H), 5.09–5.00 (m, 2H), 4.74 (d, *J* = 8.0 Hz, 1H), 4.59 (d, *J* = 8.0 Hz, 1H), 4.25 (dd, *J* = 10.6, 5.0 Hz, 1H), 4.08 (dd, *J* = 12.4, 4.0 Hz, 1H), 3.84 (dd, *J* = 12.4, 2.4 Hz, 1H), 3.77 (t, *J* = 10.4 Hz, 1H), 3.66 (t, *J* = 9.2 Hz, 1H), 3.59 (t, *J* = 9.4 Hz, 1H), 3.47–3.43 (m, 1H), 3.36 (dd, *J* = 9.6, 4.8 Hz, 1H), 3.31 (dd, *J* = 9.6, 7.6 Hz, 1H), 2.05 (s, 3H), 1.98 (s, 3H), 1.97 (s, 3H), 1.96 (s, 3H), 0.91 (s, 9H), 0.15 (s, 3H), 0.13 (s, 3H); ^13^C NMR (101 MHz, CDCl_3_) *δ* 170.5, 170.2, 169.3, 169.2, 136.9, 129.1, 128.1, 125.9, 101.2, 101.0, 97.5, 79.4, 79.1, 72.7, 71.6, 71.4, 68.4, 68.3, 67.9, 66.4, 61.4, 25.4, 20.6, 20.5, 20.5, 17.8, −4.5, −5.3; HRMS (ESI, M + Na^+^) calcd for C_33_H_47_N_3_O_14_SiNa 760.2725, found 760.2732.

## Author contribution

S.-Y. L. designed the research. J.-S. C. and S.-S. W. prepared compounds 2a–2f of Table 1. A. S. prepared compounds 4a–4e of Table 2. J.-S. C., P.-H. H., and C.-H. L. prepared compounds 6a–6f of Table 3. Y.-J. L. and A. S. prepared compounds 13–17 of Table 4. H. R. W. analyzed the Mass data. A. S. and S.-Y. L. wrote the manuscript.

## Conflicts of interest

There are no conflicts to declare.

## Supplementary Material

RA-009-C9RA06170C-s001
