# Supplementary material for: Phosphotungstic acid as a novel acidic catalyst for carbohydrate protection and glycosylation
Source: RSC Adv. 2019 Oct 21;9(58):33853–62. doi: 10.1039/c9ra06170c (PMC9073715; doi:10.1039/c9ra06170c)
Supplement: RA-009-C9RA06170C-s001 [file RA-009-C9RA06170C-s001.pdf]

Electronic Supplementary Material (ESI) for RSC Advances.
This journal is © The Royal Society of Chemistry 2019

Supporting Information

Phosphotungstic acid as a novel acidic catalyst for carbohydrate protection and glycosylation

Jyun-Siao Chen,[a] Arumugam Sankar,[a] Yi-Jyun Lin,[a] Po-Hsun Huang,[a] Chih-Hsiang Liao,[b] Shen-Shen Wu,[c] Hsin-Ru Wu,[d] Shun-Yuan Luo[a]*

[a] Department of Chemistry, National Chung Hsing University, Taichung 402, Taiwan.

[b] Taichung Municipal Feng Yuan Senior High School, Taichung 420, Taiwan

[c] National Hsinchu Girls' Senior High School, Hsinchu 300, Taiwan.

[d] Instrumentation Center, MOST, National Tsing Hua University, Hsinchu 300, Taiwan.

E-mail: *syluo@dragon.nchu.edu.tw*

**Contents**	**Page**
NMR spectrum of important compounds	2-45

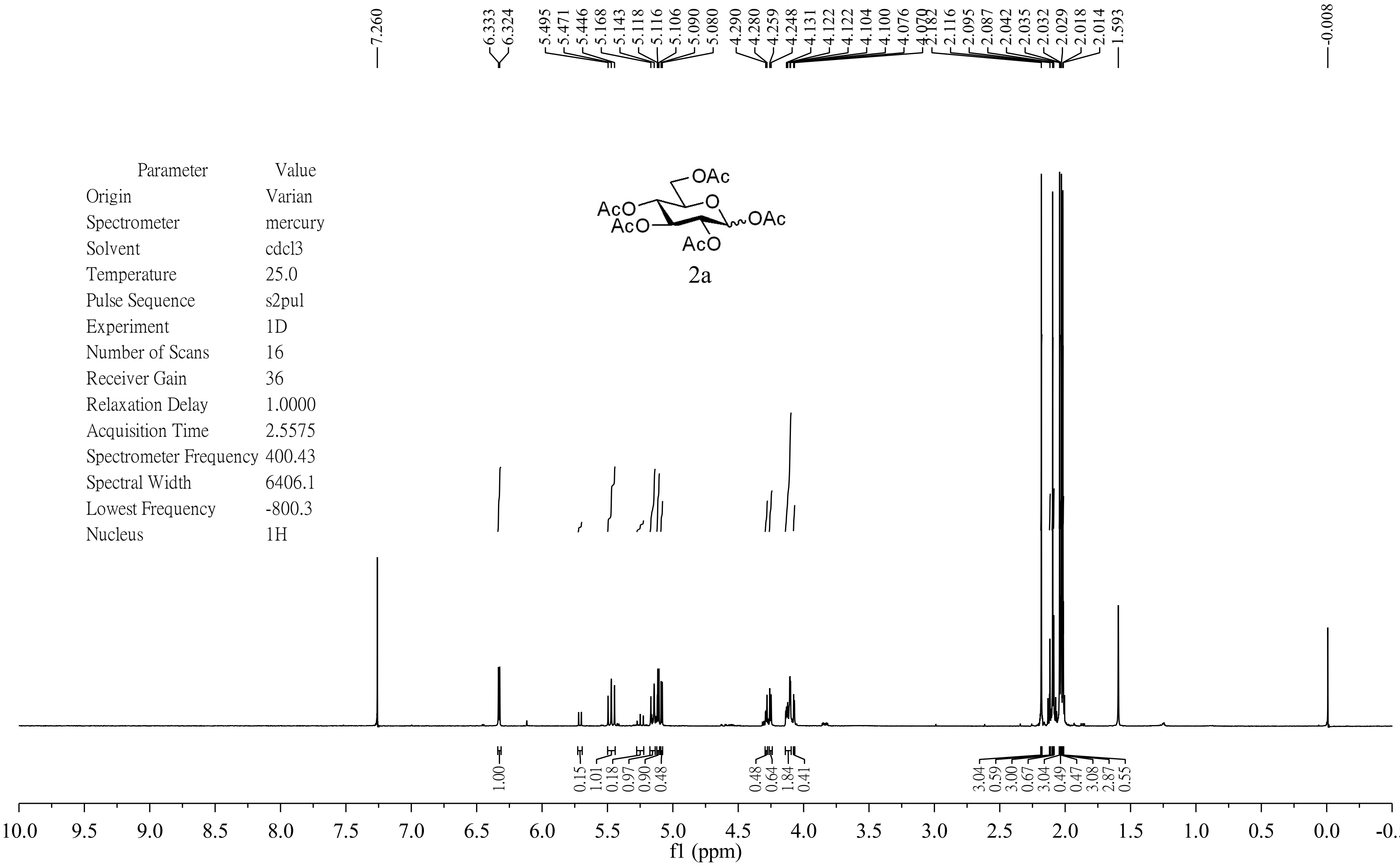

Parameter	Value
Origin	Varian
Spectrometer	mercury
Solvent	cdcl3
Temperature	25.0
Pulse Sequence	s2pul
Experiment	1D
Number of Scans	16
Receiver Gain	36
Relaxation Delay	1.0000
Acquisition Time	2.5575
Spectrometer Frequency	400.43
Spectral Width	6406.1
Lowest Frequency	-800.3
Nucleus	1H

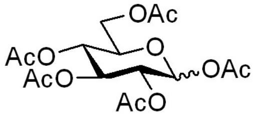

2a

Parameter	value
Origin	Varian
Spectrometer	vnmrs
Solvent	cdcl3
Temperature	20.0
Pulse Sequence	s2pul
Experiment	1D
Number of Scans	216
Receiver Gain	30
Relaxation Delay	2.0000
Acquisition Time	1.3107
Spectrometer Frequency	100.53
Spectral Width	25000.0
Lowest Frequency	-1441.7
Nucleus	13C

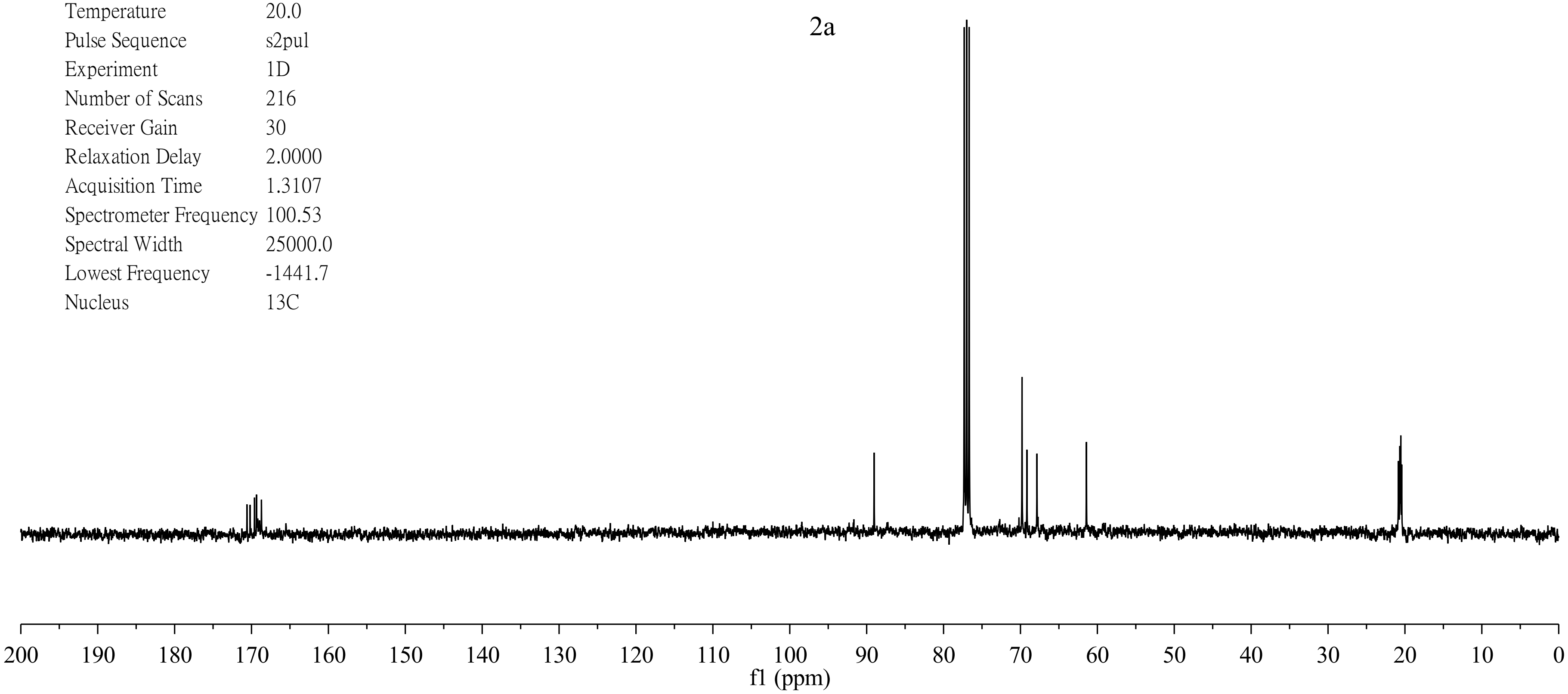

Parameter	Value
Origin	Varian
Spectrometer	mercury
Solvent	cdcl3
Temperature	25.0
Pulse Sequence	s2pul
Experiment	1D
Number of Scans	20
Receiver Gain	36
Relaxation Delay	1.0000
Acquisition Time	2.5575
Spectrometer Frequency	400.43
Spectral Width	6406.1
Lowest Frequency	-800.3
Nucleus	1H

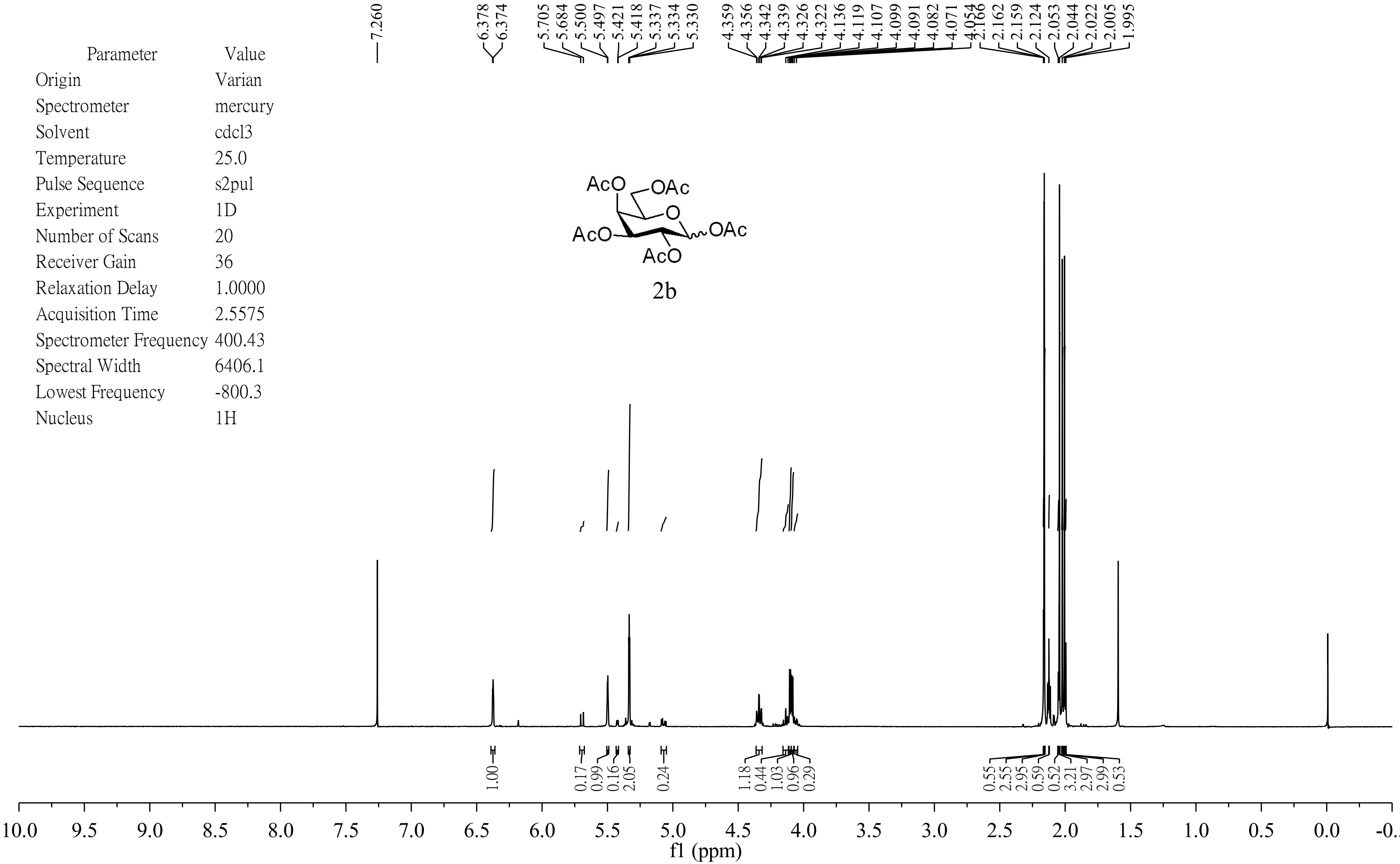

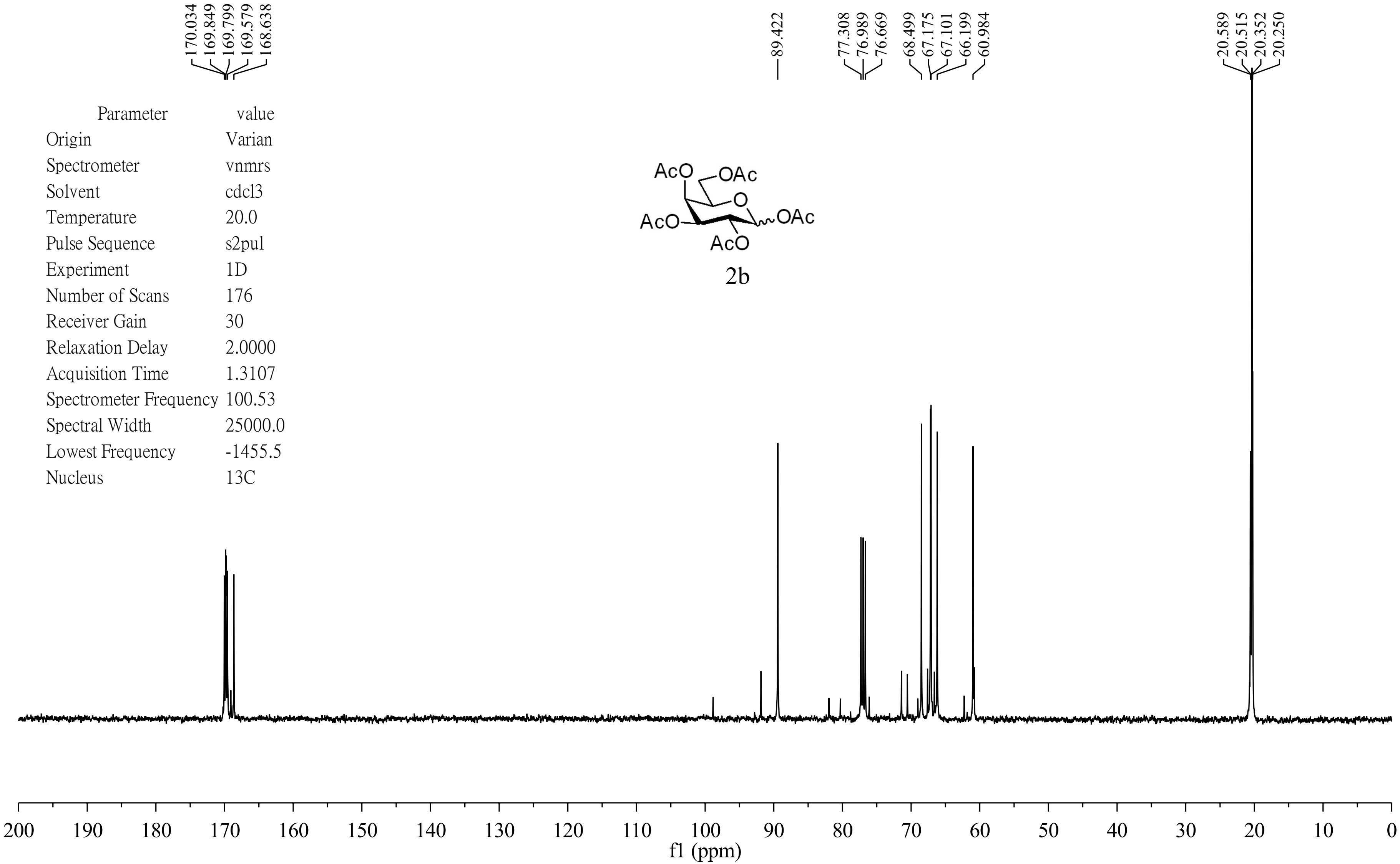

170.034
169.849
169.799
169.579
168.638
89.422
77.308
76.989
76.669
68.499
67.175
67.101
66.199
60.984
20.589
20.515
20.352
20.250
Parameter value
Origin Varian
Spectrometer vnmrs
Solvent cdcl3
Temperature 20.0
Pulse Sequence s2pul
Experiment 1D
Number of Scans 176
Receiver Gain 30
Relaxation Delay 2.0000
Acquisition Time 1.3107
Spectrometer Frequency 100.53
Spectral Width 25000.0
Lowest Frequency -1455.5
Nucleus 13C
AcO
OAc
O
AcO
OAc
AcO
2b
200 190 180 170 160 150 140 130 120 110 100 90 80 70 60 50 40 30 20 10 0
f1 (ppm)

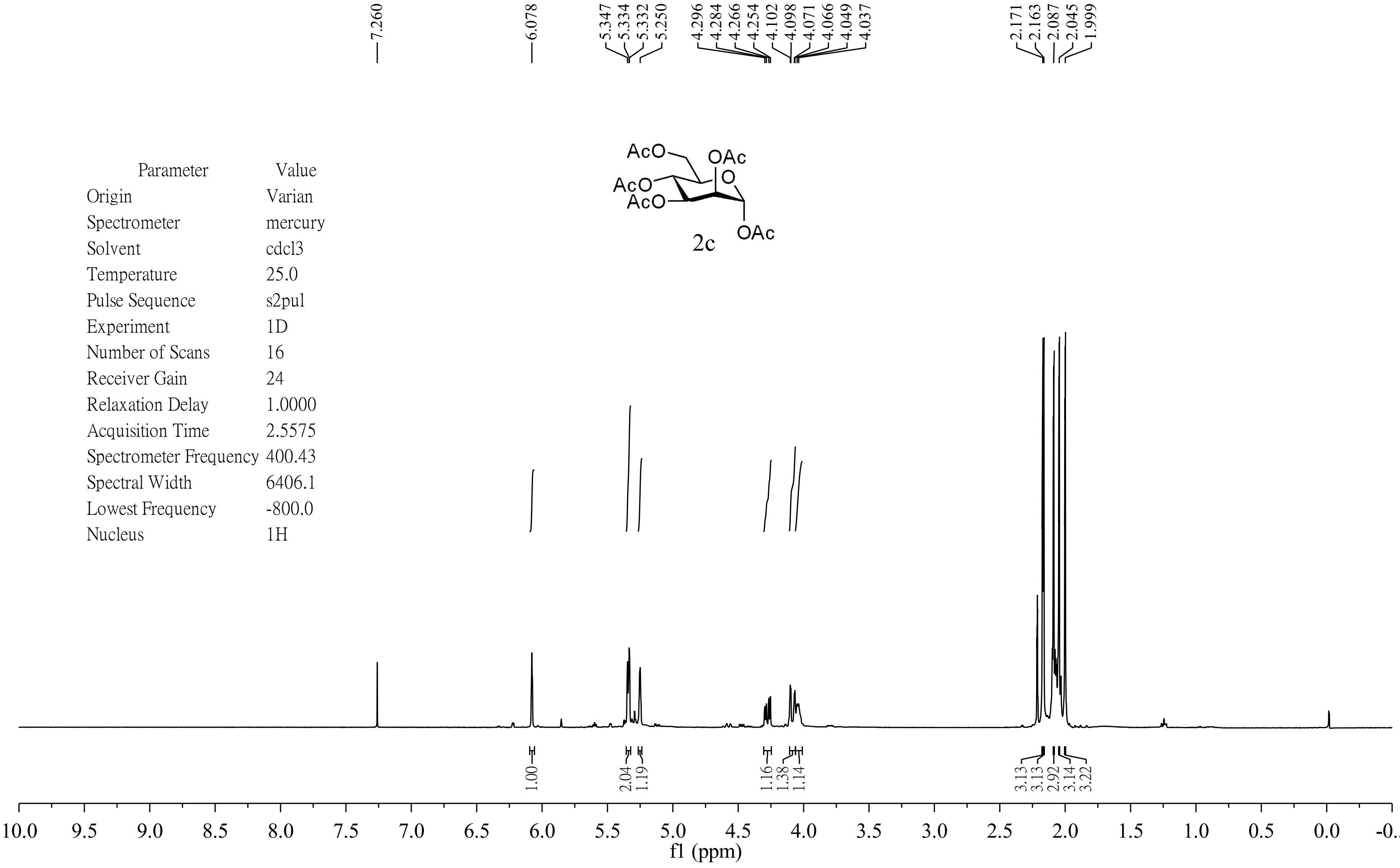
7.260
6.078
5.347
5.334
5.332
5.250
4.296
4.284
4.266
4.254
4.102
4.098
4.071
4.066
4.049
4.037
2.171
2.163
2.087
2.045
1.999
AcO
OAc
O
AcO
AcO
OAc
2c
Parameter Value
Origin Varian
Spectrometer mercury
Solvent cdcl3
Temperature 25.0
Pulse Sequence s2pul
Experiment 1D
Number of Scans 16
Receiver Gain 24
Relaxation Delay 1.0000
Acquisition Time 2.5575
Spectrometer Frequency 400.43
Spectral Width 6406.1
Lowest Frequency -800.0
Nucleus 1H
1.00
2.04
1.19
1.16
1.38
1.14
3.13
3.13
2.92
3.14
3.22
10.0 9.5 9.0 8.5 8.0 7.5 7.0 6.5 6.0 5.5 5.0 4.5 4.0 3.5 3.0 2.5 2.0 1.5 1.0 0.5 0.0 -0.5
f1 (ppm)

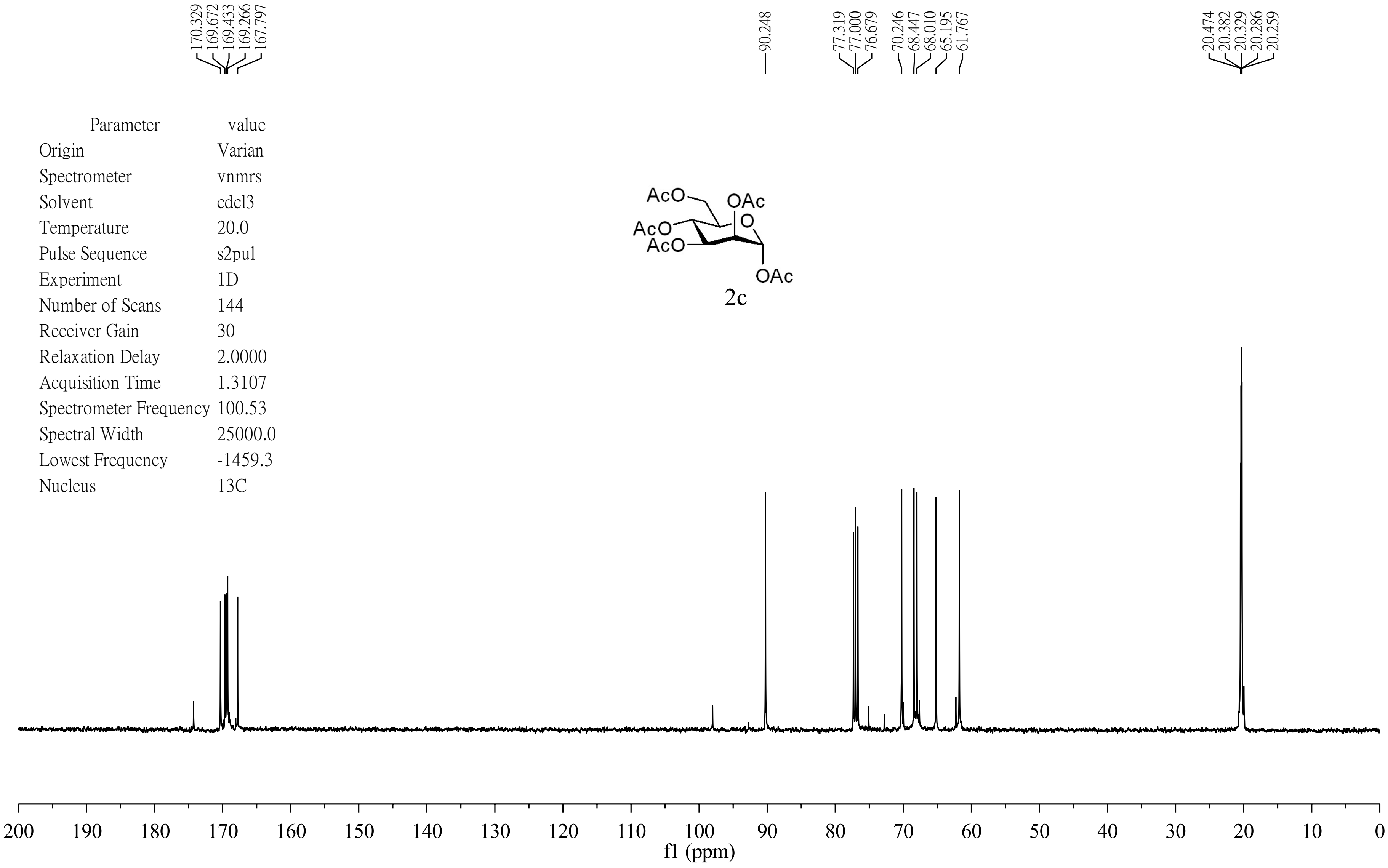

Parameter	value
Origin	Varian
Spectrometer	vnmrs
Solvent	cdcl3
Temperature	20.0
Pulse Sequence	s2pul
Experiment	1D
Number of Scans	144
Receiver Gain	30
Relaxation Delay	2.0000
Acquisition Time	1.3107
Spectrometer Frequency	100.53
Spectral Width	25000.0
Lowest Frequency	-1459.3
Nucleus	13C

2c

Parameter	Value
Origin	Varian
Spectrometer	mercury
Solvent	cdcl3
Temperature	25.0
Pulse Sequence	s2pul
Experiment	1D
Number of Scans	16
Receiver Gain	30
Relaxation Delay	1.0000
Acquisition Time	2.5575
Spectrometer Frequency	400.43
Spectral Width	6406.1
Lowest Frequency	-800.0
Nucleus	1H

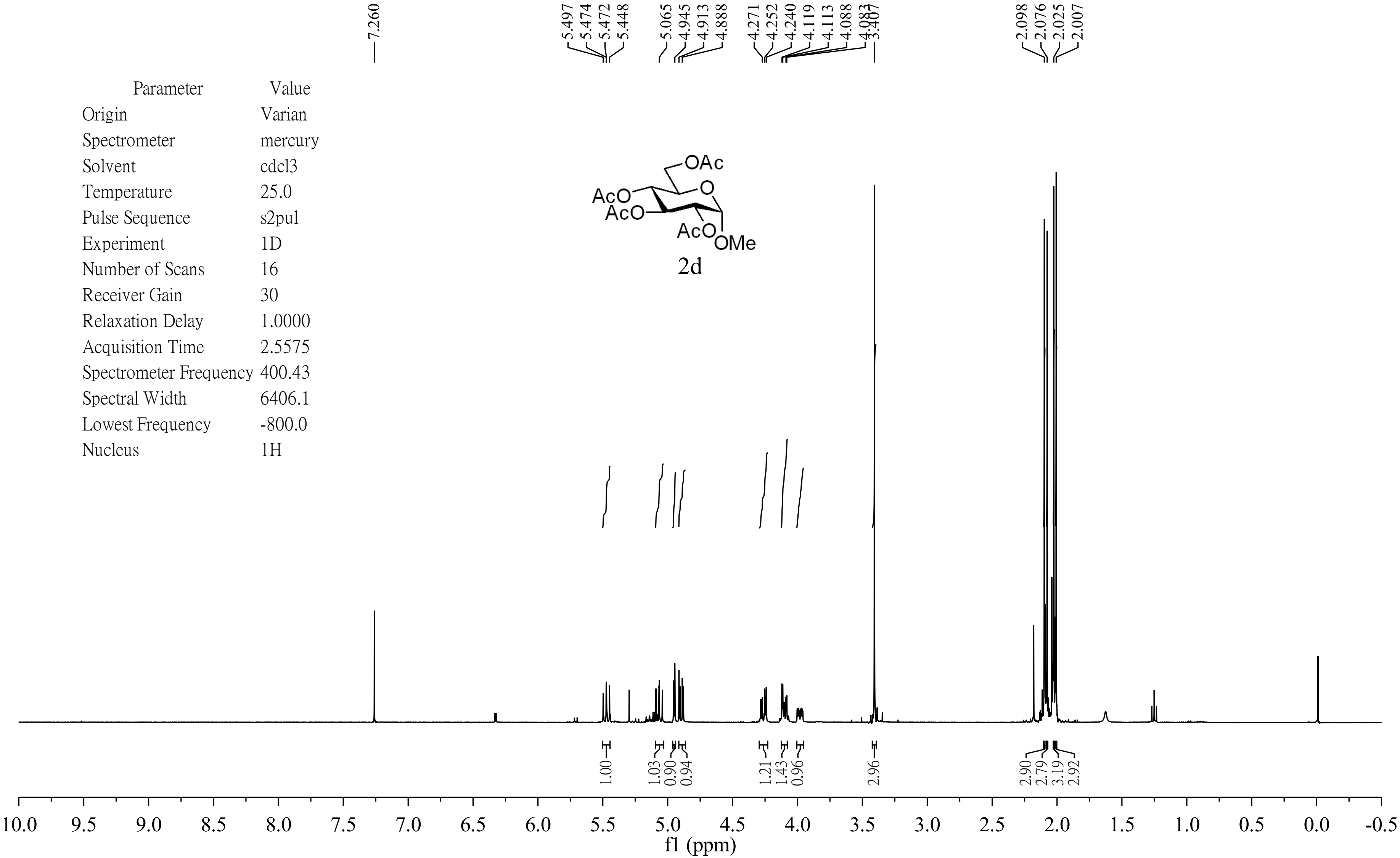

2d

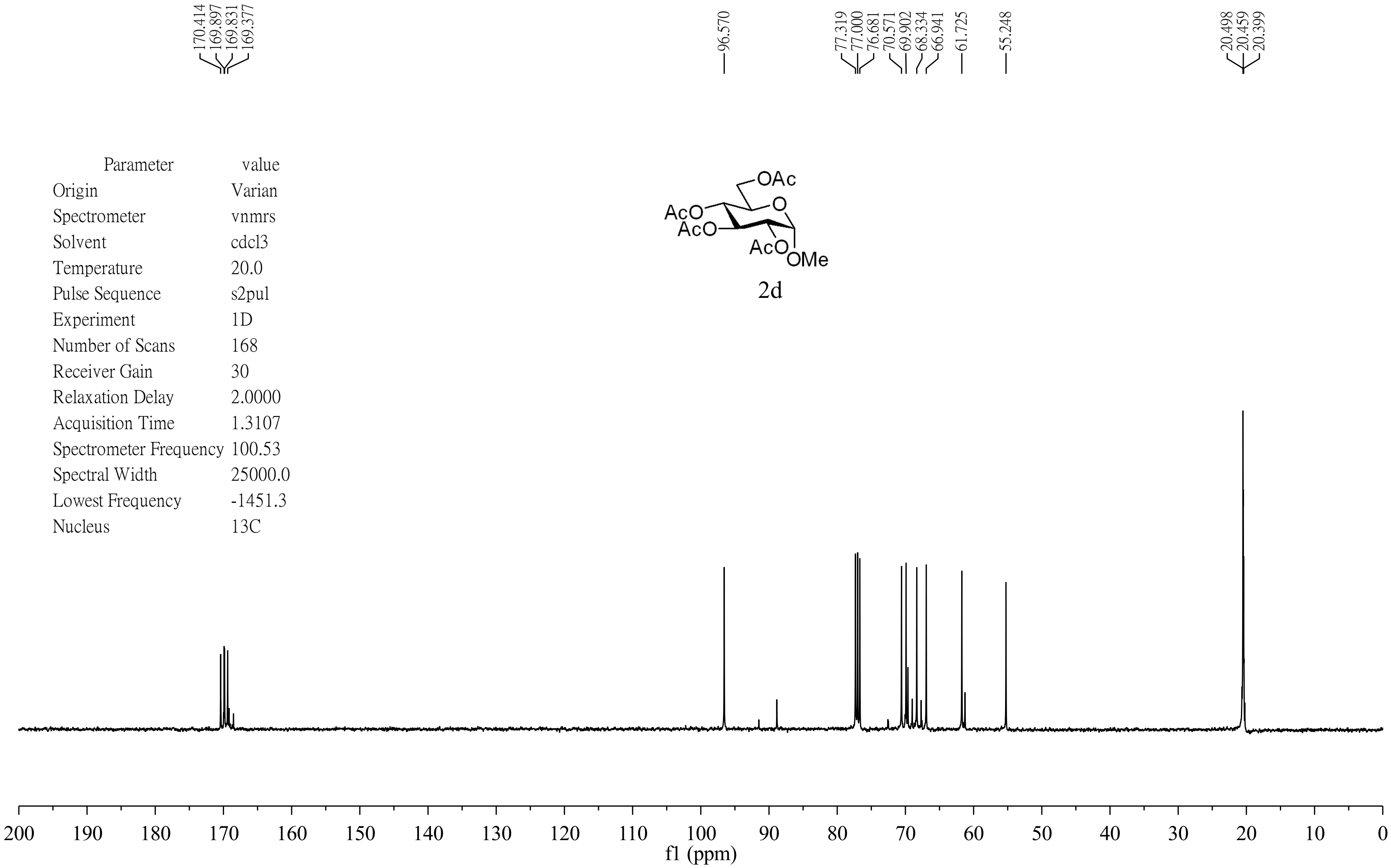

Parameter	value
Origin	Varian
Spectrometer	vnmrs
Solvent	cdcl3
Temperature	20.0
Pulse Sequence	s2pul
Experiment	1D
Number of Scans	168
Receiver Gain	30
Relaxation Delay	2.0000
Acquisition Time	1.3107
Spectrometer Frequency	100.53
Spectral Width	25000.0
Lowest Frequency	-1451.3
Nucleus	13C

2d

Parameter	Value
Origin	Varian
Spectrometer	mercury
Solvent	cdcl3
Temperature	25.0
Pulse Sequence	s2pul
Experiment	1D
Number of Scans	28
Receiver Gain	26
Relaxation Delay	1.0000
Acquisition Time	2.5575
Spectrometer Frequency	400.43
Spectral Width	6406.1
Lowest Frequency	-800.0
Nucleus	1H

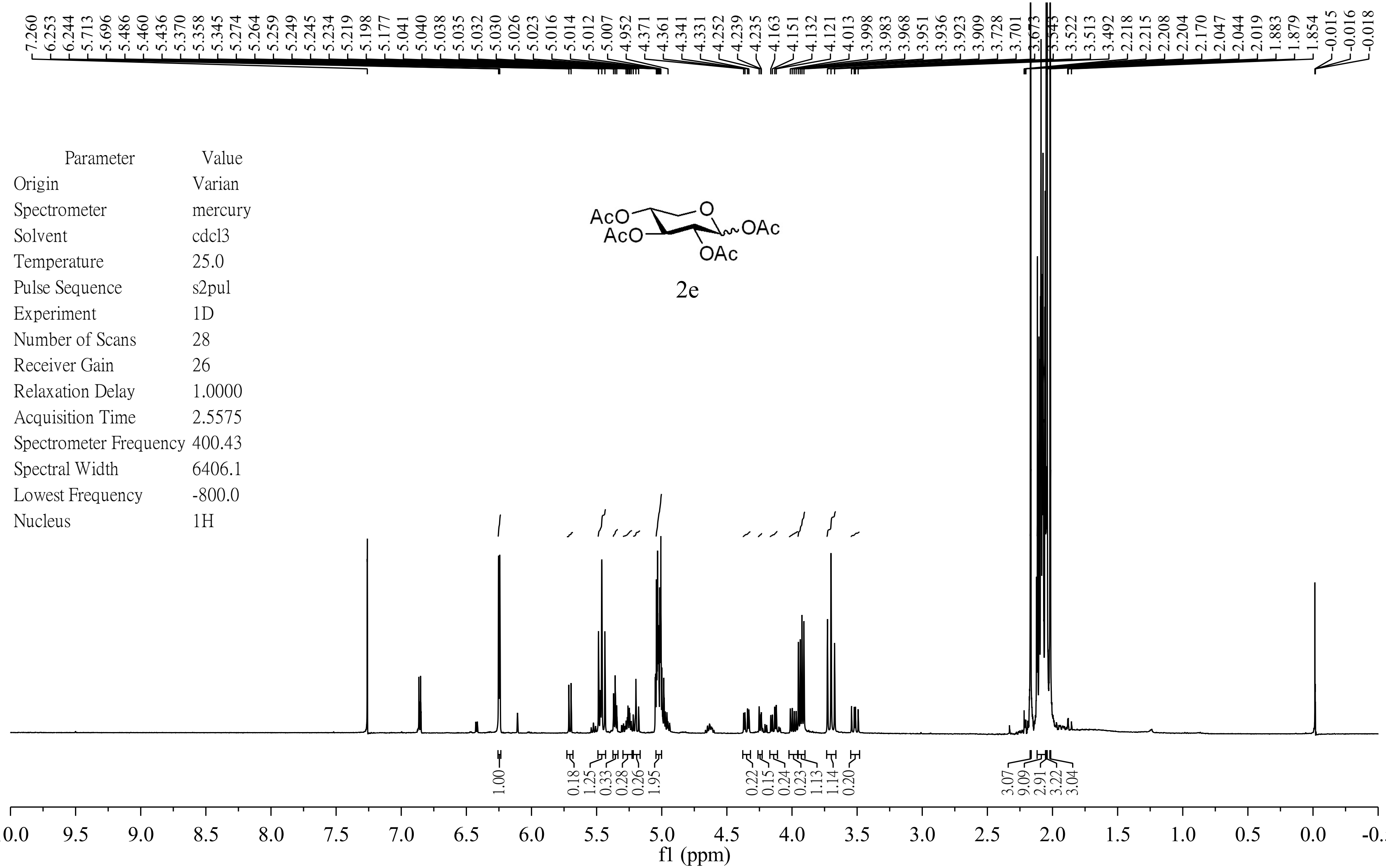

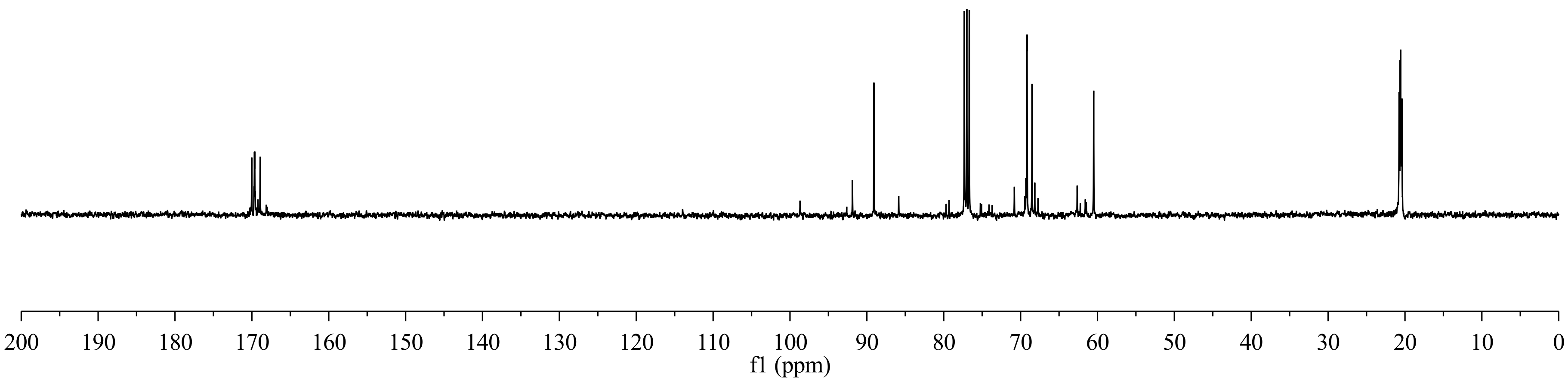

Parameter	value
Origin	Varian
Spectrometer	vnmrs
Solvent	cdcl3
Temperature	20.0
Pulse Sequence	s2pul
Experiment	1D
Number of Scans	96
Receiver Gain	30
Relaxation Delay	2.0000
Acquisition Time	1.3107
Spectrometer Frequency	100.53
Spectral Width	25000.0
Lowest Frequency	-1449.0
Nucleus	13C

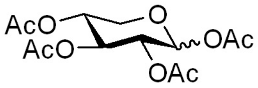

2e

Parameter	value
Origin	Varian
Spectrometer	vnmrs
Solvent	cdcl3
Temperature	20.0
Pulse Sequence	s2pul
Experiment	1D
Number of Scans	40
Receiver Gain	48
Relaxation Delay	1.0000
Acquisition Time	2.5559
Spectrometer Frequency	399.76
Spectral Width	6410.3
Lowest Frequency	-799.4
Nucleus	1H

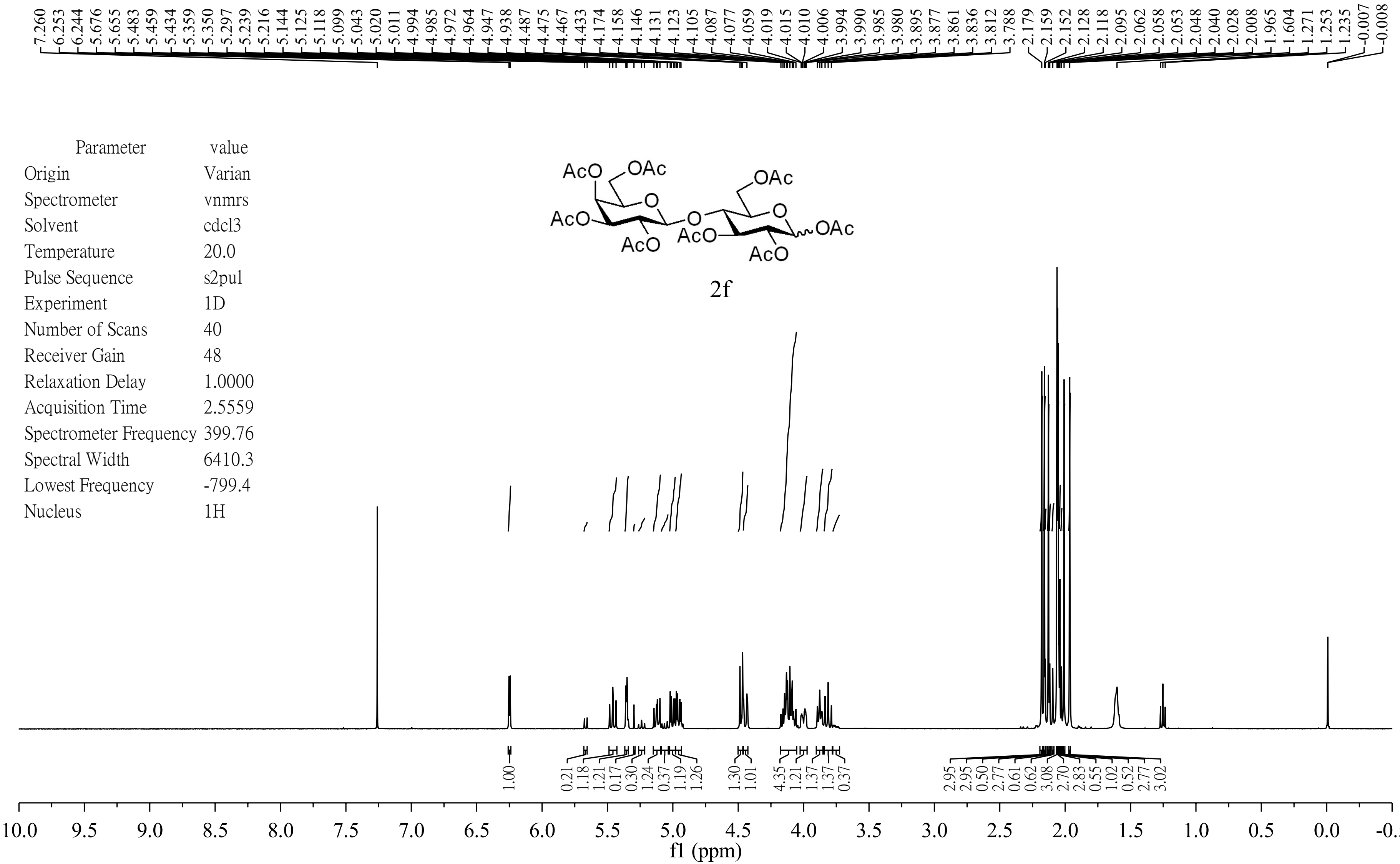

2f

Parameter	value
Origin	Varian
Spectrometer	vnmrs
Solvent	cdcl3
Temperature	20.0
Pulse Sequence	s2pul
Experiment	1D
Number of Scans	248
Receiver Gain	30
Relaxation Delay	2.0000
Acquisition Time	1.3107
Spectrometer Frequency	100.53
Spectral Width	25000.0
Lowest Frequency	-1445.9
Nucleus	13C

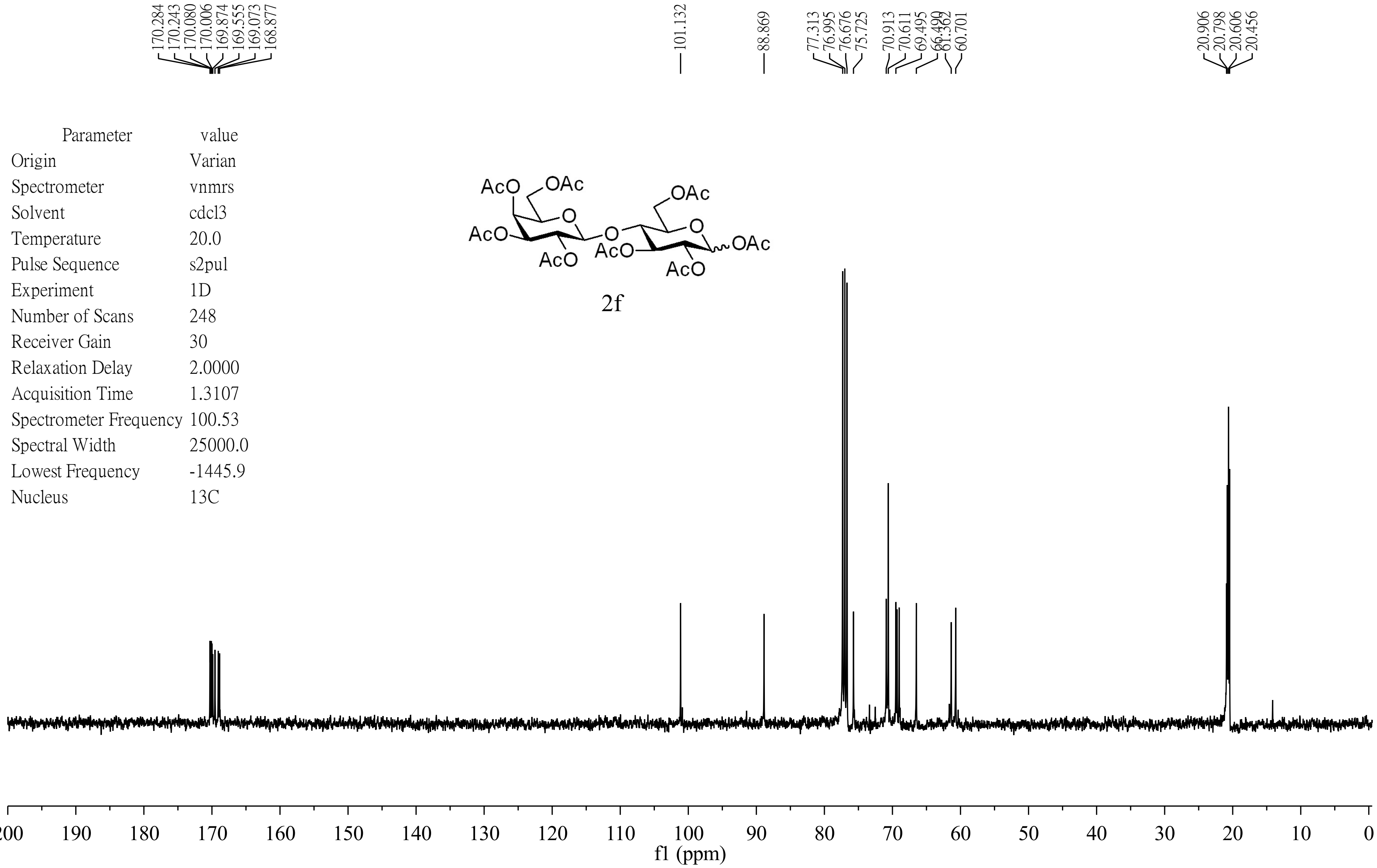

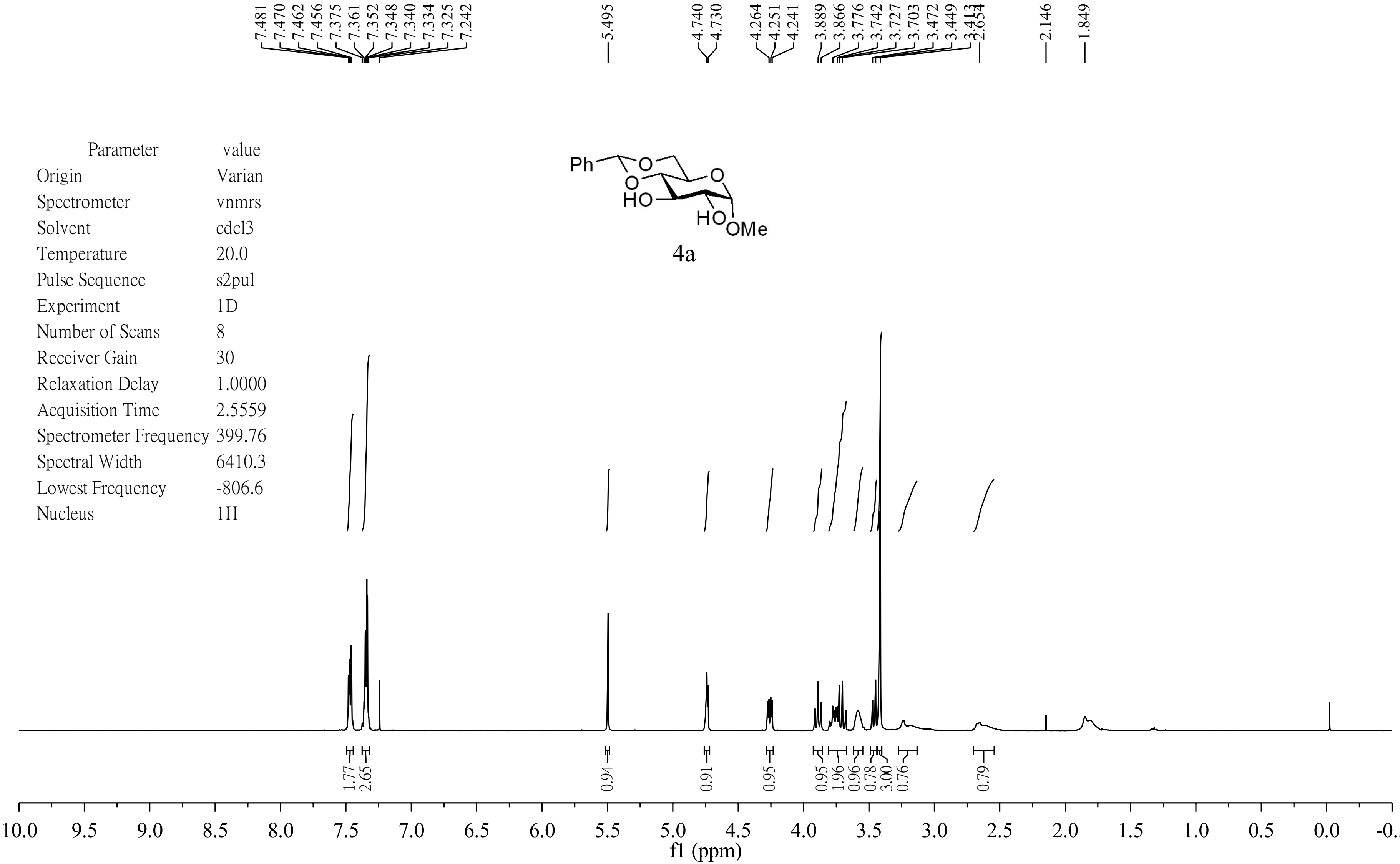
7.481 7.470 7.462 7.456 7.375 7.361 7.352 7.348 7.340 7.334 7.325 7.242
5.495
4.740 4.730
4.264 4.251 4.241
3.889 3.866 3.776 3.742 3.727 3.703 3.472 3.449 3.413 2.654
2.146
1.849
Parameter value
Origin Varian
Spectrometer vnmrs
Solvent cdcl3
Temperature 20.0
Pulse Sequence s2pul
Experiment 1D
Number of Scans 8
Receiver Gain 30
Relaxation Delay 1.0000
Acquisition Time 2.5559
Spectrometer Frequency 399.76
Spectral Width 6410.3
Lowest Frequency -806.6
Nucleus 1H
Ph
O
O
O
HO
HO
OMe
4a
1.77 2.65 0.94 0.91 0.95 0.95 1.96 0.96 0.78 3.00 0.76 0.79
10.0 9.5 9.0 8.5 8.0 7.5 7.0 6.5 6.0 5.5 5.0 4.5 4.0 3.5 3.0 2.5 2.0 1.5 1.0 0.5 0.0 -0.5
f1 (ppm)

Parameters

	Parameter	Value
1	Origin	Varian
2	Spectrometer	vnmrs
3	Solvent	cdcl3
4	Temperature	20.0
5	Pulse Sequence	s2pul
6	Experiment	1D
7	Number of Scans	1072
8	Receiver Gain	30
9	Relaxation Delay	2.0000
10	Acquisition Time	1.3107
11	Spectrometer Frequency	100.53
12	Spectral Width	25000.0
13	Lowest Frequency	-1413.9
14	Nucleus	13C

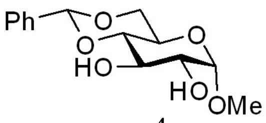

4a

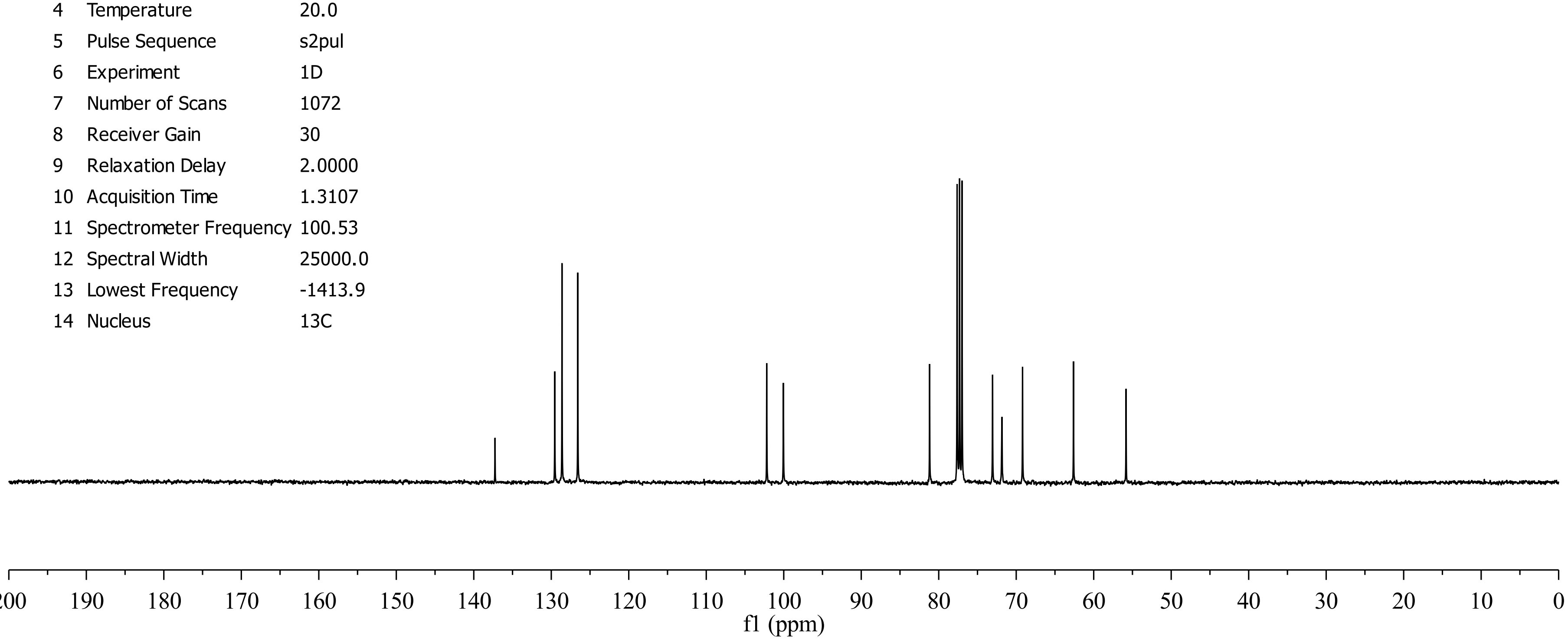

	Parameter	Value
1	Origin	Varian
2	Spectrometer	vnmrs
3	Solvent	cdcl3
4	Temperature	20.0
5	Pulse Sequence	s2pul
6	Experiment	1D
7	Number of Scans	20
8	Receiver Gain	30
9	Relaxation Delay	1.0000
10	Acquisition Time	2.5559
11	Spectrometer Frequency	399.76
12	Spectral Width	6410.3
13	Lowest Frequency	-806.6
14	Nucleus	1H

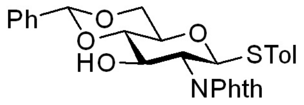

4b

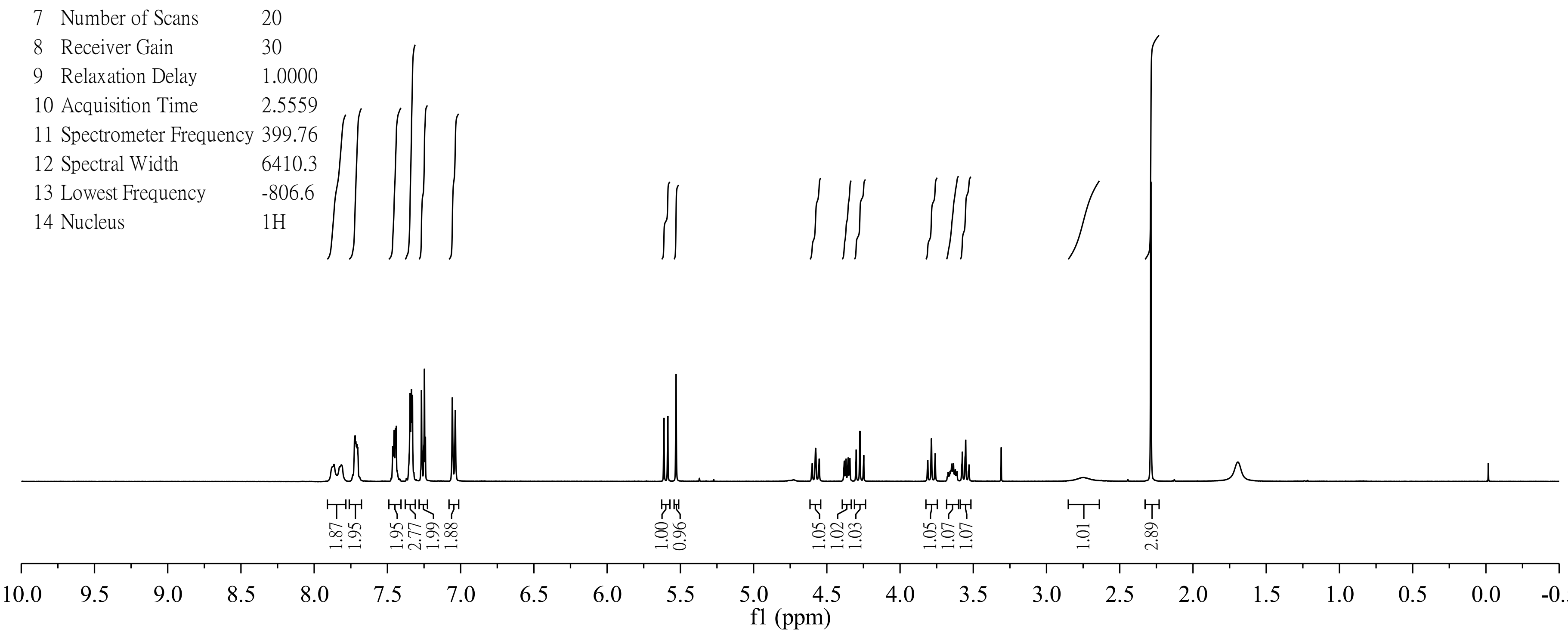

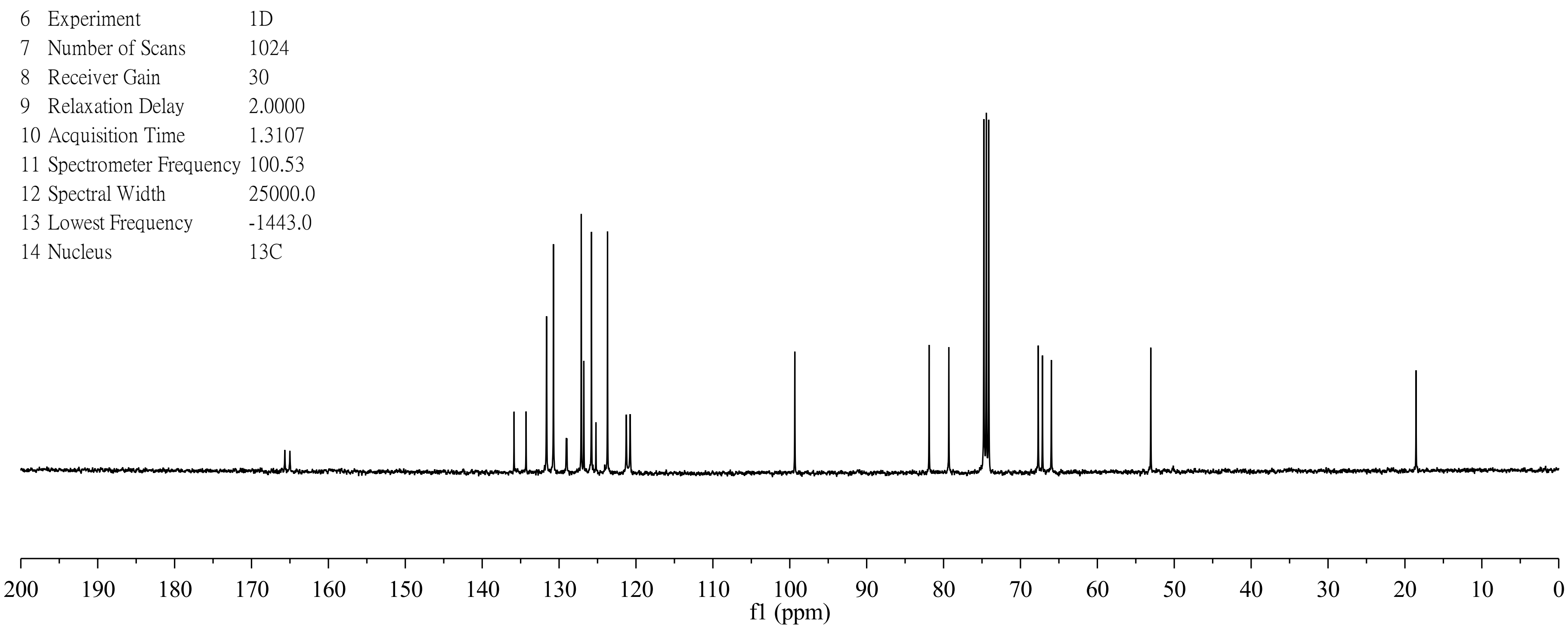

	Parameter	Value
1	Origin	Varian
2	Spectrometer	vnmrs
3	Solvent	D2O
4	Temperature	20.0
5	Pulse Sequence	s2pul
6	Experiment	1D
7	Number of Scans	1024
8	Receiver Gain	30
9	Relaxation Delay	2.0000
10	Acquisition Time	1.3107
11	Spectrometer Frequency	100.53
12	Spectral Width	25000.0
13	Lowest Frequency	-1443.0
14	Nucleus	13C

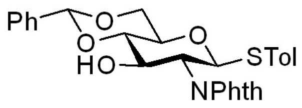

4b

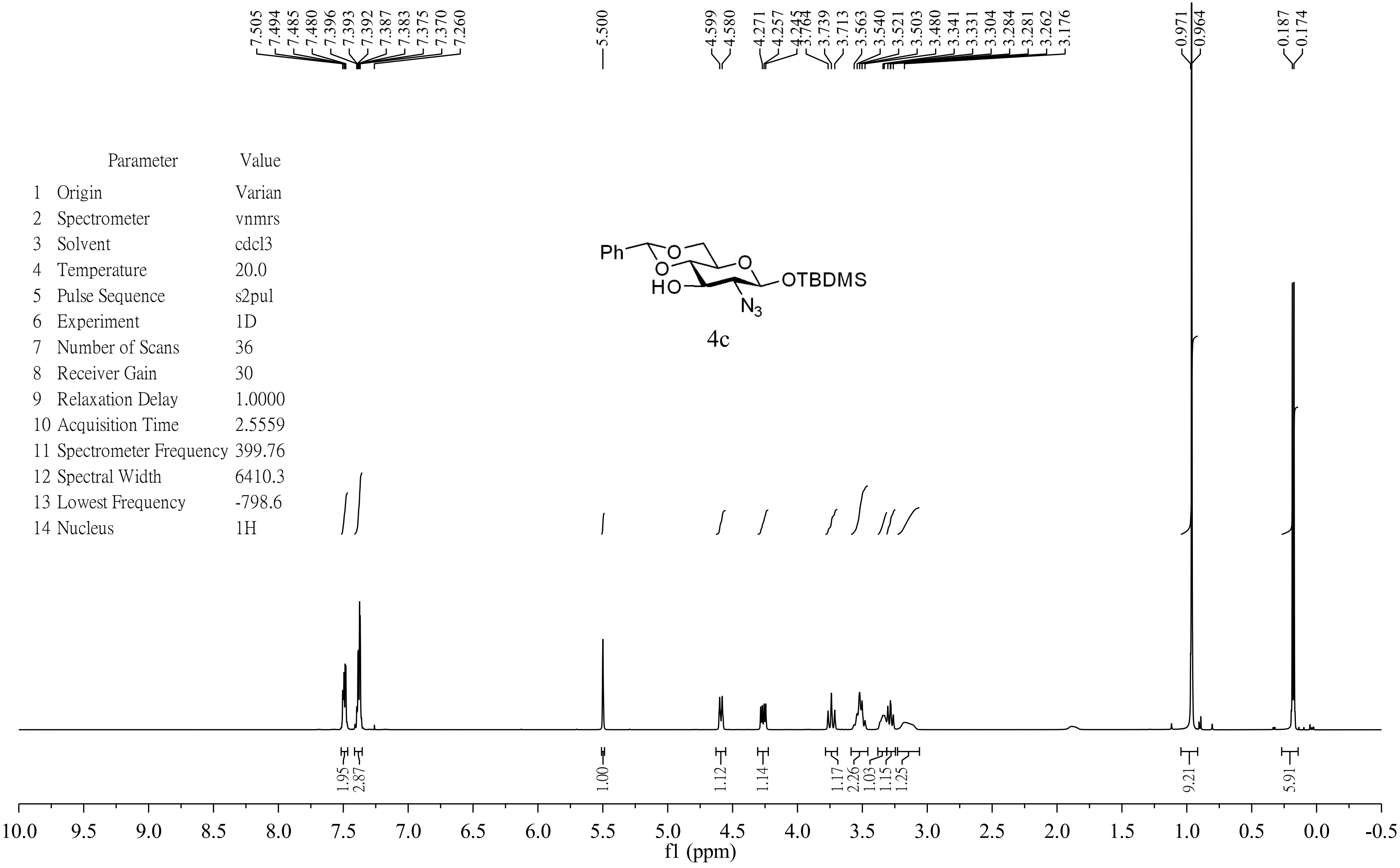

	Parameter	Value
1	Origin	Varian
2	Spectrometer	vnmrs
3	Solvent	cdcl3
4	Temperature	20.0
5	Pulse Sequence	s2pul
6	Experiment	1D
7	Number of Scans	36
8	Receiver Gain	30
9	Relaxation Delay	1.0000
10	Acquisition Time	2.5559
11	Spectrometer Frequency	399.76
12	Spectral Width	6410.3
13	Lowest Frequency	-798.6
14	Nucleus	1H

4c

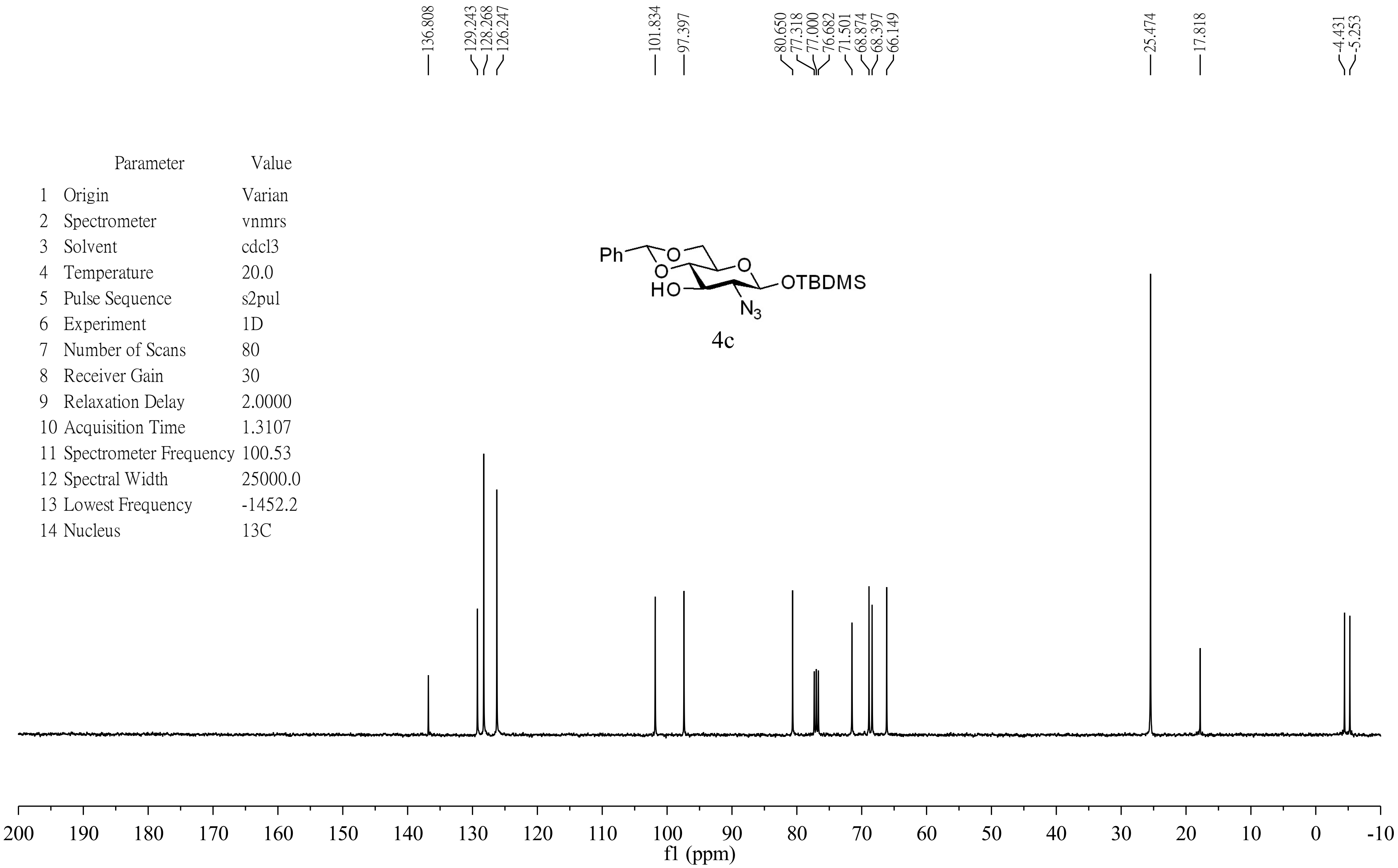

	Parameter	Value
1	Origin	Varian
2	Spectrometer	vnmrs
3	Solvent	cdcl3
4	Temperature	20.0
5	Pulse Sequence	s2pul
6	Experiment	1D
7	Number of Scans	80
8	Receiver Gain	30
9	Relaxation Delay	2.0000
10	Acquisition Time	1.3107
11	Spectrometer Frequency	100.53
12	Spectral Width	25000.0
13	Lowest Frequency	-1452.2
14	Nucleus	13C

4c

Parameter	value
Origin	Varian
Spectrometer	mercury
Solvent	cdcl3
Temperature	25.0
Pulse Sequence	s2pul
Experiment	1D
Number of Scans	80
Receiver Gain	39
Relaxation Delay	1.0000
Acquisition Time	2.5575
Spectrometer Frequency	400.43
Spectral Width	6406.1
Lowest Frequency	-801.5
Nucleus	1H

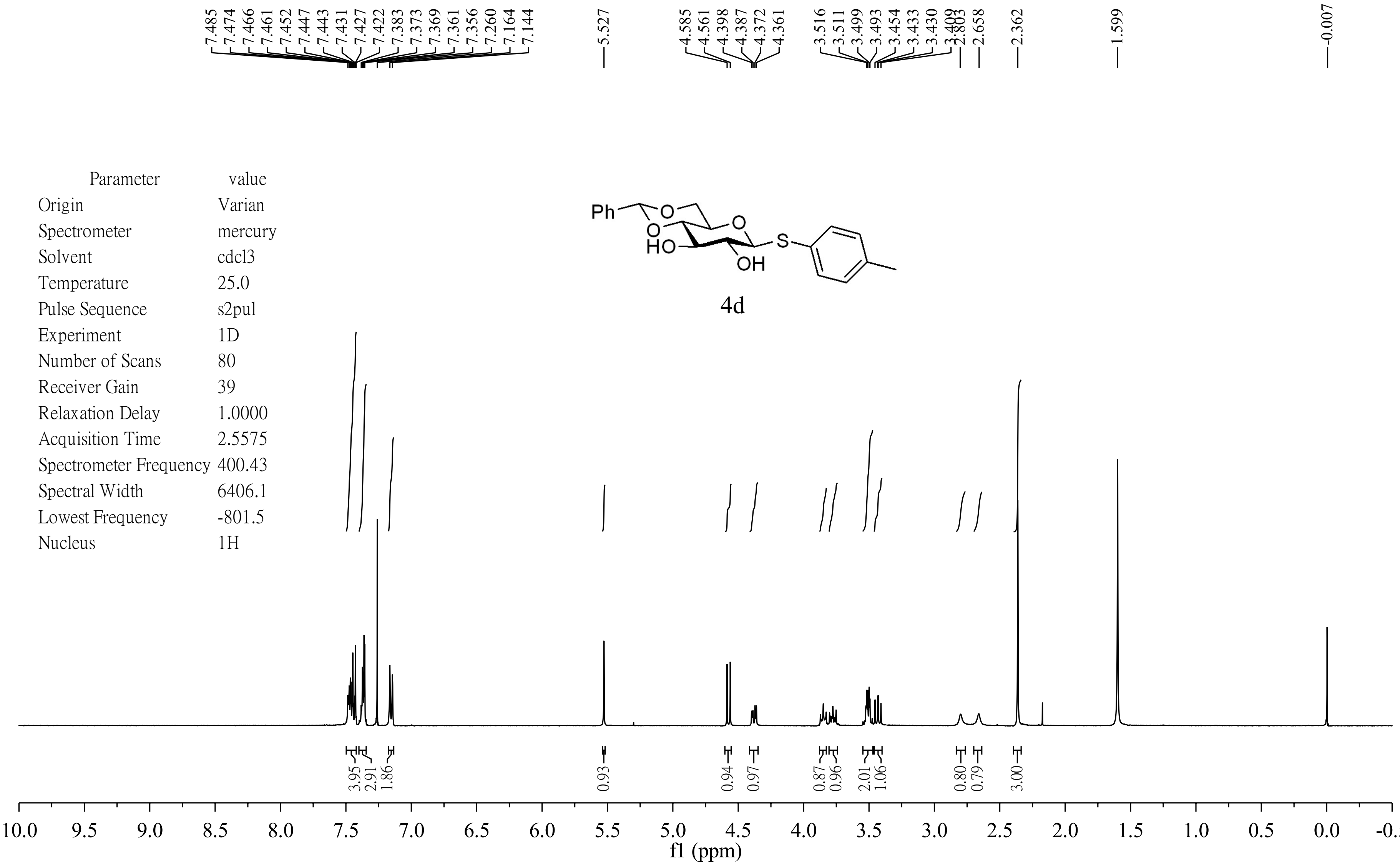

Parameter	value
Origin	Varian
Spectrometer	vnmrs
Solvent	cdcl3
Temperature	20.0
Pulse Sequence	s2pul
Experiment	1D
Number of Scans	1204
Receiver Gain	30
Relaxation Delay	2.0000
Acquisition Time	1.3107
Spectrometer Frequency	100.53
Spectral Width	25000.0
Lowest Frequency	-1450.9
Nucleus	13C

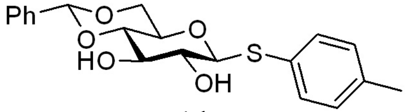

4d

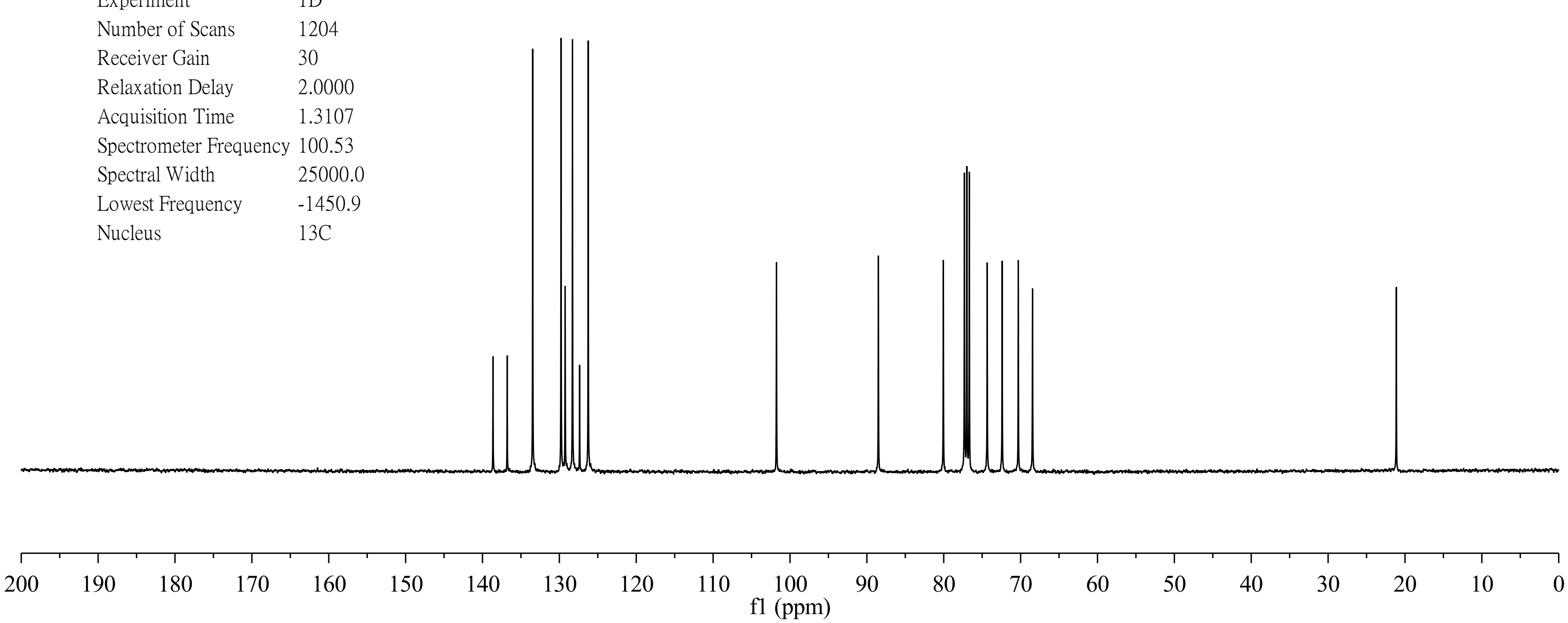

Parameter	value
Origin	Varian
Spectrometer	mercury
Solvent	cdcl3
Temperature	25.0
Pulse Sequence	s2pul
Experiment	1D
Number of Scans	104
Receiver Gain	30
Relaxation Delay	1.0000
Acquisition Time	2.5575
Spectrometer Frequency	400.43
Spectral Width	6406.1
Lowest Frequency	-846.9
Nucleus	1H

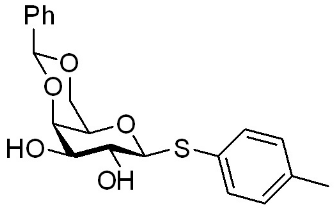

4e

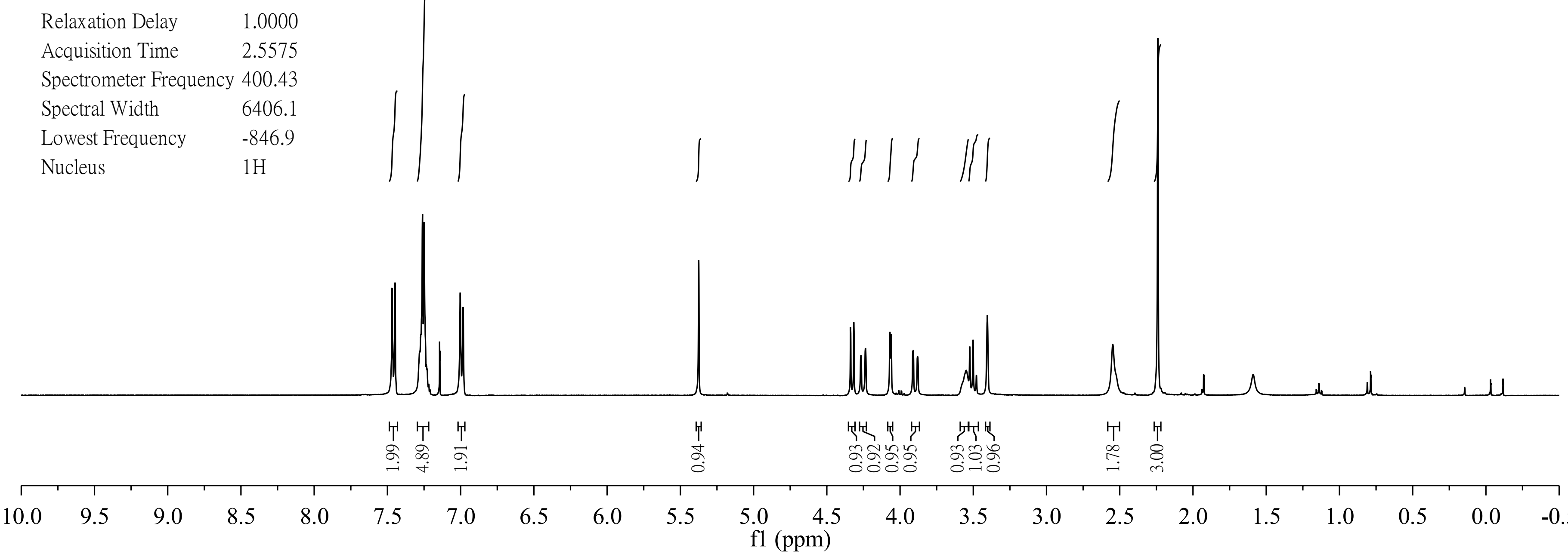

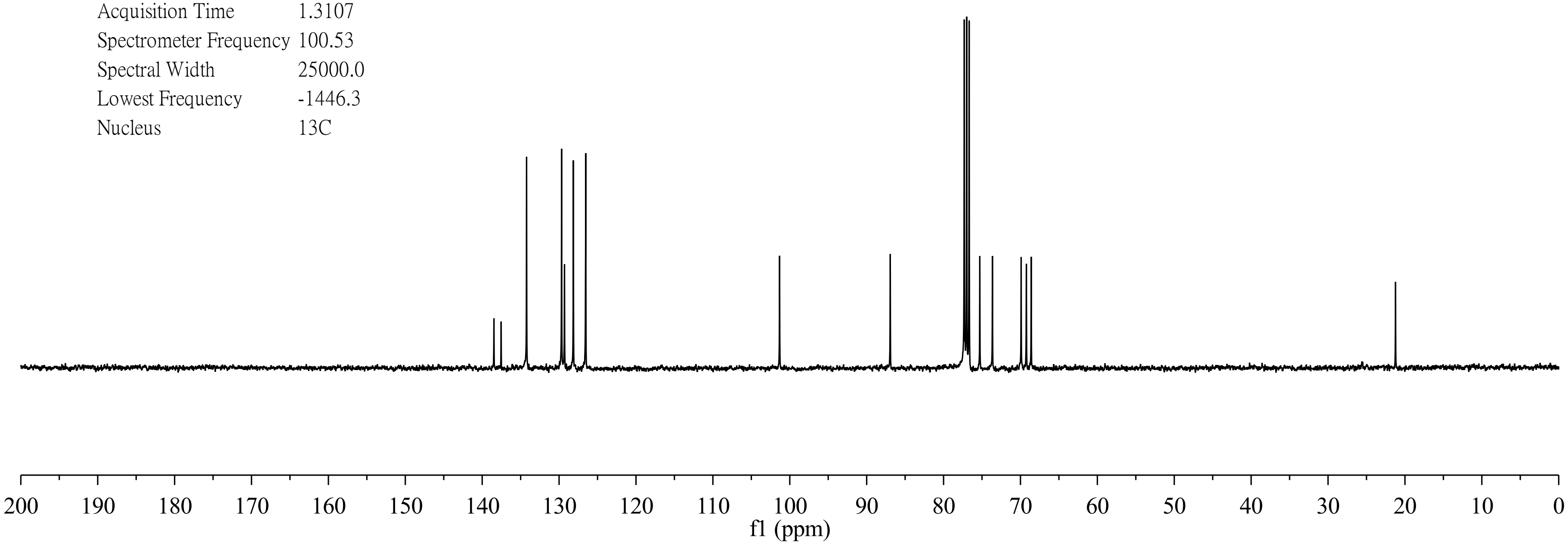

Parameter	value
Origin	Varian
Spectrometer	vnmrs
Solvent	cdcl3
Temperature	20.0
Pulse Sequence	s2pul
Experiment	1D
Number of Scans	732
Receiver Gain	30
Relaxation Delay	2.0000
Acquisition Time	1.3107
Spectrometer Frequency	100.53
Spectral Width	25000.0
Lowest Frequency	-1446.3
Nucleus	13C

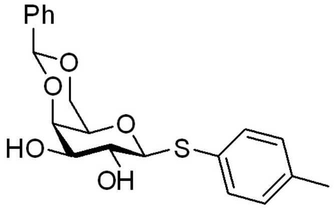

4e

Parameter	value
Origin	Varian
Spectrometer	mercury
Solvent	cdcl3
Temperature	25.0
Pulse Sequence	s2pul
Experiment	1D
Number of Scans	28
Receiver Gain	39
Relaxation Delay	1.0000
Acquisition Time	2.5575
Spectrometer Frequency	400.43
Spectral Width	6406.1
Lowest Frequency	-800.3
Nucleus	1H

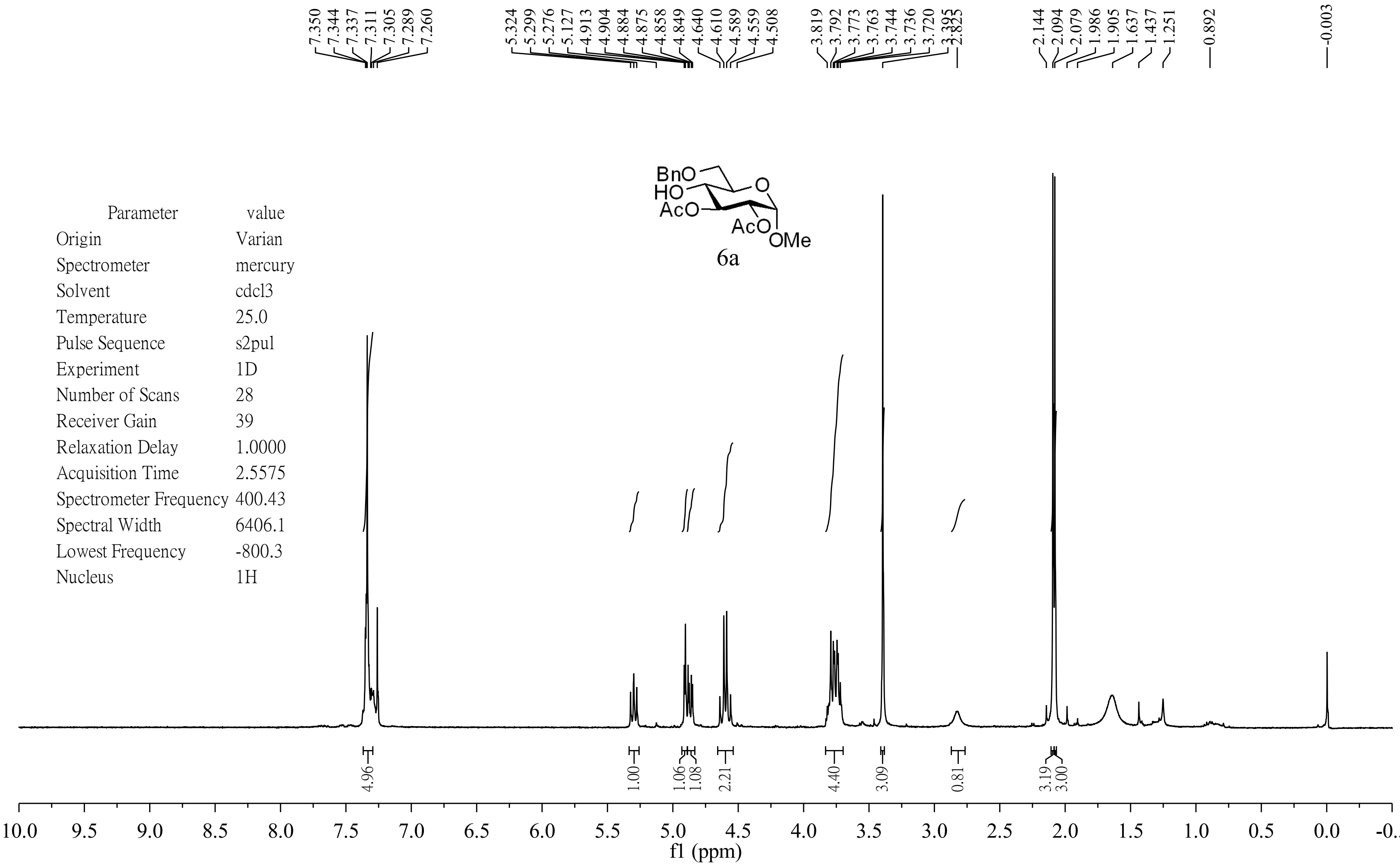

Parameter	value
Origin	Varian
Spectrometer	vnmrs
Solvent	cdcl3
Temperature	20.0
Pulse Sequence	s2pul
Experiment	1D
Number of Scans	120
Receiver Gain	30
Relaxation Delay	2.0000
Acquisition Time	1.3107
Spectrometer Frequency	100.53
Spectral Width	25000.0
Lowest Frequency	-1454.3
Nucleus	13C

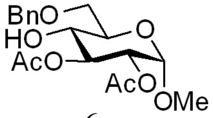

6a

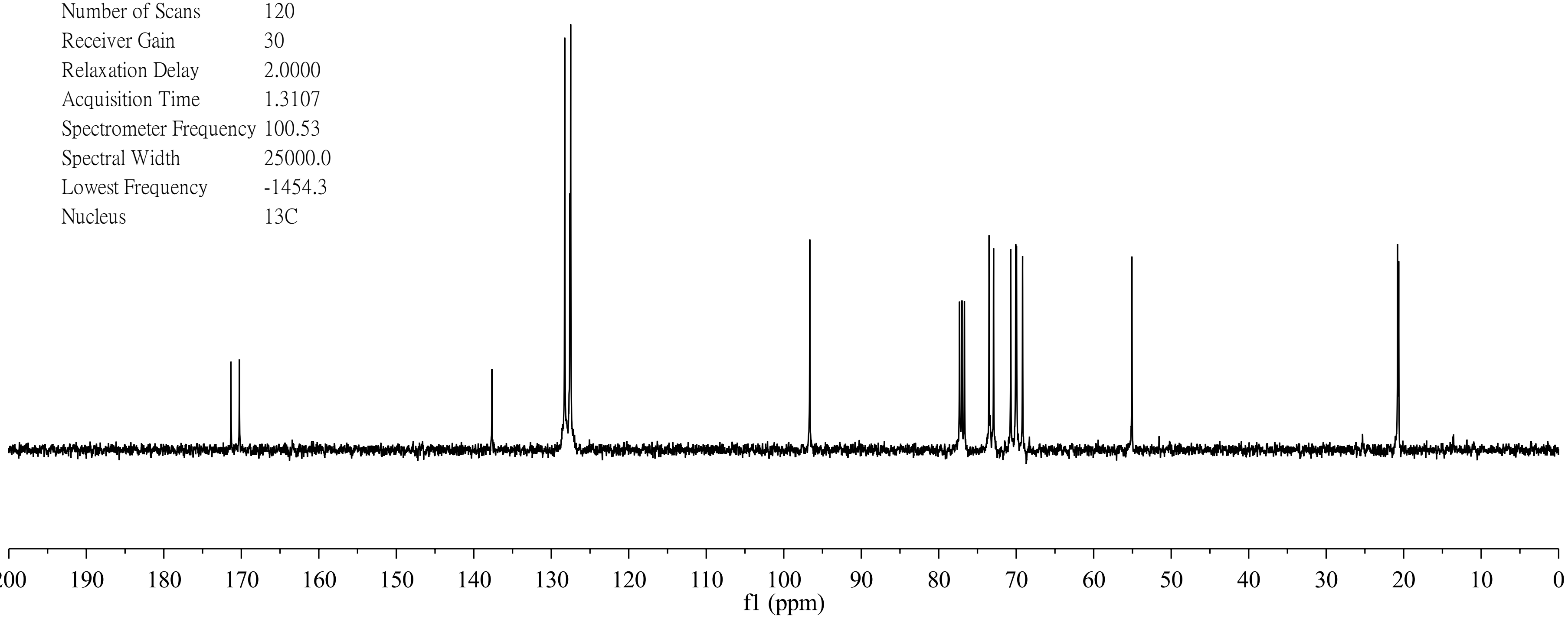

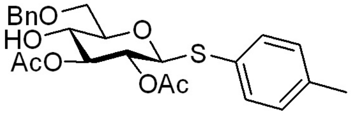

6b

Parameter	value
Origin	Varian
Spectrometer	mercury
Solvent	cdcl3
Temperature	25.0
Pulse Sequence	s2pul
Experiment	1D
Number of Scans	24
Receiver Gain	39
Relaxation Delay	1.0000
Acquisition Time	2.5575
Spectrometer Frequency	400.43
Spectral Width	6406.1
Lowest Frequency	-800.0
Nucleus	1H

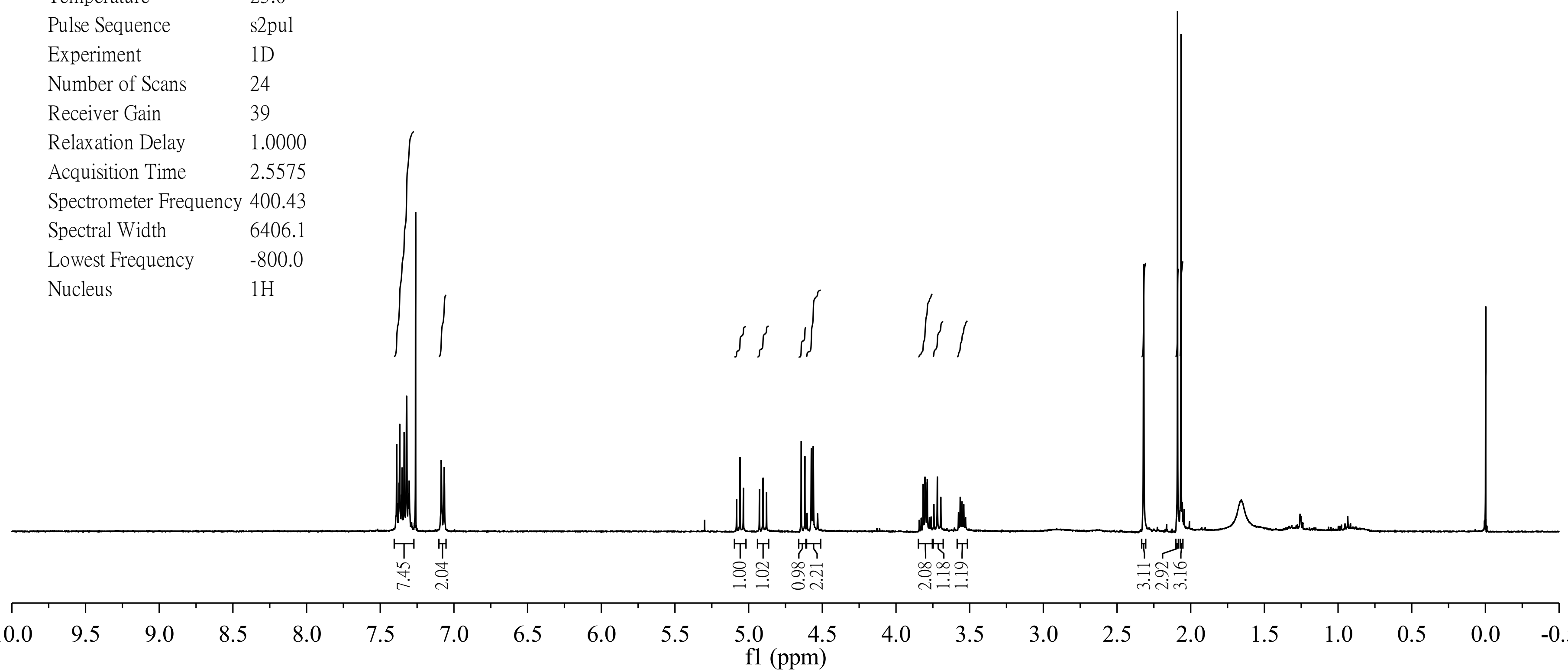

Parameter	value
Origin	Varian
Spectrometer	vnmrs
Solvent	cdcl3
Temperature	20.0
Pulse Sequence	s2pul
Experiment	1D
Number of Scans	80
Receiver Gain	30
Relaxation Delay	2.0000
Acquisition Time	1.3107
Spectrometer Frequency	100.53
Spectral Width	25000.0
Lowest Frequency	-1418.8
Nucleus	13C

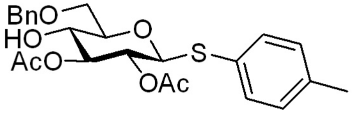

6b

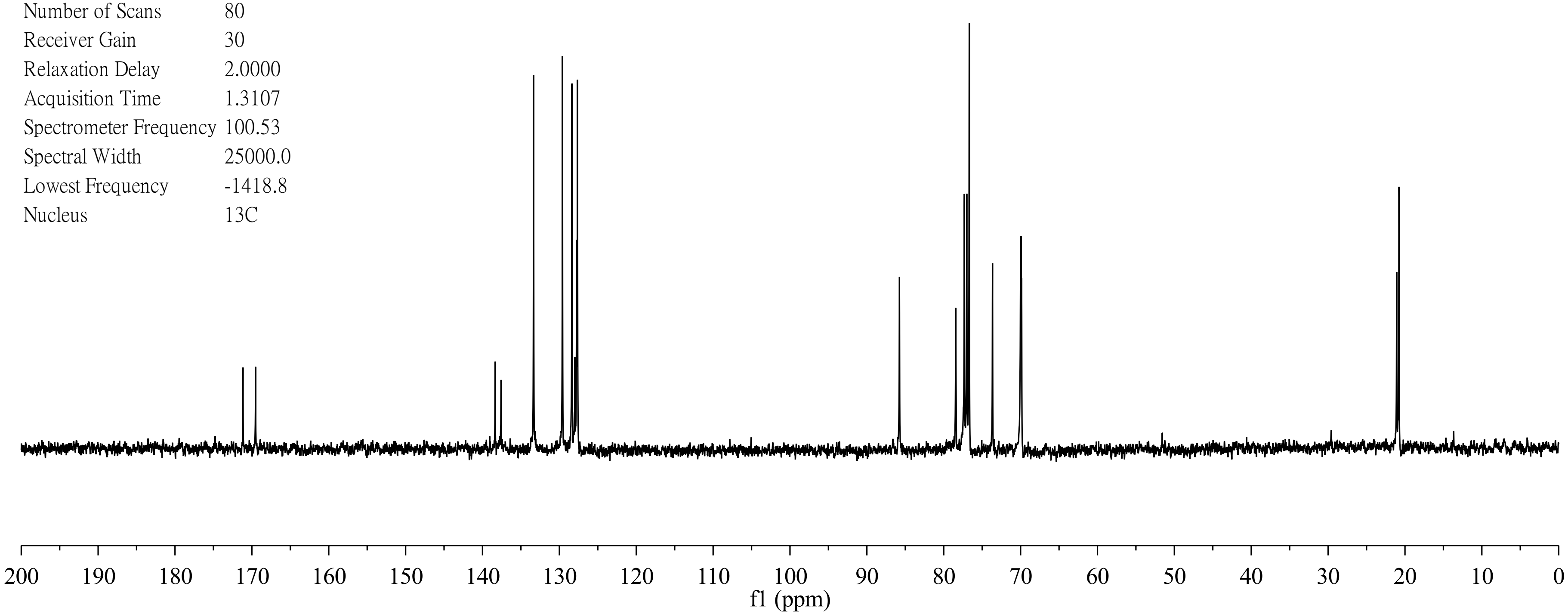

Parameter	value
Origin	Varian
Spectrometer	mercury
Solvent	cdcl3
Temperature	25.0
Pulse Sequence	s2pul
Experiment	1D
Number of Scans	64
Receiver Gain	39
Relaxation Delay	1.0000
Acquisition Time	2.5575
Spectrometer Frequency	400.43
Spectral Width	6406.1
Lowest Frequency	-799.6
Nucleus	1H

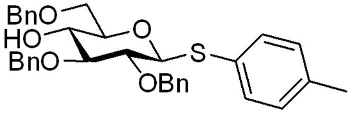

6c

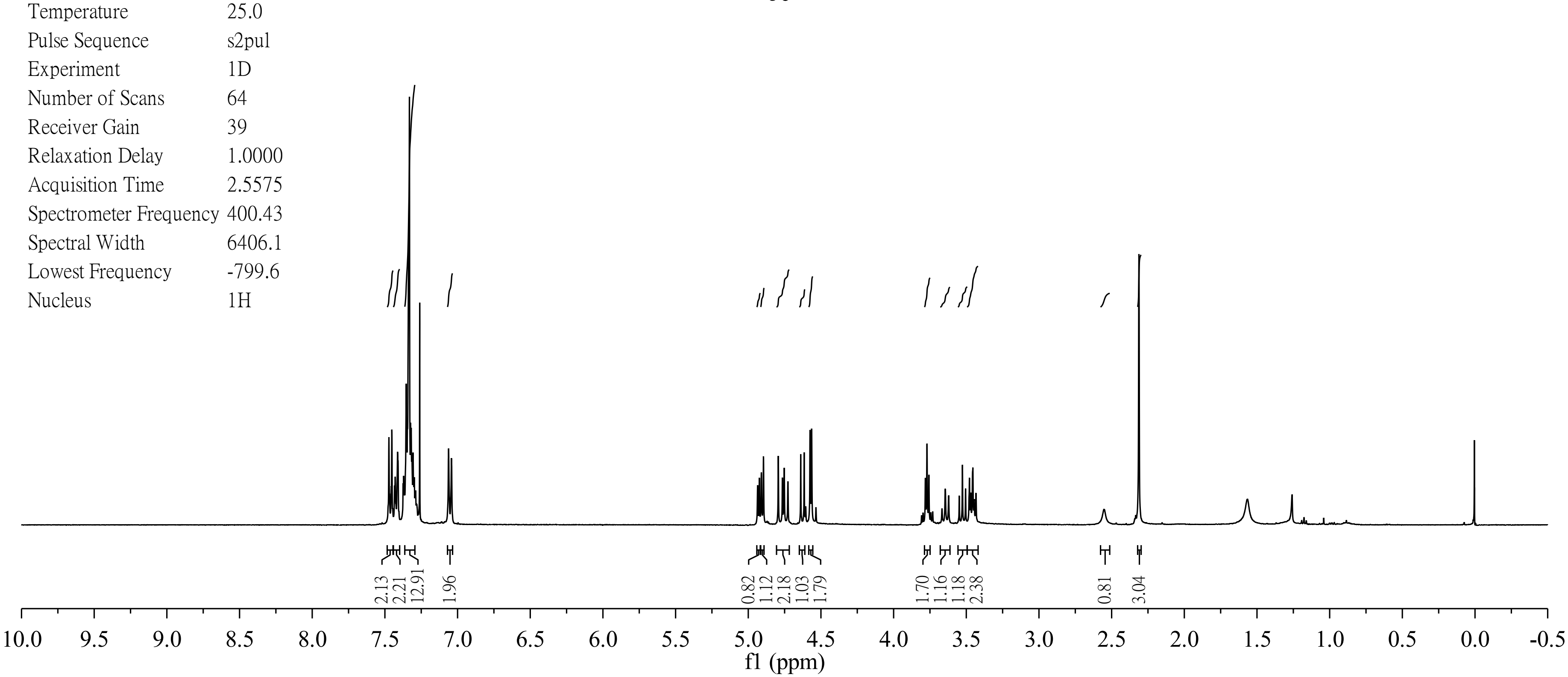

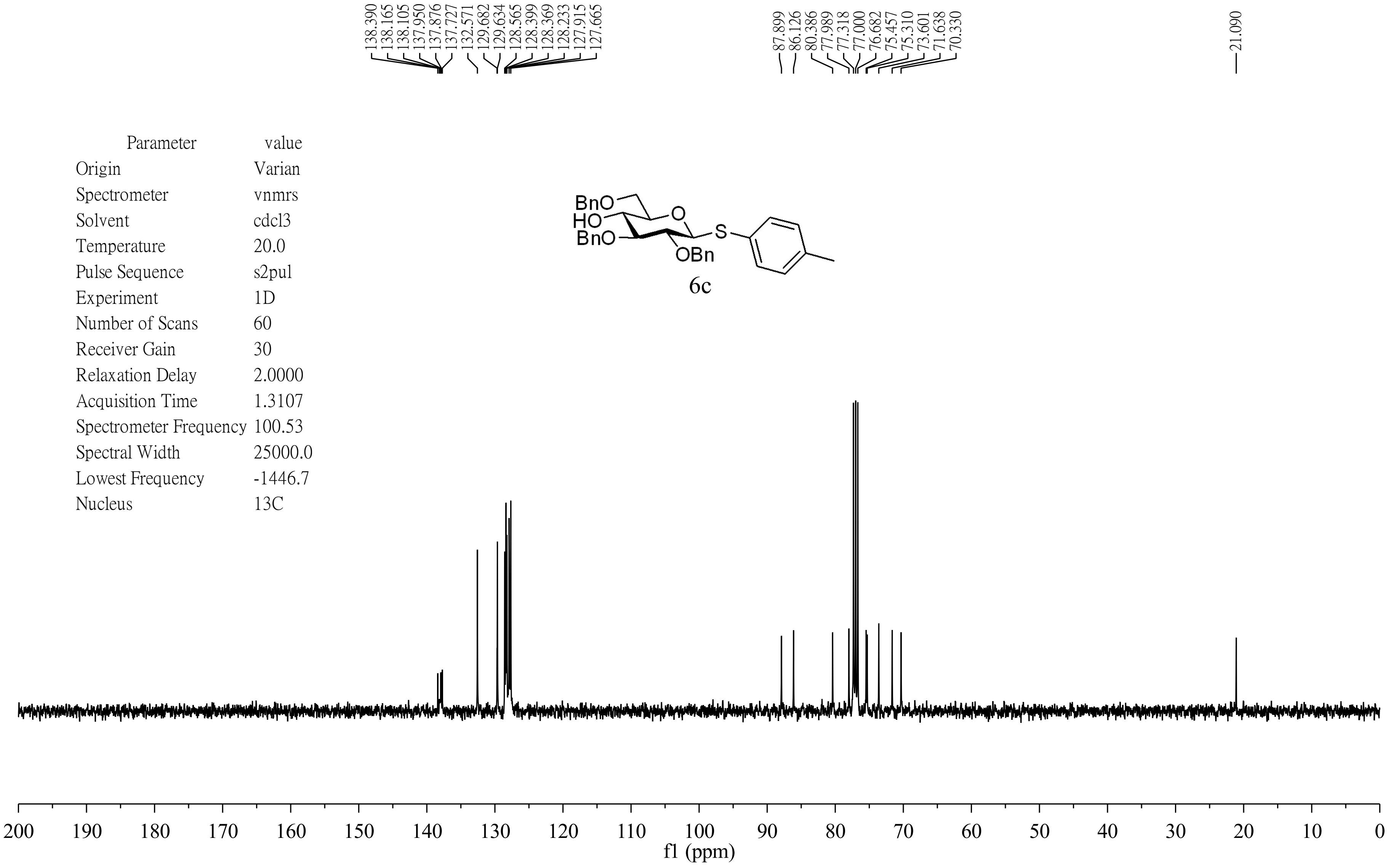

Parameter	value
Origin	Varian
Spectrometer	vnmrs
Solvent	cdcl3
Temperature	20.0
Pulse Sequence	s2pul
Experiment	1D
Number of Scans	60
Receiver Gain	30
Relaxation Delay	2.0000
Acquisition Time	1.3107
Spectrometer Frequency	100.53
Spectral Width	25000.0
Lowest Frequency	-1446.7
Nucleus	13C

6c

Parameter	value
Origin	Varian
Spectrometer	mercury
Solvent	cdcl3
Temperature	25.0
Pulse Sequence	s2pul
Experiment	1D
Number of Scans	72
Receiver Gain	39
Relaxation Delay	1.0000
Acquisition Time	2.5575
Spectrometer Frequency	400.43
Spectral Width	6406.1
Lowest Frequency	-800.7
Nucleus	1H

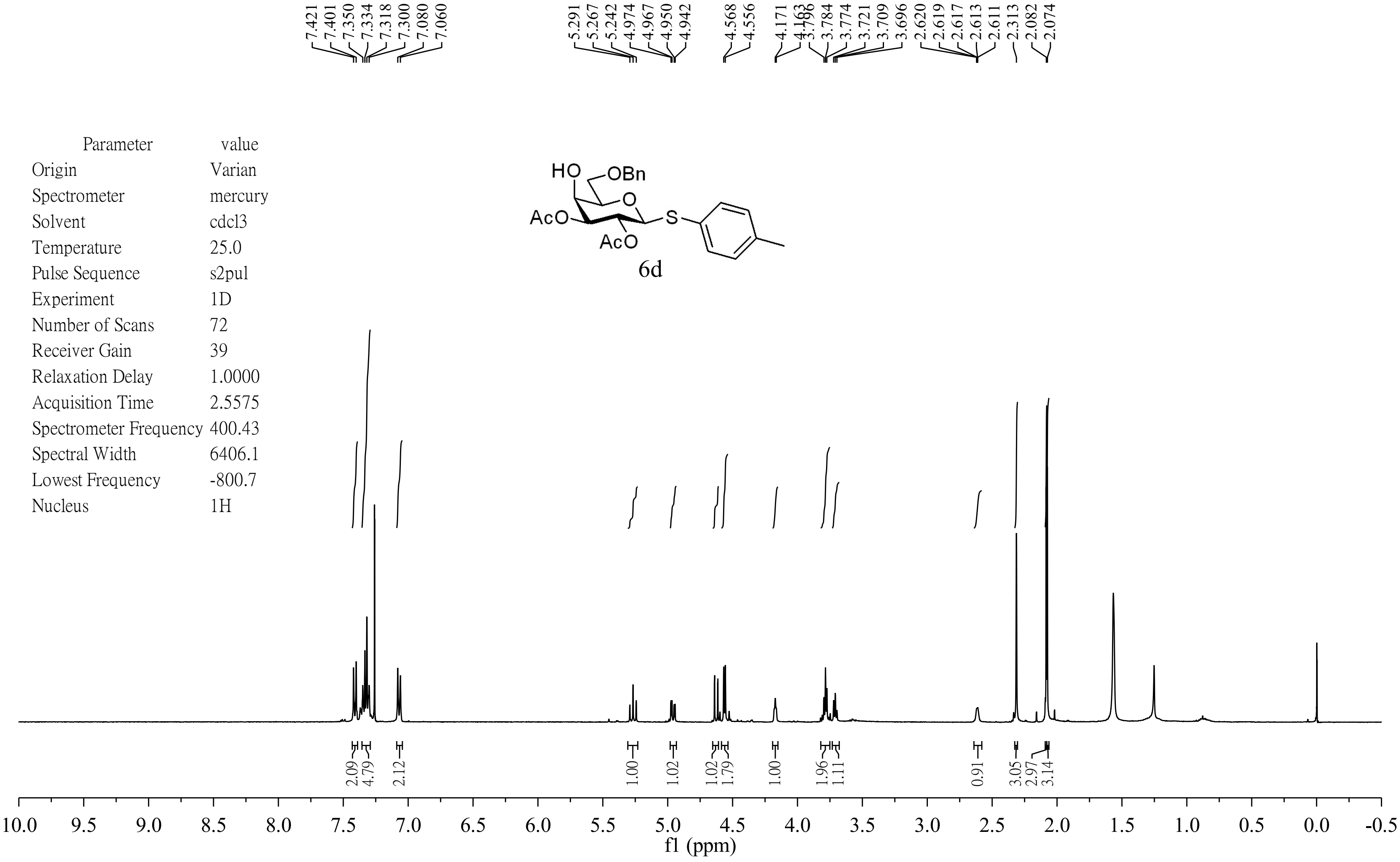

Parameter	value
Origin	Varian
Spectrometer	vnmrs
Solvent	cdcl3
Temperature	20.0
Pulse Sequence	s2pul
Experiment	1D
Number of Scans	448
Receiver Gain	30
Relaxation Delay	2.0000
Acquisition Time	1.3107
Spectrometer Frequency	100.53
Spectral Width	25000.0
Lowest Frequency	-1443.0
Nucleus	13C

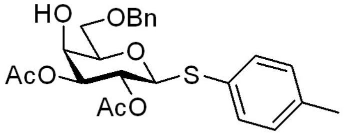

6d

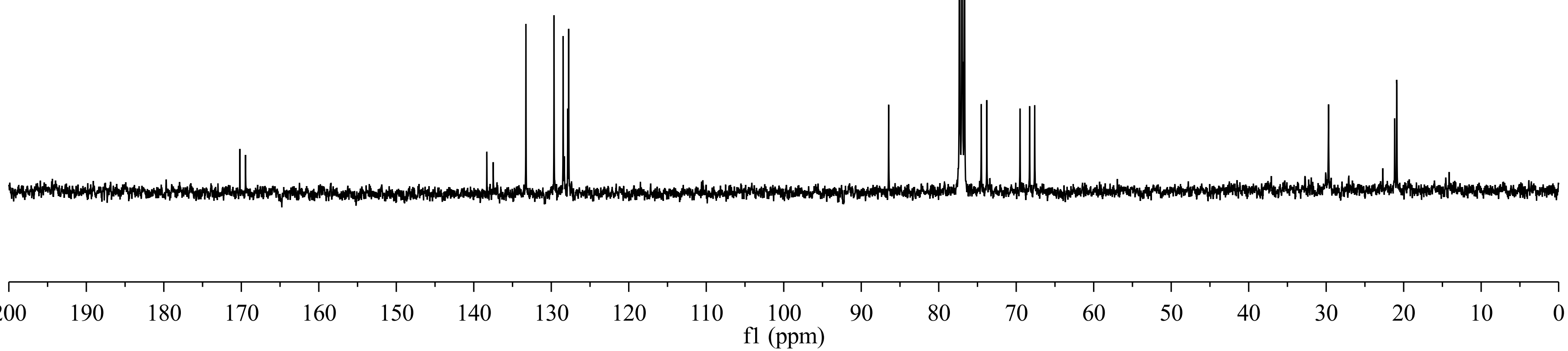

Parameter	value
Origin	Varian
Spectrometer	vnmrs
Solvent	cdcl3
Temperature	20.0
Pulse Sequence	s2pul
Experiment	1D
Number of Scans	40
Receiver Gain	30
Relaxation Delay	1.0000
Acquisition Time	2.5559
Spectrometer Frequency	399.76
Spectral Width	6410.3
Lowest Frequency	-799.4
Nucleus	1H

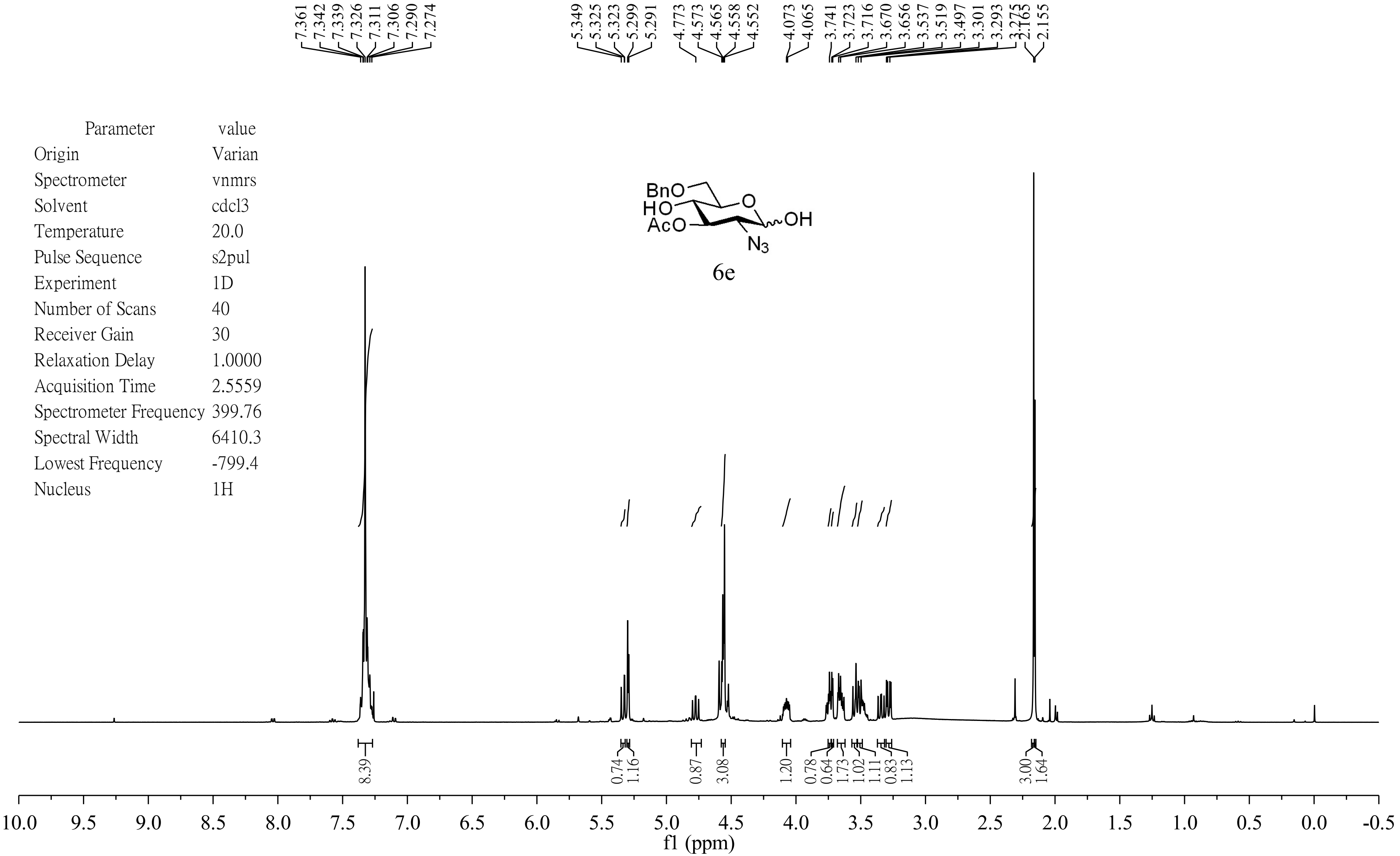

6e

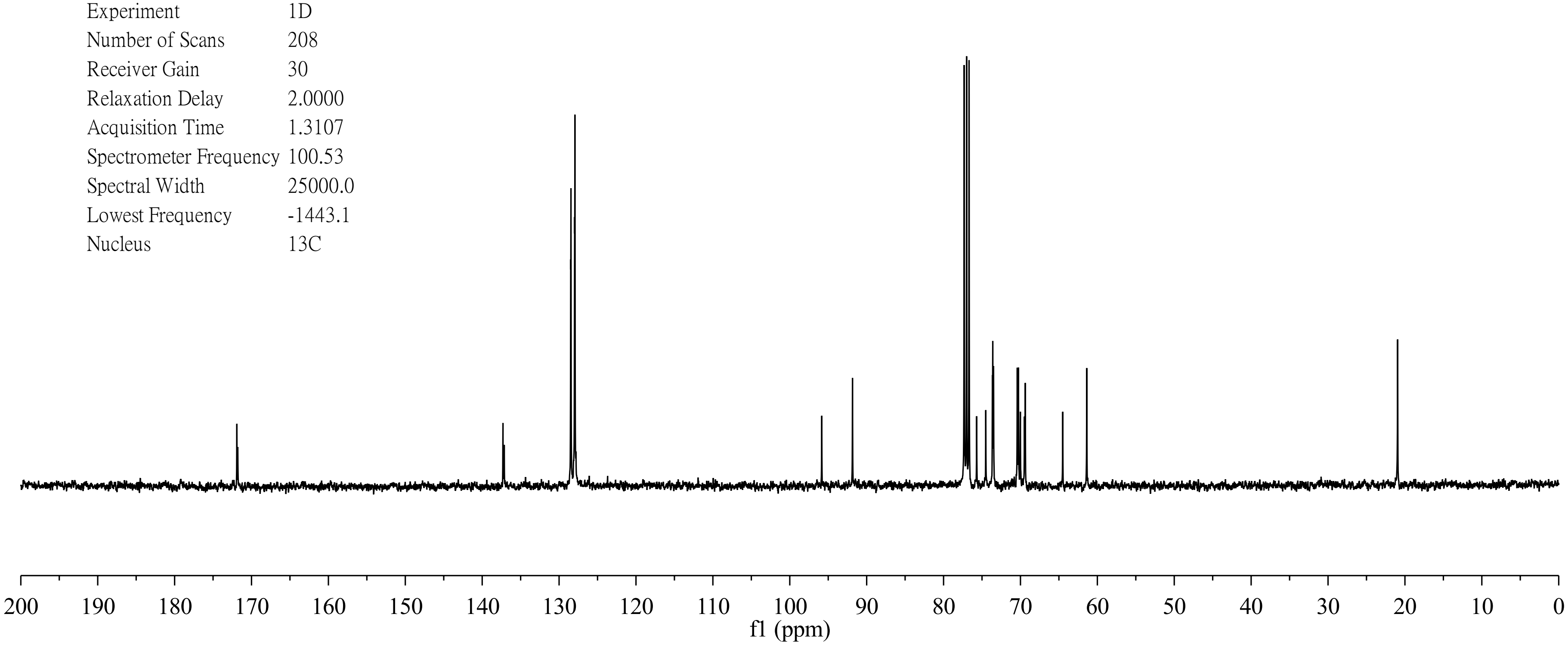

Parameter	value
Origin	Varian
Spectrometer	vnmrs
Solvent	cdcl3
Temperature	20.0
Pulse Sequence	s2pul
Experiment	1D
Number of Scans	208
Receiver Gain	30
Relaxation Delay	2.0000
Acquisition Time	1.3107
Spectrometer Frequency	100.53
Spectral Width	25000.0
Lowest Frequency	-1443.1
Nucleus	13C

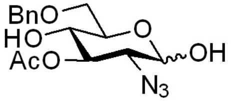

6e

Parameter	value
Origin	Varian
Spectrometer	vnmrs
Solvent	cdcl3
Temperature	20.0
Pulse Sequence	s2pul
Experiment	1D
Number of Scans	28
Receiver Gain	58
Relaxation Delay	1.0000
Acquisition Time	2.5559
Spectrometer Frequency	399.76
Spectral Width	6410.3
Lowest Frequency	-799.4
Nucleus	1H

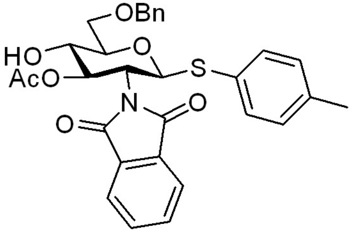

6f

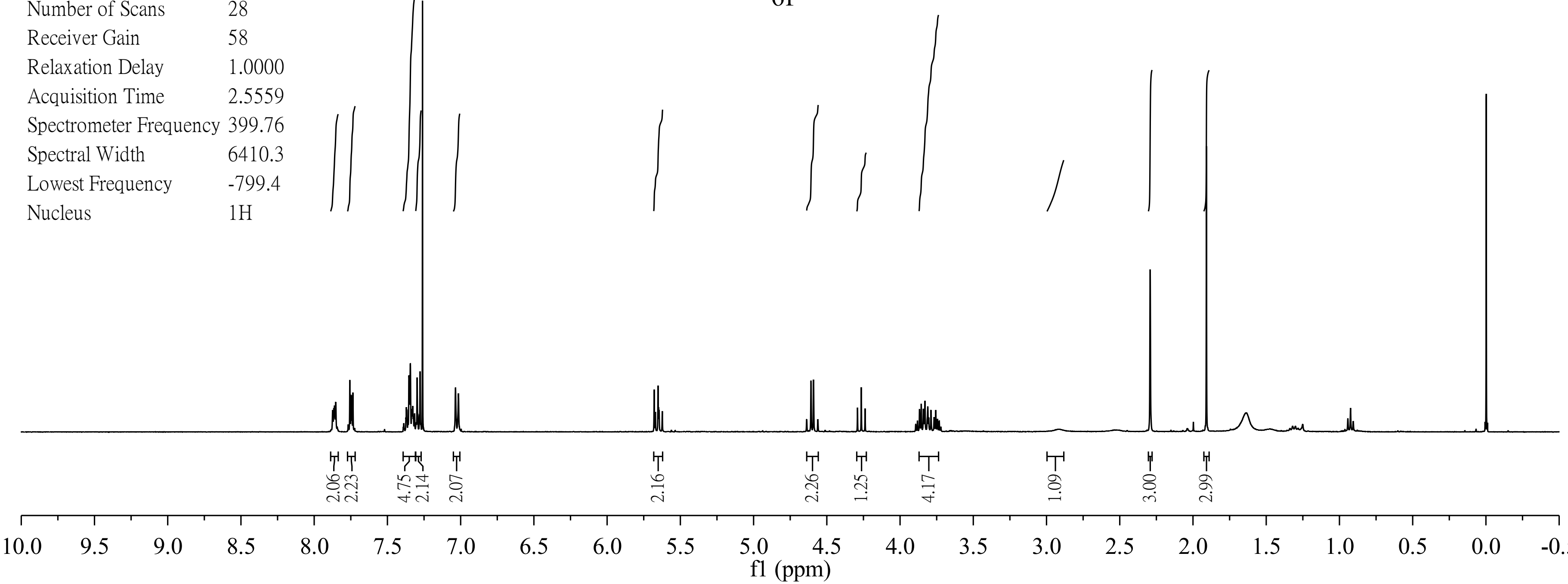

Parameter	value
Origin	Varian
Spectrometer	vnmrs
Solvent	cdcl3
Temperature	20.0
Pulse Sequence	s2pul
Experiment	1D
Number of Scans	240
Receiver Gain	30
Relaxation Delay	2.0000
Acquisition Time	1.3107
Spectrometer Frequency	100.53
Spectral Width	25000.0
Lowest Frequency	-1445.5
Nucleus	13C

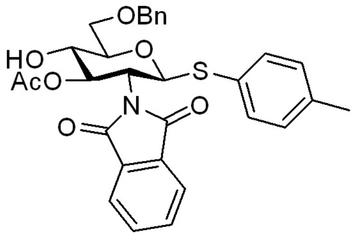

6f

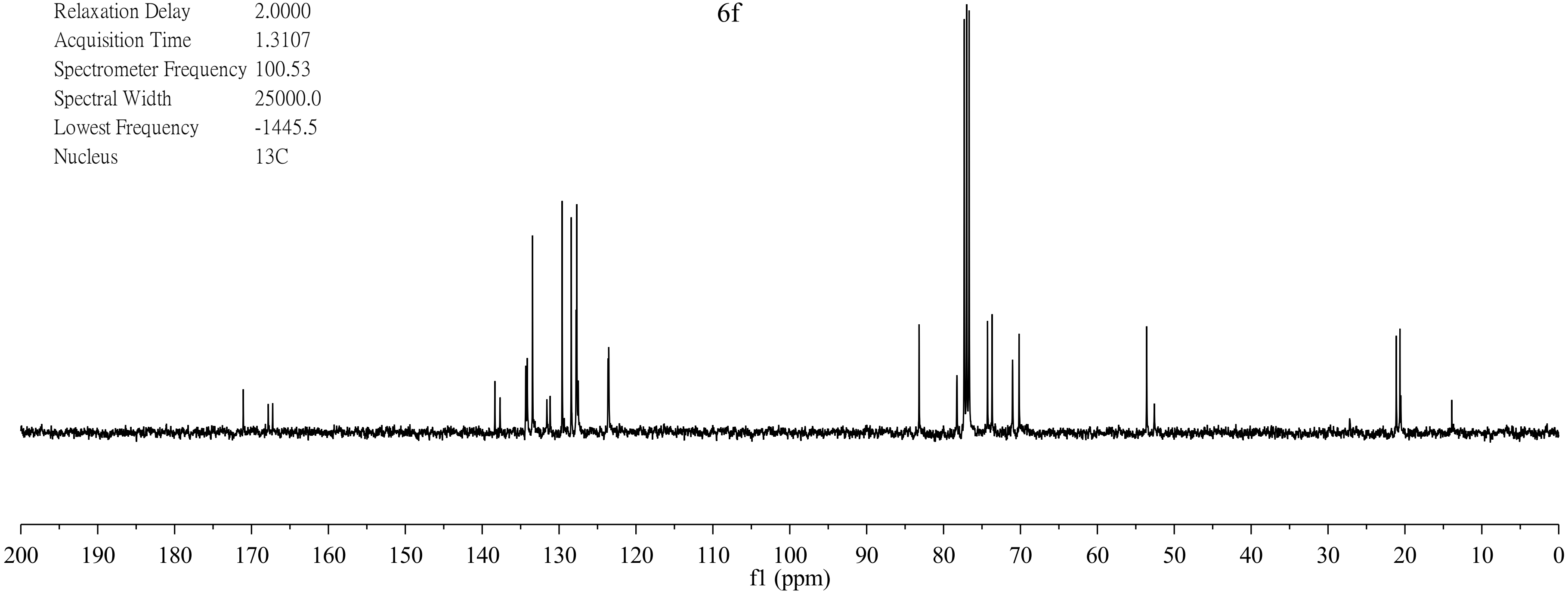

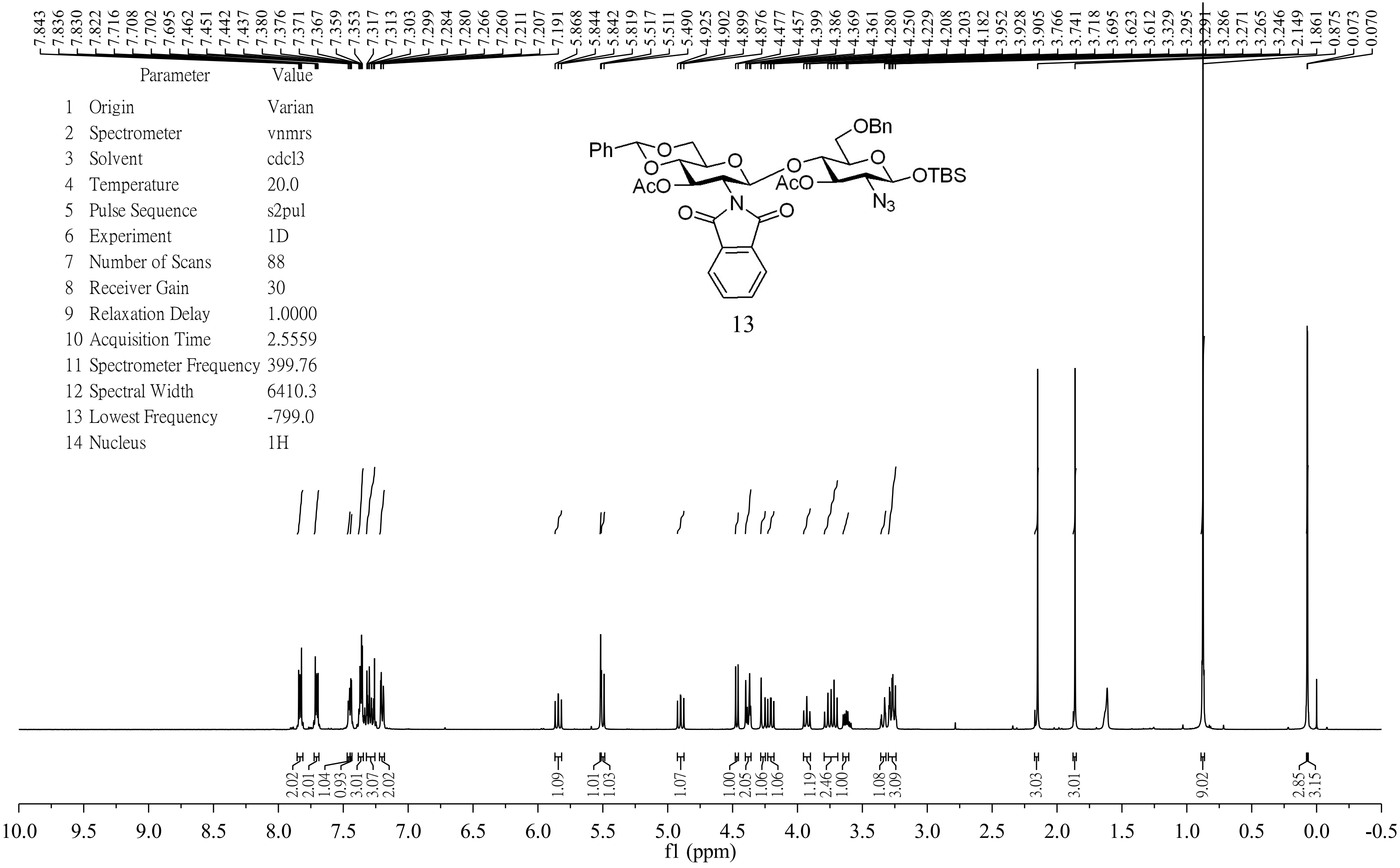

7.843 7.836 7.830 7.822 7.716 7.708 7.702 7.695 7.462 7.451 7.442 7.437 7.380 7.376 7.371 7.367 7.359 7.353 7.317 7.313 7.303 7.299 7.284 7.280 7.266 7.260 7.211 7.207 7.191 5.868 5.844 5.842 5.819 5.517 5.511 5.490 4.925 4.902 4.899 4.876 4.477 4.457 4.399 4.386 4.369 4.361 4.280 4.250 4.229 4.208 4.203 4.182 3.952 3.928 3.905 3.766 3.741 3.718 3.695 3.623 3.612 3.329 3.295 3.291 3.286 3.271 3.265 3.246 2.149 1.861 0.875 0.073 0.070
Parameter Value
1 Origin Varian
2 Spectrometer vnmrs
3 Solvent cdcl3
4 Temperature 20.0
5 Pulse Sequence s2pul
6 Experiment 1D
7 Number of Scans 88
8 Receiver Gain 30
9 Relaxation Delay 1.0000
10 Acquisition Time 2.5559
11 Spectrometer Frequency 399.76
12 Spectral Width 6410.3
13 Lowest Frequency -799.0
14 Nucleus 1H
Ph O O AcO O N O O O AcO O OBn OTBS N3
13
2.02 2.01 1.04 0.93 3.01 3.07 2.02 1.09 1.01 1.03 1.07 1.00 2.05 1.06 1.06 1.19 2.46 1.00 1.08 3.09 3.03 3.01 9.02 2.85 3.15
10.0 9.5 9.0 8.5 8.0 7.5 7.0 6.5 6.0 5.5 5.0 4.5 4.0 3.5 3.0 2.5 2.0 1.5 1.0 0.5 0.0 -0.5
f1 (ppm)

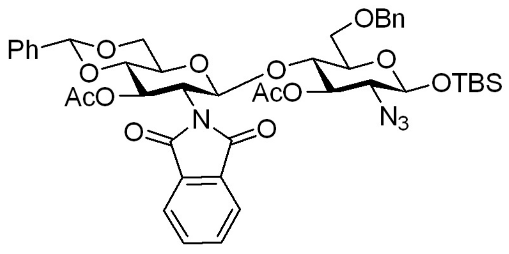

13

	Parameter	Value
1	Origin	Varian
2	Spectrometer	vnmrs
3	Solvent	cdcl3
4	Temperature	20.0
5	Pulse Sequence	s2pul
6	Experiment	1D
7	Number of Scans	2016
8	Receiver Gain	30
9	Relaxation Delay	2.0000
10	Acquisition Time	1.3107
11	Spectrometer Frequency	100.53
12	Spectral Width	25000.0
13	Lowest Frequency	-1441.8
14	Nucleus	13C

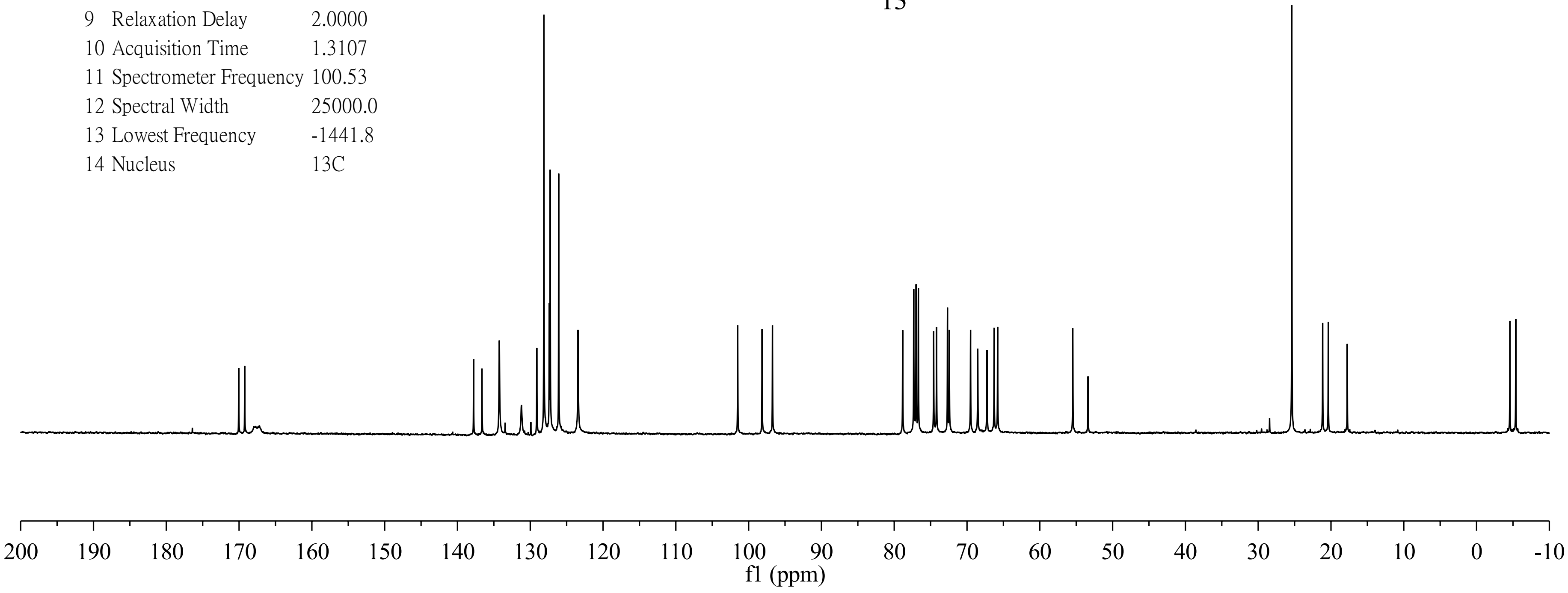

Parameters		
	Parameter	Value
1	Origin	Varian
2	Spectrometer	vnmrs
3	Solvent	cdcl3
4	Temperature	20.0
5	Pulse Sequence	s2pul
6	Experiment	1D
7	Number of Scans	92
8	Receiver Gain	30
9	Relaxation Delay	1.0000
10	Acquisition Time	2.5559
11	Spectrometer Frequency	399.76
12	Spectral Width	6410.3
13	Lowest Frequency	-791.2
14	Nucleus	1H

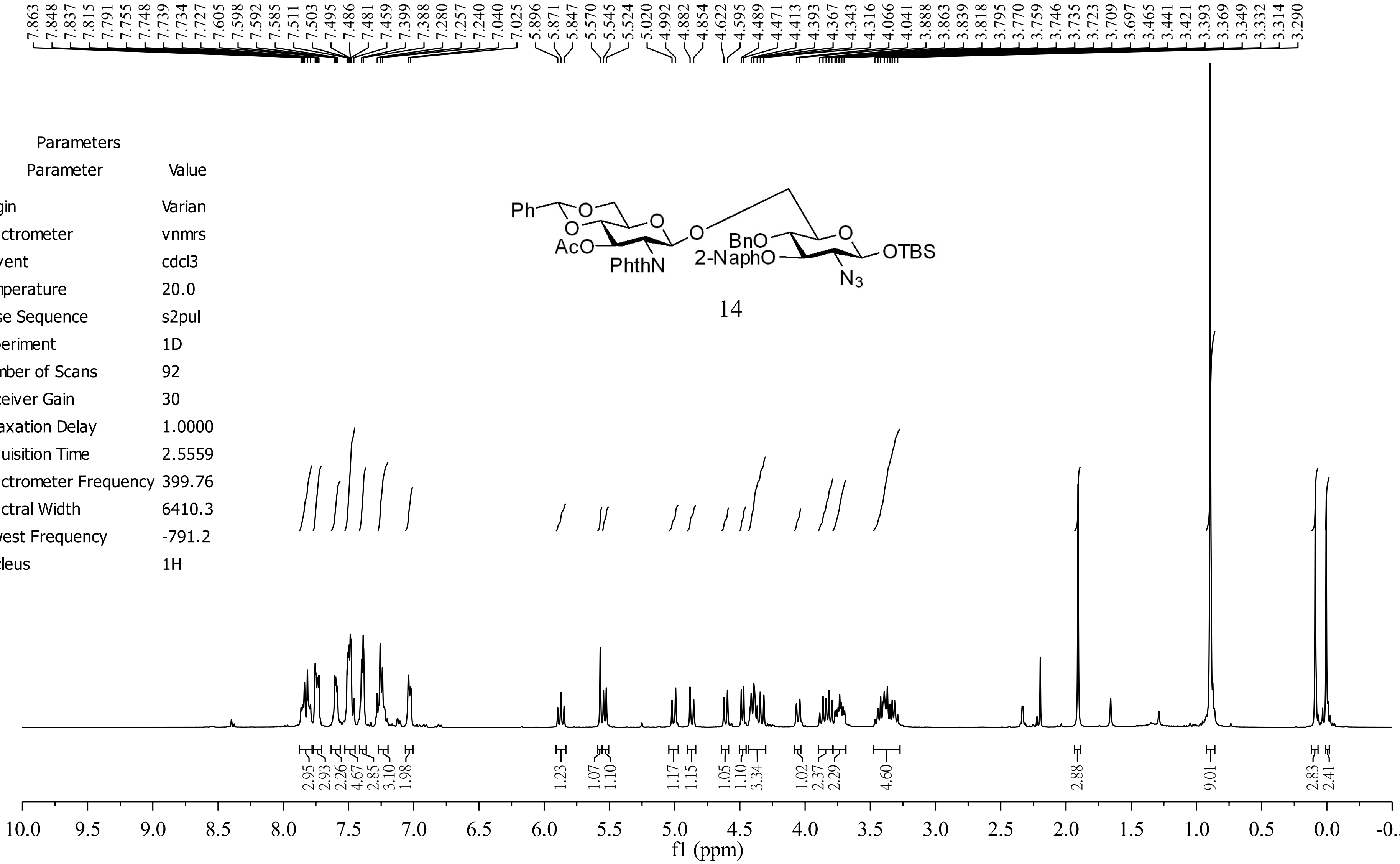

Parameters

Parameter		Value
1	Origin	Varian
2	Spectrometer	vnmrs
3	Solvent	cdcl3
4	Temperature	20.0
5	Pulse Sequence	s2pul
6	Experiment	1D
7	Number of Scans	1360
8	Receiver Gain	30
9	Relaxation Delay	2.0000
10	Acquisition Time	1.3107
11	Spectrometer Frequency	100.53
12	Spectral Width	25000.0
13	Lowest Frequency	-1443.1
14	Nucleus	13C

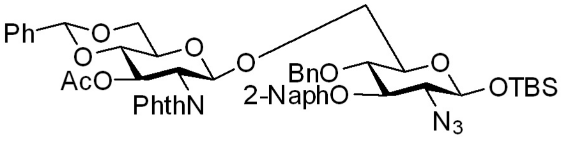

14

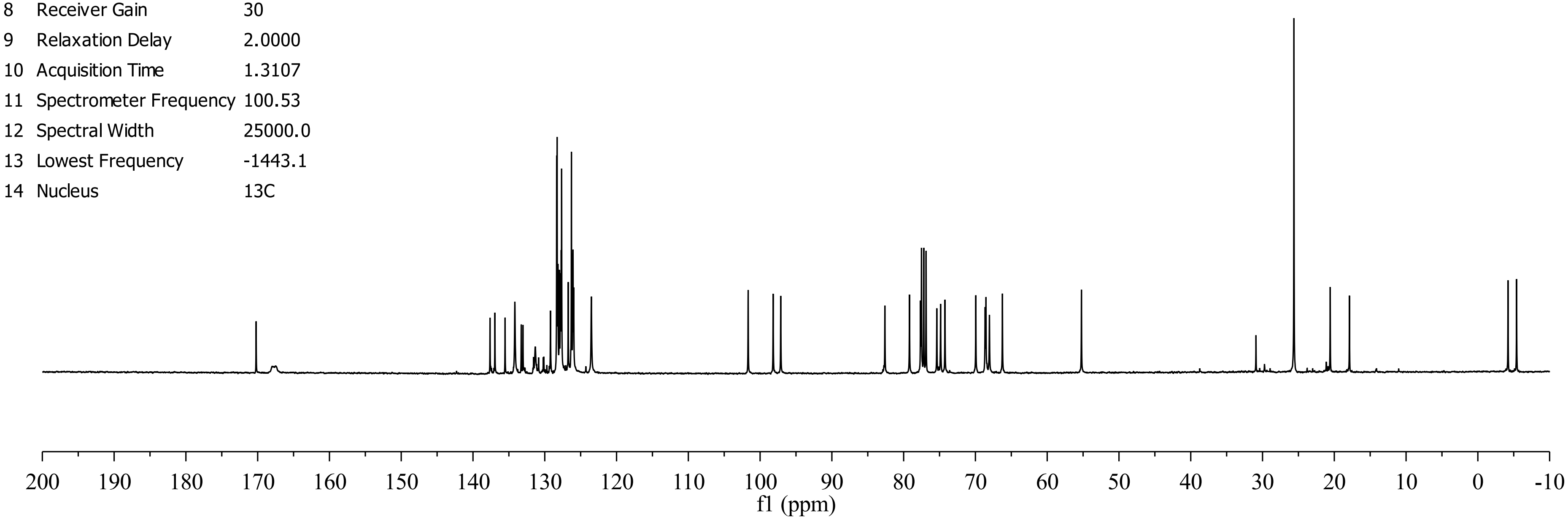

Parameter	value
Origin	Varian
Spectrometer	mercury
Solvent	CDCl3
Temperature	29.0
Pulse Sequence	s2pul
Experiment	1D
Number of Scans	80
Receiver Gain	20
Relaxation Delay	0.0000
Acquisition Time	2.8000
Spectrometer Frequency	400.44
Spectral Width	6006.0
Lowest Frequency	-476.4
Nucleus	1H

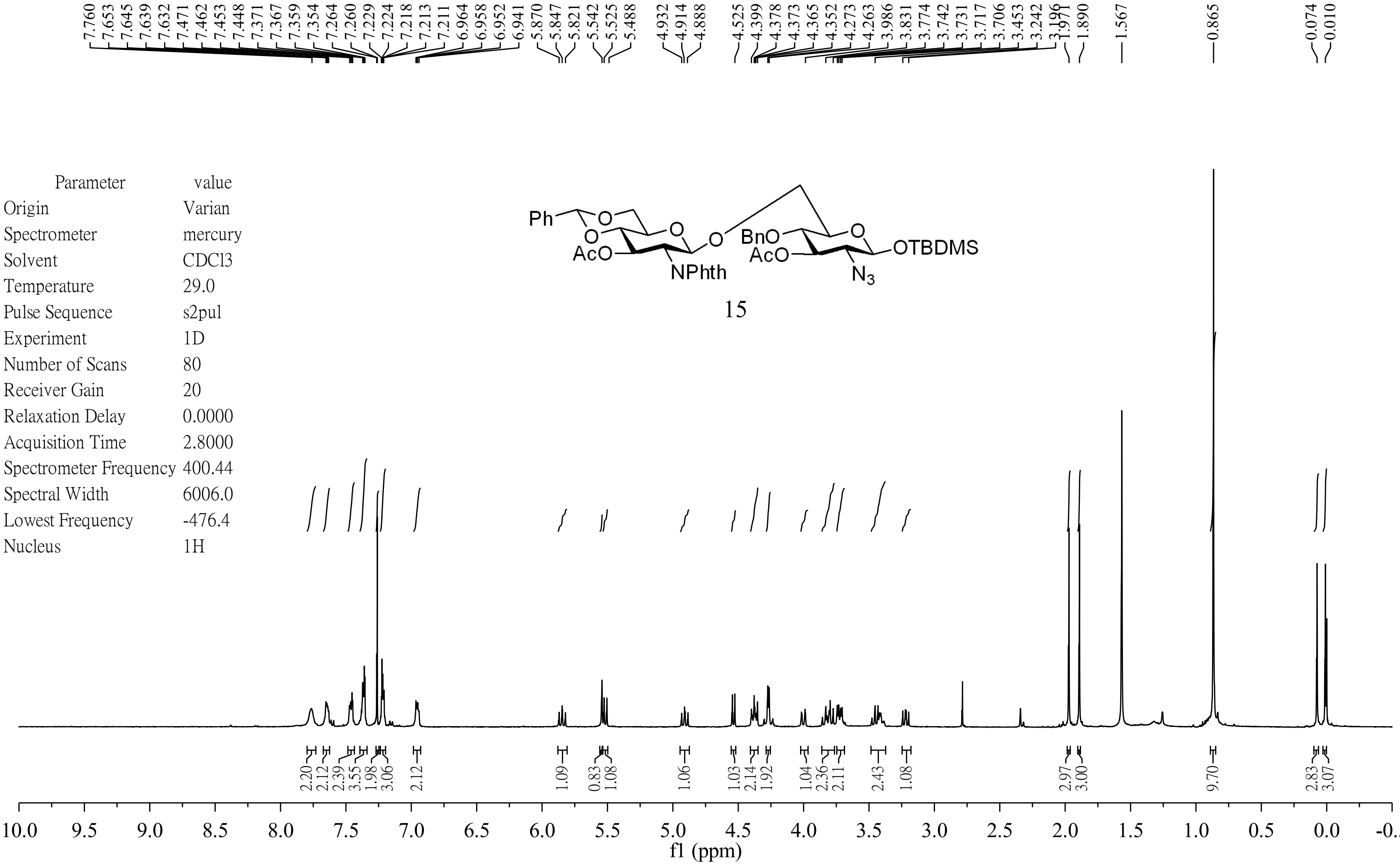

Parameter	value
Origin	Varian
Spectrometer	vnmrs
Solvent	cdcl3
Temperature	20.0
Pulse Sequence	s2pul
Experiment	1D
Number of Scans	612
Receiver Gain	30
Relaxation Delay	2.0000
Acquisition Time	1.3107
Spectrometer Frequency	100.53
Spectral Width	25000.0
Lowest Frequency	-1443.2
Nucleus	13C

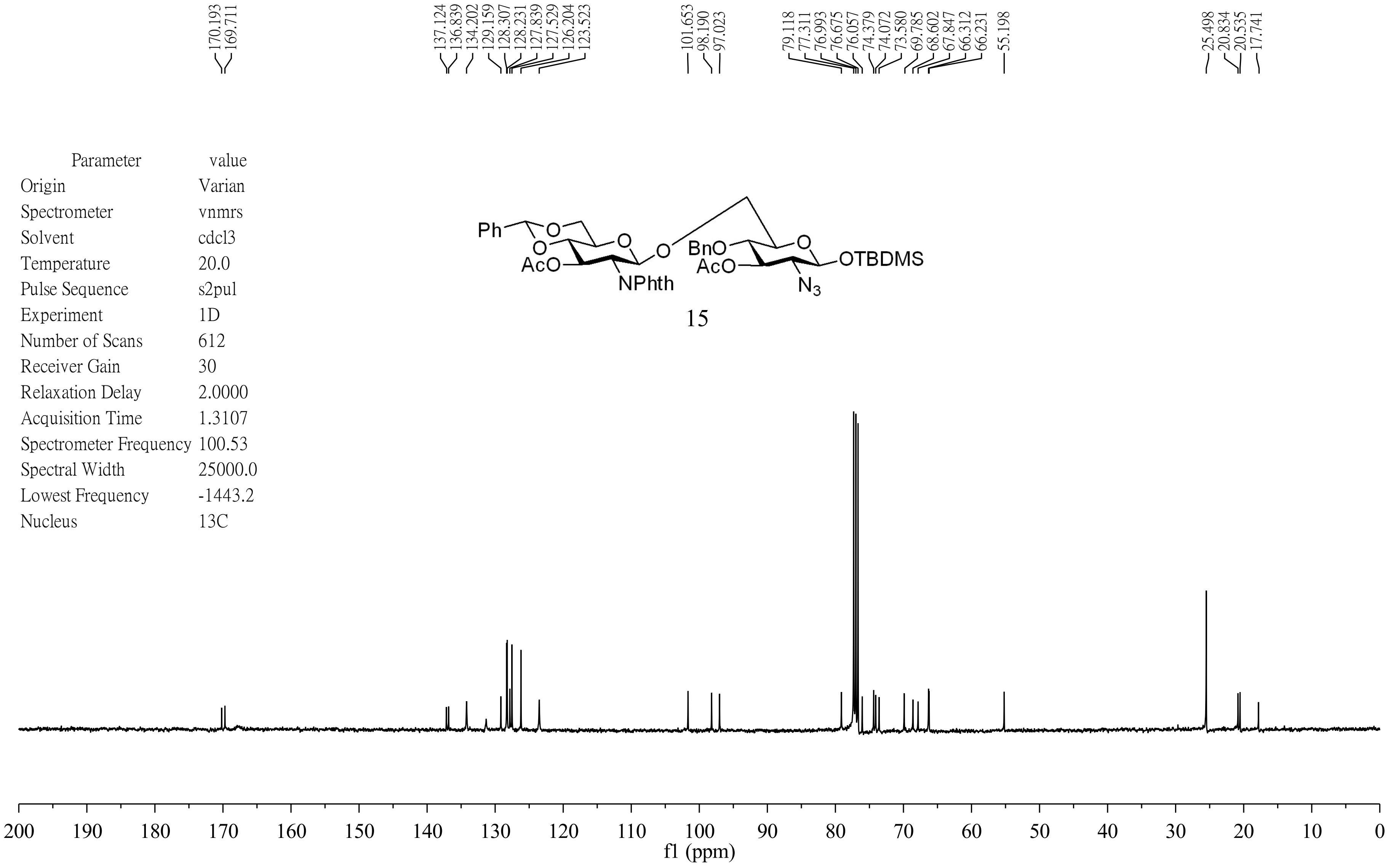

Parameter	Value
1 Origin	Varian
2 Spectrometer	vnmrs
3 Solvent	cdcl3
4 Temperature	20.0
5 Pulse Sequence	s2pul
6 Experiment	1D
7 Number of Scans	52
8 Receiver Gain	18
9 Relaxation Delay	1.0000
10 Acquisition Time	2.5559
11 Spectrometer Frequency	399.76
12 Spectral Width	6410.3
13 Lowest Frequency	-797.8
14 Nucleus	1H

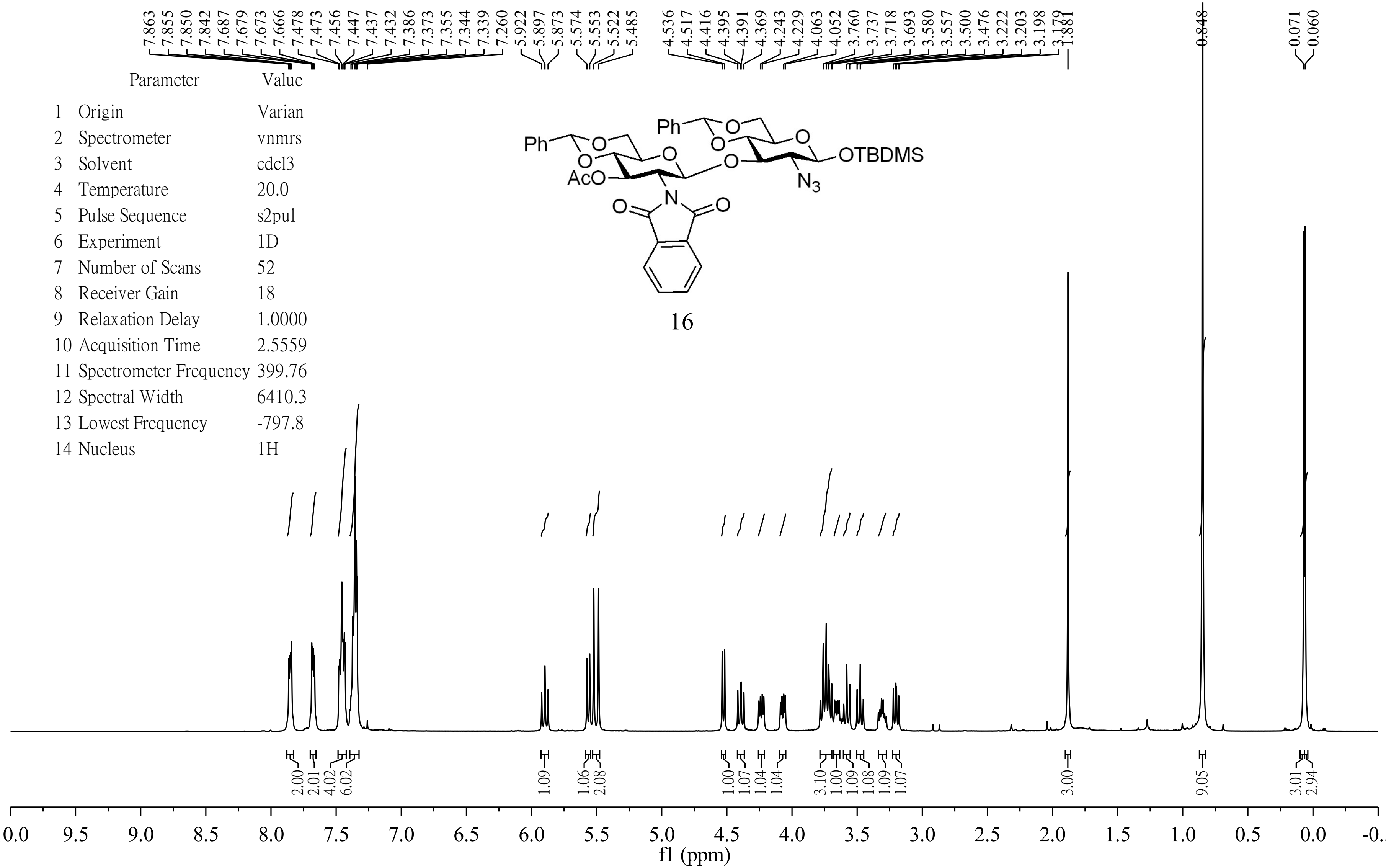

Parameter	Value
1 Origin	Varian
2 Spectrometer	vnmrs
3 Solvent	cdcl3
4 Temperature	20.0
5 Pulse Sequence	s2pul
6 Experiment	1D
7 Number of Scans	440
8 Receiver Gain	30
9 Relaxation Delay	2.0000
10 Acquisition Time	1.3107
11 Spectrometer Frequency	100.53
12 Spectral Width	25000.0
13 Lowest Frequency	-1454.3
14 Nucleus	13C

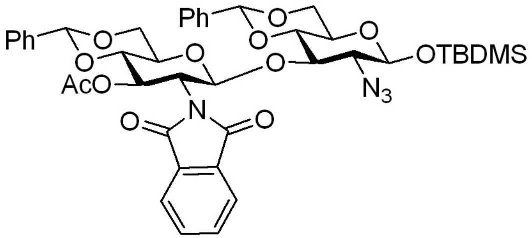

16

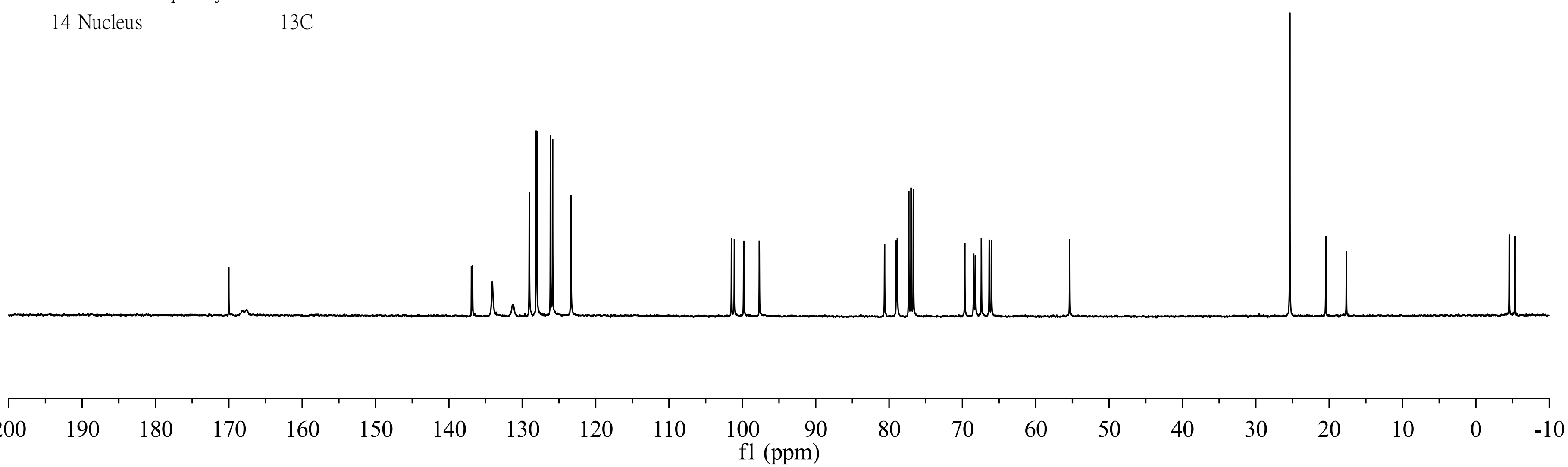

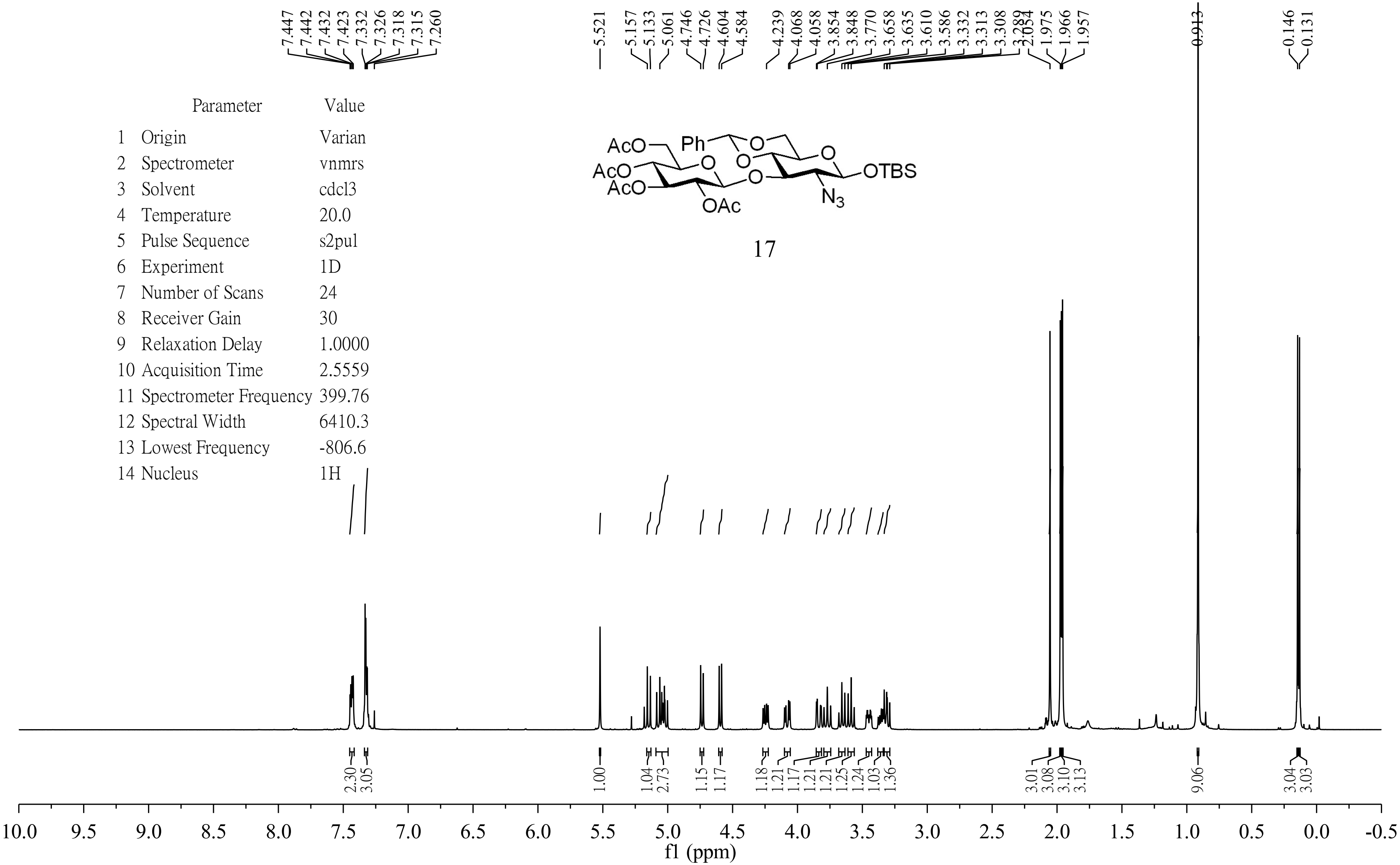
7.447 7.442 7.432 7.423 7.332 7.326 7.318 7.315 7.260
5.521 5.157 5.133 5.061 4.746 4.726 4.604 4.584 4.239 4.068 4.058 3.854 3.848 3.770 3.658 3.635 3.610 3.586 3.332 3.313 3.308 3.289 2.054 1.975 1.966 1.957
0.913
0.146 0.131
Parameter Value
1 Origin Varian
2 Spectrometer vnmrs
3 Solvent cdcl3
4 Temperature 20.0
5 Pulse Sequence s2pul
6 Experiment 1D
7 Number of Scans 24
8 Receiver Gain 30
9 Relaxation Delay 1.0000
10 Acquisition Time 2.5559
11 Spectrometer Frequency 399.76
12 Spectral Width 6410.3
13 Lowest Frequency -806.6
14 Nucleus 1H
AcO
AcO
AcO
Ph
O
OAc
N3
OTBS
17
2.30 3.05 1.00 1.04 2.73 1.15 1.17 1.18 1.21 1.17 1.21 1.21 1.25 1.24 1.03 1.36 3.01 3.08 3.10 3.13 9.06 3.04 3.03
10.0 9.5 9.0 8.5 8.0 7.5 7.0 6.5 6.0 5.5 5.0 4.5 4.0 3.5 3.0 2.5 2.0 1.5 1.0 0.5 0.0 -0.5
f1 (ppm)

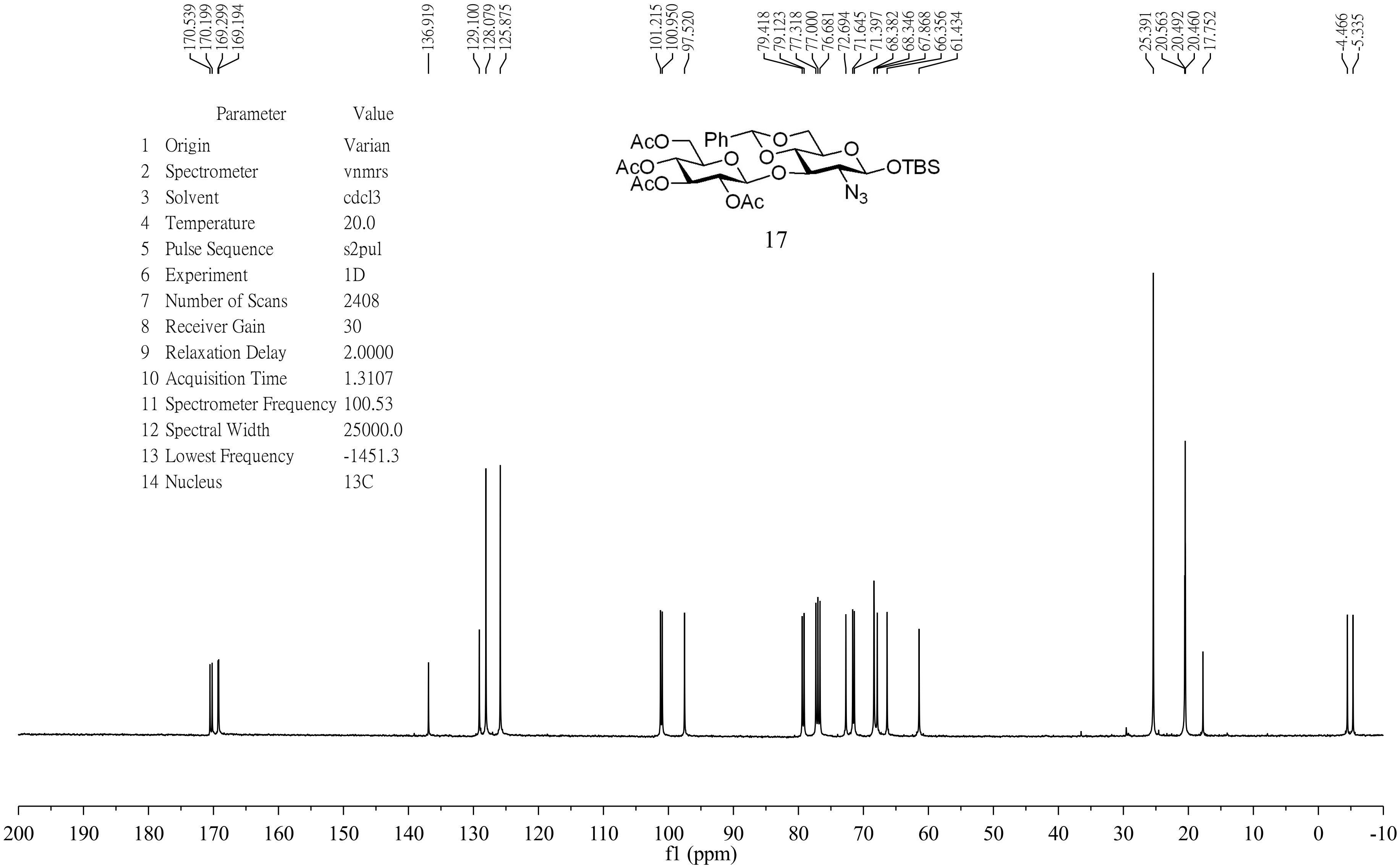

	Parameter	Value
1	Origin	Varian
2	Spectrometer	vnmrs
3	Solvent	cdcl3
4	Temperature	20.0
5	Pulse Sequence	s2pul
6	Experiment	1D
7	Number of Scans	2408
8	Receiver Gain	30
9	Relaxation Delay	2.0000
10	Acquisition Time	1.3107
11	Spectrometer Frequency	100.53
12	Spectral Width	25000.0
13	Lowest Frequency	-1451.3
14	Nucleus	13C

17
